# Identification of prostate cancer biomarkers in urinary exosomes

**DOI:** 10.18632/oncotarget.4851

**Published:** 2015-07-13

**Authors:** Anders Øverbye, Tore Skotland, Christian J. Koehler, Bernd Thiede, Therese Seierstad, Viktor Berge, Kirsten Sandvig, Alicia Llorente

**Affiliations:** ^1^ Department of Molecular Cell Biology, Institute for Cancer Research, Oslo University Hospital-The Norwegian Radium Hospital, Oslo, Norway; ^2^ Centre for Cancer Biomedicine, Faculty of Medicine, University of Oslo, Oslo, Norway; ^3^ The Biotechnology Centre of Oslo, University of Oslo, Oslo, Norway; ^4^ Department of Biosciences, University of Oslo, Oslo, Norway; ^5^ Department of Radiology and Nuclear Medicine, Oslo University Hospital, Oslo, Norway; ^6^ Department of Urology, Oslo University Hospital, Oslo, Norway

**Keywords:** biomarkers, exosomes, extracellular vesicles, mass spectrometry, prostate cancer

## Abstract

Exosomes have recently appeared as a novel source of non-invasive cancer biomarkers since tumour-specific molecules can be found in exosomes isolated from biological fluids. We have here investigated the proteome of urinary exosomes by using mass spectrometry to identify proteins differentially expressed in prostate cancer patients compared to healthy male controls. In total, 15 control and 16 prostate cancer samples of urinary exosomes were analyzed. Importantly, 246 proteins were differentially expressed in the two groups. The majority of these proteins (221) were up-regulated in exosomes from prostate cancer patients. These proteins were analyzed according to specific criteria to create a focus list that contained 37 proteins. At 100% specificity, 17 of these proteins displayed individual sensitivities above 60%. Even though several of these proteins showed high sensitivity and specificity for prostate cancer as individual biomarkers, combining them in a multi-panel test has the potential for full differentiation of prostate cancer from non-disease controls. The highest sensitivity, 94%, was observed for transmembrane protein 256 (TM256; chromosome 17 open reading frame 61). LAMTOR proteins were also distinctly enriched with very high specificity for patient samples. TM256 and LAMTOR1 could be used to augment the sensitivity to 100%. Other prominent proteins were V-type proton ATPase 16 kDa proteolipid subunit (VATL), adipogenesis regulatory factor (ADIRF), and several Rab-class members and proteasomal proteins. In conclusion, this study clearly shows the potential of using urinary exosomes in the diagnosis and clinical management of prostate cancer.

## INTRODUCTION

Prostate cancer is the sixth leading cause of cancer-related deaths in males world-wide [1]. Some patients will never experience symptoms or disease progression, whereas others will have a rapid progression to a life-threatening disease [1]. Existing clinical markers like tumor stage, Gleason score and prostate specific antigen (PSA) blood level, are not sufficient to guide choice of treatment, and many patients face problems with under- or overtreatment [2]. PSA has been used for nearly three decades as a biomarker for prostate cancer and is still a useful marker for prostate cancer after diagnosis [3]. However, the use of PSA has resulted in significant prostate cancer over-diagnosis [4] since elevated serum PSA levels are often detected in patients with non-malignant conditions such as benign prostatic hyperplasia. It is therefore clear that characterization of additional biomarkers that can indicate whether a histologically proven tumor will give rise to a clinical significant disease is strongly needed. Importantly, several prostate biomarkers such as PCA3, TMPRSS2:ERG and AMCAR have recently been identified [5, 6].

Extracellular vesicles (EVs) released by cells have recently appeared as a novel source of noninvasive biomarkers for several diseases [7-9]. In terms of cancer, this is based on the idea that EVs released by tumor cells contain a set of specific tumor-related molecules that can be found in biological fluids such as blood, urine, seminal fluid and breast milk [10, 11]. Interestingly, several proteins [12-15], lipids [16], RNAs [7] and microRNAs [17, 18] present in EVs have been identified as potential prostate cancer biomarkers.

Blood has traditionally been the dominant body fluid for cancer biomarkers such as PSA (prostate cancer), CA-125 (ovarian cancer), and HER2/neu (breast cancer). However, the use of urine is increasing in the cancer biomarker field since this bio-fluid is obtained noninvasively and readily in large quantities [19]. One of the immediate challenges of mass spectrometry (MS) analysis in blood (serum/plasma) samples is the vast difference in dynamic range of proteins, that even when the most abundant proteins are removed (ie. albumin, micro-globulin), makes the identification of low abundant proteins difficult. This is a less prominent feature in urine samples. Furthermore, urine has additional advantages when it comes to cancers of the urogenital system since the composition of urine directly reflects changes in associated organs functioning. The presence of EVs in urine was discovered in 2004 [20]. It is believed that urinary EVs originate from epithelial cells of the urogenital system, which includes the organs involved in reproduction and urine excretion. It is at the moment unclear to which extent the different organs of this system contribute to the urine EV population. However, several studies indicate that EVs originate to some extent from prostate cells since several prostate-specific molecules such as prostatic acid phosphatase (PPAP), prostate transglutaminase (TGM4) and prostate-specific membrane antigen (PSMA) have been detected in urinary EVs [7, 12, 21-23]. In several of these studies, urine was collected after a prostate massage since this procedure seems to increase the amount of exosomes found in urine [7, 24]. However, in the study presented here urine was collected directly, without a previous prostate massage, due to practical reasons. Crucially, an easy sample collection procedure would facilitate the use of potential EV-based prostate cancer markers in the clinic.

Living cells release different types of EVs. The two main types of EVs have different mechanisms of release: direct budding from the plasma membrane and fusion of multivesicular bodies (MVBs) with the plasma membrane, a process that leads to the release of the internal vesicles contained in the MVBs [25, 26]. EVs that form directly from the plasma membrane are often referred to as shedding vesicles or microparticles. These vesicles are 100-1000 nm in diameter and are normally sedimented at 10,000g [27]. EVs that originate from MVBs are commonly named exosomes, have a size diameter of 30-150 nm and are normally sedimented at 100,000 [27]. There are at the moment no experimental methods that allow the complete separation of these two types of EVs. However, sequential centrifugation and ultracentrifugation at 100,000 x g is a method commonly used to obtain an EV-pellet enriched in exosomes. Since this is the method that we have used here, and several of our analyses support the idea that we have isolated exosomes, we will use this term to refer to the vesicles investigated in this study.

**Figure 1:**
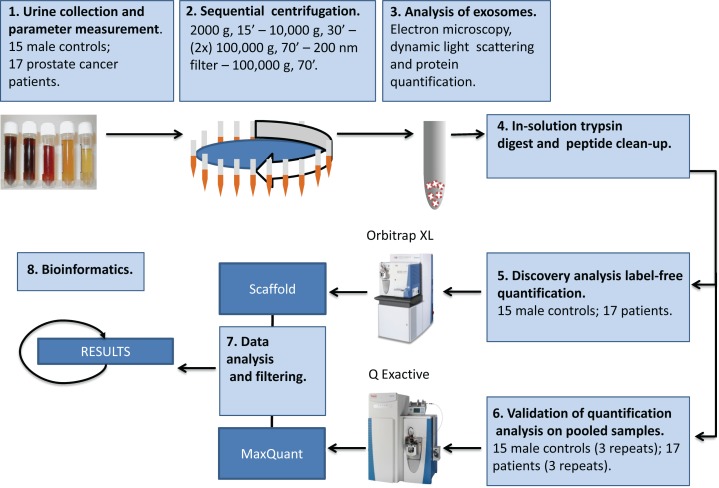
Schematic procedure for isolation and characterization of urinary exosomes See text for details.

Exosomes released by cancer cell lines are commonly chosen as a first step in the search of biomarkers for a specific cancer type. We have recently characterized the composition of exosomes released by the metastatic prostate cancer cell line PC-3 at the protein [13], lipid [16] and microRNA level [18]. In order to further investigate the use of exosomes as a source of prostate cancer biomarkers, we have here isolated urinary exosomes and performed a proteomic analysis of urinary exosomes from prostate cancer patients and healthy controls (Figure 1). Intriguingly, 246 proteins were found to be significantly changed in urinary exosomes from prostate cancer patients versus healthy controls. The potential use of these proteins as prostate cancer biomarkers is discussed.

## RESULTS

### Isolation of urinary exosomes

**Figure 2:**
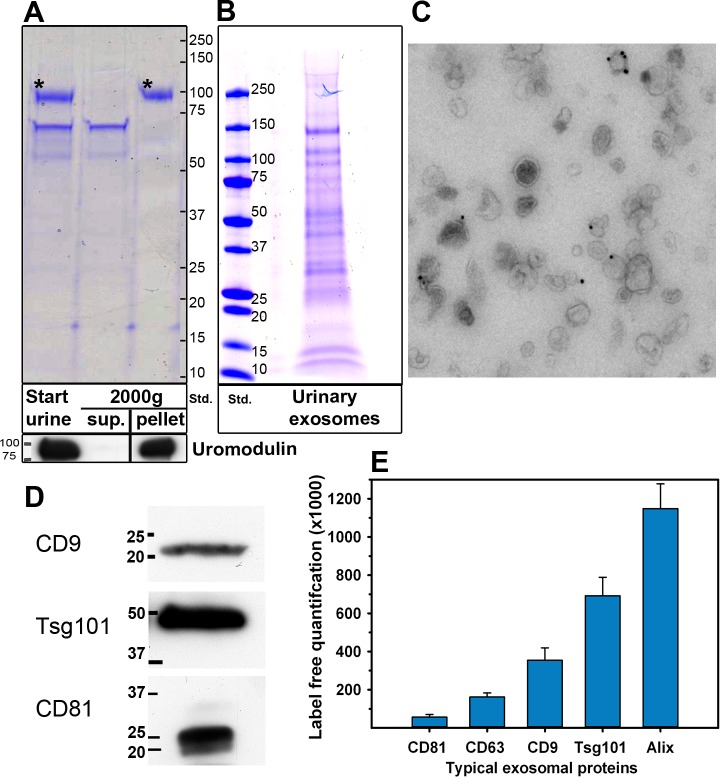
Characterization of urinary exosomes **A.** Removal of uromodulin after 2000 x g centrifugation. Urine samples (30 μl in sample buffer 4x) before (start urine) and after 2000 x g centrifugation (2000g, sup.) were loaded onto the gel. The pellet obtained after 2000 x g centrifugation was solubilized in a similar volume of water as the volume of the supernatant, and 30 μl in sample buffer 4x were loaded onto the gel (2000g, pellet). Asterisks in Coomassie stained gels (upper part) indicate uromodulin as confirmed by Western blot (lower part). Sup: Supernatant. Std: molecular weight (kD) standard. **B.** Urinary exosomes were isolated by ultracentrifugation and 10 μg were run in a 4-20% SDS-PAGE. Exosomal proteins were Coomassie stained. Std: molecular weight (kD) standard. **C.** Urinary exosomes labeled with mouse anti-CD63 followed by rabbit-anti-mouse, and then by 10 nm Protein A-gold conjugates were inspected by electron microscopy. **D.** Identification of CD9, CD81 and Tsg101 in urinary exosomes (2 μg) by Western blot. **E**. Five exosome markers detected by MS, quantified by Top 3 TIC (*n* = 15; ± SEM).

The urine samples were first centrifuged 15 min at 2,000 x g. This step removes cells and, to a large extent, uromodulin (Figure 2A). The supernatant was then centrifuged for 30 min at 10,000 x g, and finally exosomes were pelleted at 100,000 x g. The samples were maintained at room temperature until this step. The exosome pellet was washed twice with cold PBS and, before the last centrifugation, the samples were filtrated through a 200 nm filter in order to remove vesicles larger than 200 nm in diameter. As shown in Figure 2B, several bands were observed when urinary exosomes were run on a SDS-PAGE and stained. The protein pattern of urinary exosomes was completely different from crude urine (Figure 2A). This method yields 0.20 ± 0.11 (*n* = 15) μg exosomal protein/ml urine, although there are variations. The variation does not seem to correlate to the creatinine levels to a large extent (data not shown), and may be due to additional reasons such as inter-individual variations.

### Characterization of urinary exosomes

Urinary exosomes were subjected to several control analyses. As shown in Figure 2C, negative stained urinary exosomes presented the typical cup-shape morphology in electron micrographs, and no membrane fragments were observed. In addition, the exosomal marker CD63 was present in urinary exosomes (Figure 2C). The size of the exosomes, as measured by dynamic light scattering, was 149 ± 20 nm, (*n* = 15) and the size of urinary exosomes from control and prostate cancer patients was similar. As shown in Figure 2D, Western blot analysis divulged several typical exosomal markers such as CD9, CD63 and Tsg101 were found in urinary exosomes. In agreement with Figure 2D, several exosomal markers were detected in urinary exosomes by MS (Figure 2E). These experiments indicate that the vesicles isolated from urine are relatively homogeneous and not contaminated with membrane fragments, and that they contain exosomal markers.

### Mass spectrometry analysis of urinary exosomes

A preliminary MS analysis was performed to evaluate the necessary amount of sample to obtain reliable and reproducible results. Analysis of a dilution series of control urinary exosomes showed that 400 ng of exosomal protein (as measured by the BCA assay) gave reproducible results (see Supplemental Figure S1 for details), and therefore samples containing 500 ng of protein were injected in subsequent MS analyses. First, similar exosomal protein amounts (2 μg) from 6 healthy controls and 6 prostate cancer patients were submitted to tryptic digestion. The resulting peptides were analyzed by LC-MS-based bottom-up proteomics in triplicates (6 patients and 6 control samples) or single runs (remaining 9 controls and 10 patient samples). In total 1949 proteins were detected, of which 1644 were confidently identified at a protein false-discovery rate (FDR) of 1.0 % (peptide FDR 1.0%). No decoy hits were reported at this threshold. Proteins detected in single runs were only included if also detected in triplicate runs. The identified peptides matched on average 18.0 % of all identified proteins (average sequence coverage), or when taking size distribution into account, 11%/kDa. This suggests that the majority of the proteins were intact proteins rather than processed or secreted fragment peptides.

To validate the quantitative results found in the initial independent proteomic analysis of 15 control samples and 16 patient samples, a quantitative procedure was performed with iBAQ [28], where each sample type; control and patient, where pooled together and divided into three runs and normalized to total protein amount detected. This analysis gives reliable protein abundance for all candidate proteins. The linearity of the iBAQ MS-quantification was demonstrated as shown in Supplemental Figure S2.

The triplicate analysis revealed 90 % overlap from patient to control samples with a Pearson correlation of 0.87. On average, patient urinary exosomes contained 1150 proteins, while 1087 were the average number of proteins detected in exosomes from healthy male controls. 623 (36.8%) proteins were found in all of the 31 samples. Crucially, even though there is an age gap between the patient and the control group (average 63.7 ± 5.5 years versus 46.6 ± 7.3 years), no significant impact of age on the distribution of proteins was found (2-sided ANOVA, F = 0.254). We observed a tendency towards higher protein content in exosomes from patients compared to controls, but the difference in the number of proteins found in each group was not significant, even when weighted for age (Supplemental Figure S3). As urine samples from controls were first urine voids and urine samples from patients were second or third morning voids, a control experiment was performed to compare urinary exosomes isolated from first versus second voids in three control males. This study (with overlap of technical triplicates of 97 %) revealed the presence of 1350 proteins (Supplemental Figure S4). A subset of 50 proteins was significantly (*p* < 0.05) altered in first versus second morning urine exosomes (data not shown). Based on this information, we decided that if any of these proteins was found to be among the proteins significantly altered in urinary exosomes from healthy controls versus prostate cancer patients, that protein should not be considered as a potential prostate cancer biomarker (see below).

**Figure 3:**
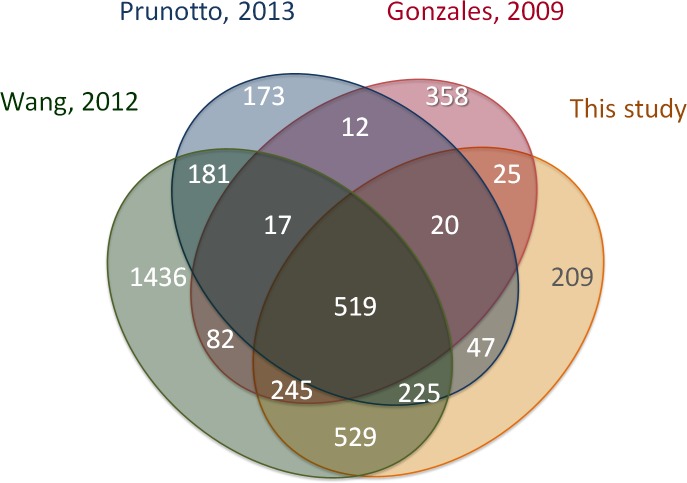
Overlap of this study with other studies of normal urinary exosomes Number of overlapping *Uniprot* entries shown in each segment based on data available from EVpedia 2.0. i) 3270 proteins from Wang 2012, ii) 1230 proteins from Prunetto 2013, iii) 1314 proteins from Gonzales 2009, iv) 1644 proteins from this study.

Since other data sets of urinary exosomes are available [24, 29-31] we performed a comparative analysis with our urinary exosomes (1644 entries found) (Figure 3). Just 32 % (519/1644) of the annotated proteins were found in all four studies investigated. Highest overlap was seen with a MudPIT analysis of urine from 9 healthy persons (89% - 1518/1644), but this study in itself shows wide distribution with an internal overlap of 31 % [30]. The low overlap with some studies may be attributed to differences in the experimental protocols used for exosomal isolation. Also, MS-based proteomics has an inherent drawback of only 75-80% reproducibility due to electron-disparity, stoichiometric preferences, and exclusion protocols used for data-dependent acquisition [32], thus showing that the method and instrument used may impact the results.

### Comparison of the proteome of control and prostate cancer urinary exosomes

**Figure 4:**
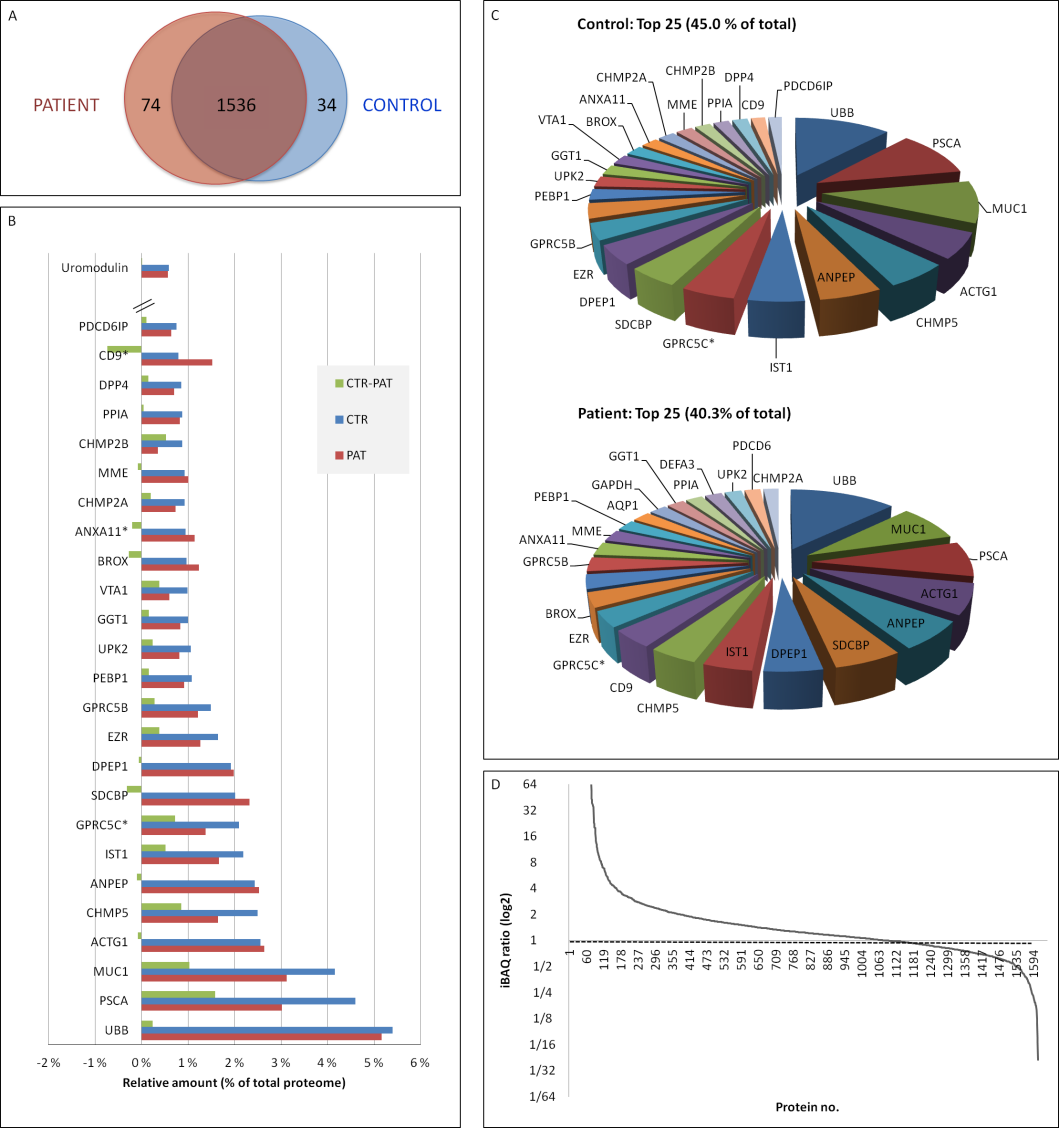
Comparison of control and patient urinary exosomal proteins **A.** Venn diagram. **B.** Amounts of the 25 more abundant proteins, and uromodulin for each dataset. PAT indicates percentage of total in patient group, CTR in control group, CTR – (minus) PAT indicates the difference between CTR and PAT groups, i.e. %CTR - %PAT. **C.** Pie diagrams of the 25 more abundant proteins in control and patient samples as measured by iBAQ. **D.** Patient versus control ratio (high to low) for all proteins detected. Ratio = 1.0 marked. * = proteins significantly altered (*p* < 0.05).

The proteins found in exosomes from healthy and prostate cancer individuals had a high qualitative overlap (93 %, 1536 of 1644 proteins) (Figure 4A). Compared to serum [33] and liver [34], the urinary exosome proteome has a large difference in abundance from common to rare proteins. The top 25 most abundant proteins in urinary exosomes from healthy controls and prostate cancer patients are shown in Figure 4B-4C. These proteins encompass 40% (as measured by individual protein abundance versus total abundance of all detected proteins) in patients and 45% in controls of the whole proteome as measured by quantitative MS. Ubiquitin was shown to be the most abundant protein in this study with up to 5% of the protein composition of urinary exosomes. Of note is the presence of several well-known exosomal proteins within the top echelon (i.e. CD9, TSG101, Alix/PDCD6IP) and interestingly, CD9 was increased in prostate cancer samples (Figure 4B). It should also be mentioned that uromodulin was also found, thus it cofractionates with urinary exosomes to some extent. However, the levels of uromodulin are not high enough (33^rd^ most abundant protein, 0.57% of total proteome) to compete with less abundant proteins and limit the sensitivity of the MS analysis. It was also noted that the levels of uromodulin were similar in control and patient urinary exosomes. Importantly, among the 1644 proteins discovered, 1203 proteins had a prostate cancer versus control ratio between 0.5 and 2 (Figure 4D). The slightly skewed distribution of ratios towards patient samples could be attributed to the fact that the presence of fewer proteins makes up the majority of the total protein for control samples (relative amount of total protein covered by 25 most abundant proteins: 45% versus 40%) and/or that urinary exosomes from prostate cancer patient have a different distribution of proteins than normal cells. i.e. express more proteins of medium to low abundance, but fewer of high abundance as seen for the total proteome analysis (Figure 4B).

### Identification of exosomal proteins significantly changed in prostate cancer patients versus controls urinary exosomes

In order to identify proteins of particular interest for prostate cancer management, proteins significantly altered between prostate cancer patients and controls urinary exosomes were investigated. This resulted in a subset of 246 proteins that were found to be significantly altered (*p* < 0.05) between the two groups (listed in Supplemental Tables S2 and S3). In particular, 221 proteins were up-regulated in prostate cancer samples and 25 proteins were down-regulated. One of the up-regulated proteins, lactotransferrin, was also found to be up-regulated in exosomes isolated from second versus first morning urine, and was therefore removed from the list (see above). Importantly, well characterized prostate cancer related proteins such as PSA and FOLH1/PSMA were also readily detected at higher level in the exosomes from patient samples.

**Figure 5:**
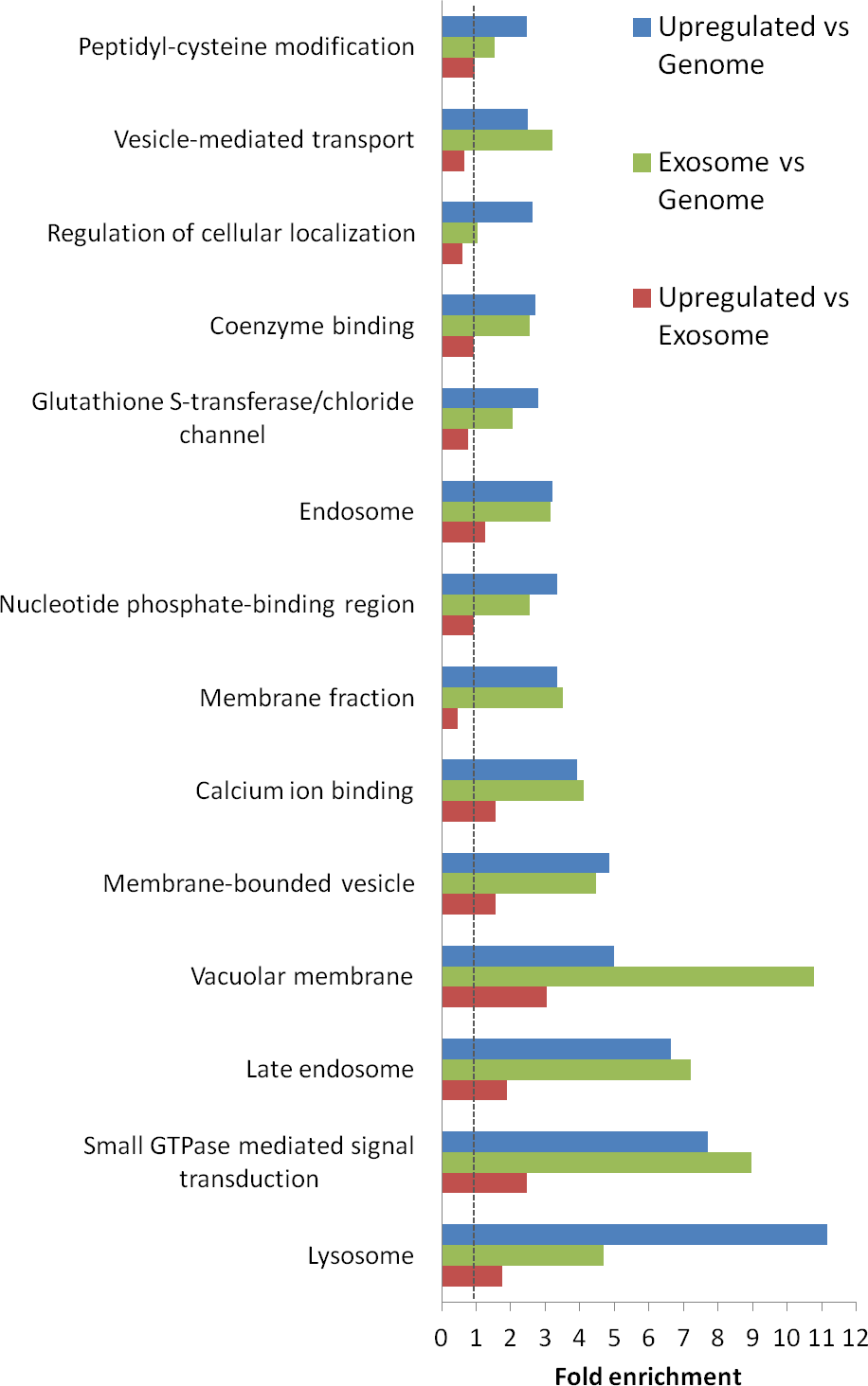
Functional analysis of proteins significantly increased in urinary exosomes from prostate cancer patients The analysis was performed using DAVID analysis tool (http://david.abcc.ncifcrf.gov/). A significant relationship was considered established at FDR < 0.5% by cluster analysis combining several annotations criteria. The red bar indicate fold-enrichment for the 221 proteins significantly up-regulated in prostate cancer urinary exosomes versus the complete exosomal proteome from this study (from the 1644 proteins, 1477 have known annotation). The green bar indicate indicate fold-enrichment for the urinary total exosomal proteome discovered in this analysis compared to the complete genome, and the blue bar indicate fold-enrichment for the 221 proteins significantly up-regulated in prostate cancer urinary exosomes versus the whole genome. No change (1.0) line marked.

An investigation of the functional categories of the proteins found to be up-regulated in the study based on Gene Ontology mapping is shown in Figure 5. The up-regulated subset of proteins/genes is compared to all the proteins/genes present in the whole genome, and to the complete set of proteins/genes found in our exosomal preparations. For reference, this urinary exosomal subproteome is also compared to the genome. Most striking is the presence of lysomal/ late endosomal proteins (11- and 7-fold increase respectively), but also the presence of small GTPase, calcium binding and GST-binding proteins is noteworthy. In addition, proteins annotated to the GO term “membrane compartments of vacuoles” are enriched compared to the genome, and also to the exosomal proteome, though to a lesser extent. However, the group of proteins associated with the overall membrane fraction is less represented among the up-regulated proteins than in the exosomal preparations. Other categories that show a decrease when comparing up-regulated proteins to the exosomal proteome are localisation and transport, indicating that these functions are less important for the disease-specific protein subset.

**Figure 6:**
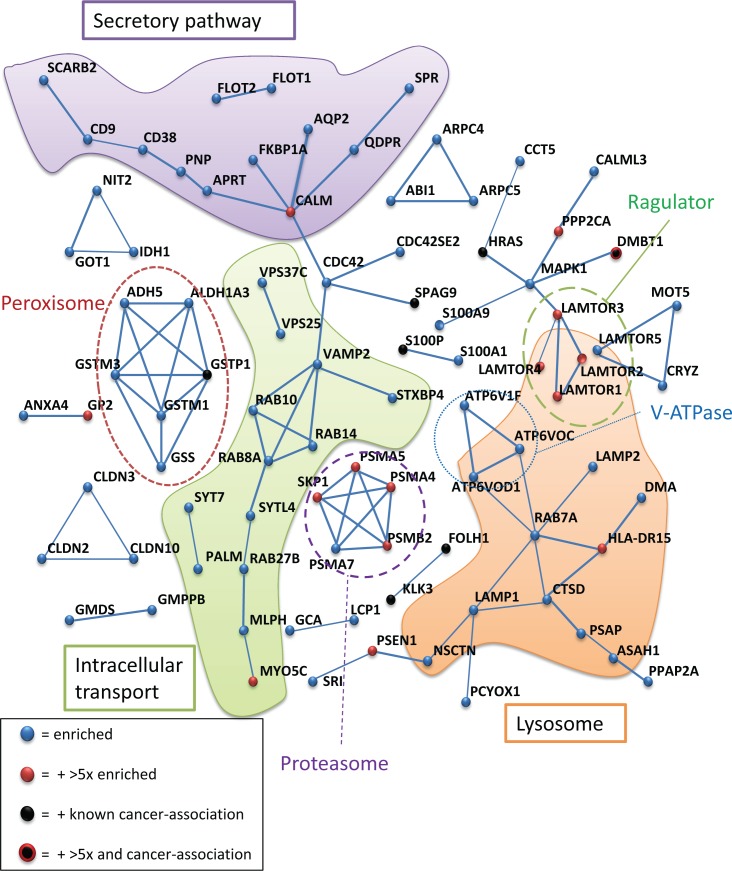
STRING protein interaction analysis of proteins significantly increased in urinary exosomes from prostate cancer patients The interaction threshold was set at 0.70 (STRING). Protein nodes with no or weak ( < 0.70) interaction are not shown. Known associations with complexes or localizations are indicated. Especially, well-known cancer-associated proteins and proteins with a higher than 5-fold enrichment, are indicated.

We found 67 % of the detected proteins to be known as exosomal proteins, and 38 % were membrane-vesicle-associated. About 25% (56/220) of the enriched proteins were related to male reproduction. Furthermore, a STRING interaction analysis of the proteins with a higher prostate cancer versus control ratio is shown in Figure 6. An emphasis was made for proteins that have a ratio higher than 5 (in red) and for proteins well-established as cancer-related (in black).

Several known protein complexes were enriched in prostate cancer patients urinary exosomes, such as the Vacuolar H+ ATPase and the LAMTOR (late endosomal/lysosomal adaptor and mitogen-activated protein kinase and mammalian target of rapamycin (mTOR) activator/regulator) complexes (average enrichment 3.7 and 4.5 respectively) (Figure 6), thus suggesting that these proteins may have a specific role in exosome release from prostate cancer cells. The exosomal (CD9- CD81-SCARB2) (average enrichment 2.40) and lysosomal (CTSD- LAMP2- CTSZ- SPNS1- PSAP) (average enrichment 4.15) proteins were also enriched in exosomes from prostate cancer patients. Finally, several components of the proteasome were also found to an increased degree in patient samples, along with the SKP1-protein. SKP1 is an essential component of the SCF (SKP1-CUL1-F-box protein) ubiquitin ligase complex (a.k.a. CRL1) that mediates the ubiquitination of proteins involved in cell cycle progression, signal transduction and transcription. Cullin-1, another component of the complex, was also detected in exosomes, but its level did not differ significantly (*p* = 0.075) between the two groups. Other proteins enriched in prostate cancer proteins such as LAPTM4A, RAB7A, AFAP, and TSPAN6 are putative targets for CRL1 as detected by screening with a NEDD8 activating enzyme inhibitor [35]. A closer look at the interactome of the proteins with a ratio patient to control above 5 shows further links between MAPKSP1/LAMTOR3, and protein phospatase 2A (PPP2CA) and ubiquitin-conjugating enzyme E2K (UBE2K) (Supplemental Figure S5A), thus connecting the LAMTOR/Ragulator complex and the proteasomal complexes. A similar map for the interactions of the 25 down-regulated proteins in urinary exosomes from prostate cancer patients could indicate a central role of the tyrosine kinase Lck, phosphatidylinositol-specific phospholipase C and ESCRT-0 proteins STAM1/2 for signalling events abolished in exosomes from prostatic cancer tissue (Supplemental Fig S5B). A significant feature of the proteins found in this subset was the presence of SH2/3-domain in 5 out of 25 proteins. Also, several of these proteins have been shown to be involved in immune response and antigen presentation (i.e. CD59, ITIH4, MARCKS, B2M, and LCK).

### Specificity and sensitivity of potential prostate cancer biomarker candidates in urinary exosomes

To evaluate the proteins significantly altered in prostate cancer patients versus controls exosomes as potential biomarkers, the specificity and sensitivity of the proteins was evaluated. A detection threshold for each protein was determined based on 100% specificity for patient samples, i.e. negative for every control sample at that threshold. The resulting sensitivity for each protein was then calculated and is shown in Table S3. The table, sorted from high to low sensitivity, also includes the quantified amount of each protein in relation to the measured proteome (as ppm).

**Figure 7:**
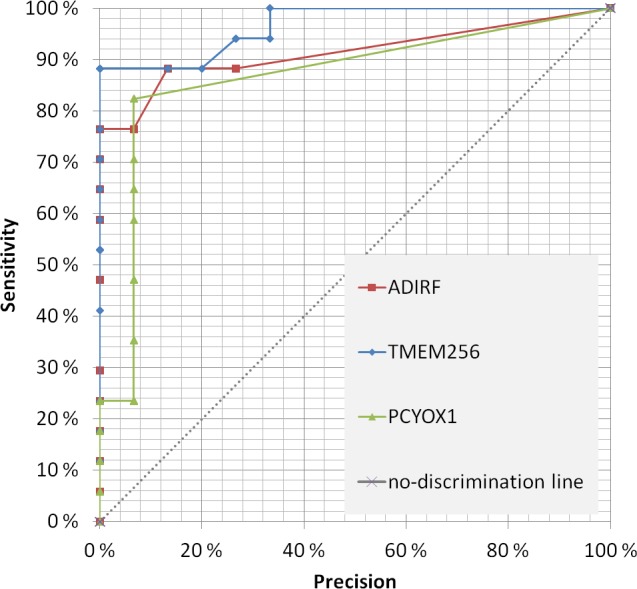
Receiver operating characteristic (ROC) for the three most promising prostate cancer biomarkers candidates in urinary exosomes Precision (calculated as the opposite of specificity) was decided for every data point and the resulting sensitivity was calculated accordingly. The area-under-curve (AUC) was determined for TMEM256 and a combination of PCYOX1 and TMEM256.

The first 37 candidates (listed in Table 1) were of particular interest since they passed the following three criteria: [i] significantly changed both by LFQ and iBAQ analysis, [ii] sensitivity above 50% at 100% specificity, and [iii] increased/decreased more than 1.75 times in patient versus control samples. We also analyzed every protein significantly enriched in prostate cancer urinary exosomes at an optimized detection threshold where its specificity and sensitivity was maximized based on the Youden's J analysis. This approach may be better for proteins that are not detected in all or most patient samples, but still are present in many patient samples and significantly enriched amounts in the quantification validation analysis by iBAQ. A heat-map for both selection approaches is shown in Supplemental Tables S4 and S5. A receiver operating characteristic (ROC) chart of the 3 most promising candidate proteins is shown in Figure 7, where area-under-curve (AUC) for TM256 as biomarker is 0.87. By combining TM256 and LAMTOR1 gives an AUC = 0.94, proving the further advantage for multiplexing biomarkers. It should be mentioned that the proteins found in the focus list were not found to be significantly different in urinary exosomes from healthy females versus males (unpublished observations).

**Table 1:** Proteins of particular interest as prostate cancer biomarkers

			Data from Discovery Analysis				Data from Quantatitive Validation Analysis	
#	Uniprot Accession Number	Protein name	#Present in CTR (of 15)	#Present in PAT (of 16)	Sensitivity at 100% specificity	#PAT at 100% specificity	Validated iBAQ ratio PAT:CTR	Abundance (ppm of total proteome)
**1**	Q8N2U0	Transmembrane protein 256	5	16	94%	15	**140.39**	4324
**2**	Q15847	Adipogenesis regulatory factor	4	15	81%	13	**18.99**	369
**3**	Q6IAA8	Ragulator complex protein LAMTOR1	4	16	81%	13	**22.98**	201
**4**	P13796	Plastin-2	14	16	75%	12	**3.15**	256
**5**	P61019	Ras-related protein Rab-2A	14	16	75%	12	**3.55**	1083
**6**	P20337	Ras-related protein Rab-3B	15	16	75%	12	**2.69**	1138
**7**	O95716	Ras-related protein Rab-3D	15	16	75%	12	**2.24**	2340
**8**	P51149	Ras-related protein Rab-7a	15	16	75%	12	**3.26**	2317
**9**	P27449	V-type proton ATPase 16 kDa proteolipid subunit	0	12	75%	12	**3.55**	861
**10**	Q687X5	Metalloreductase STEAP4	14	16	69%	11	**2.97**	953
**11**	Q99497	Protein DJ-1	15	16	69%	11	**1.92**	957
**12**	P25815	Protein S100-P	14	15	69%	11	**1.84**	1351
**13**	Q96C24	Synaptotagmin-like protein 4	5	12	69%	11	**3.08**	91
**14**	Q9NVJ2	ADP-ribosylation factor-like protein 8B	13	15	63%	10	**2.79**	49
**15**	Q96QE2	Proton myo-inositol cotransporter	2	11	63%	10	**2.66**	100
**16**	P20340	Ras-related protein Rab-6A	10	16	63%	10	**3.36**	240
**17**	O43657	Tetraspanin-6	9	16	63%	10	**4.03**	3067
**18**	P78369	Claudin-10	7	14	56%	9	**2.14**	26
**19**	P57739	Claudin-2	2	12	56%	9	**3.00**	69
**20**	O15551	Claudin-3	1	10	56%	9	**1.75**	170
**21**	O60547	GDP-mannose 4.6 dehydratase	2	12	56%	9	**2.45**	16
**22**	P46926	Glucosamine-6-phosphate isomerase 1	4	13	56%	9	**15.51**	44
**23**	Q14108	Lysosome membrane protein 2	15	16	56%	9	**3.94**	824
**24**	Q6NUT3	Major facilitator superfamily domain-containing protein 12	5	15	56%	9	**8.07**	65
**25**	Q9BV36	Melanophilin	12	16	56%	9	**2.26**	151
**26**	P35270	Sepiapterin reductase	10	14	56%	9	**2.16**	114
**27**	Q9BRA2	Thioredoxin domain-containing protein 17	15	16	56%	9	**2.35**	288
**28**	Q9BUT1	3-hydroxybutyrate dehydrogenase type 2	14	16	50%	8	**2.26**	389
**29**	P62158	Calmodulin	15	16	50%	8	**6.30**	4764
**30**	Q9Y646	Carboxypeptidase Q	1	8	50%	8	**5.80**	51
**31**	Q14254	Flotillin-2	14	16	50%	8	**2.89**	541
**32**	Q08380	Galectin-3-binding protein	15	16	50%	8	**1.99**	678
**33**	Q99571	P2X purinoceptor 4	6	13	50%	8	**2.36**	76
**34**	Q9Y3R5	Protein dopey-2	12	16	50%	8	**2.99**	218
**35**	P06703	Protein S100-A6	15	16	50%	8	**0.48**	1853
**36**	Q15286	Ras-related protein Rab-35	15	16	50%	8	**0.57**	245
**37**	P67775	Serine/threonine-protein phosphatase 2A catalytic subunit alpha isoform	11	14	50%	8	**16.91**	41

**List of the 37 significantly enriched proteins with 50% or higher sensitivity at 100% specificity, and PAT:CTR above 1.75.** The table includes the number of samples, both PAT and CTR, where the protein was detected, sensitivity at 100% specificity, number of patient samples at 100% specificity, ratio PAT:CTR based on the iBAQ quantitative analysis of pooled samples in triplicate, and relative abundance detected in the same analysis (ppm of total proteome). Yellow colour show down-regulated proteins as measured by quantitative analysis, blue colour show proteins only present in patient urinary exosomes in discovery analysis. PAT = patients samples, CTR = healthy control samples.

Several exosomal biomarkers for prostate cancer based on expressed prostatic secretions (EPS) urine have been reported [36, 37]. Importantly, many of these markers were also detected in this study, thus showing that it is not always required to perform a prostatic massage to detect prostate cancer biomarkers in urinary exosome. As a further comparison, we investigated whether a subset of urinary exosomal biomarkers that were discussed in a recent review were present in our study [12]. As shown in Supplemental Figure S6, 17 of the 20 markers presented in the review were found to be altered between the two control and the prostate cancer group in our analysis, but only 5 of them were significantly changed (*p* < 0.05).

Finally, we compared our findings in urinary exosomes with a data base compiling many published studies on prostate cancer (cancerproteomics.uio.no). Interestingly, approximately 30% of the exosomal proteins significantly changed in urinary exosomes from prostate cancer patients have been shown to be altered in other studies (Supplemental Figure S7). Concordantly, we have found 171 proteins that have not previously been found as potential biomarkers for prostate cancer. In summary, the proteins found in this study give an excellent fundament for a better diagnosis and/or prognosis of prostate cancer based on the analysis of urinary exosomes.

## DISCUSSION

This study shows that 246 proteins were significantly altered in urinary exosomes of prostate cancer patients compared to healthy controls. Specific criteria were applied to find the best potential prostate cancer biomarkers among these proteins. Importantly, among the 37 proteins selected, 17 of them displayed individual sensitivities above 60% at 100% specificity. These results, based on the proteomic analysis of more than 30 individual samples, are in agreement with the idea that exosomes are a source of biomarkers for several pathologies and may be used in clinical settings. They also confirm the power of MS-based proteome analysis in biomarker discovery.

The most interesting potential prostate cancer biomarker identified in our study (both at 100% specificity and combined specificity and sensitivity) is TM256/C17orf61, a protein that has been predicted to be located at the plasma membrane [38] and in exosomes [29]. TM256 showed the highest sensitivity (94%) and level of enrichment (140-fold) of all the detected proteins. The fact that TM256 is also relatively abundant in patient exosomes further increases the interest for this protein as a promising biomarker. Scarce knowledge exists about TM256, but it has been shown in leukaemia cells that a TM256-fusion protein with non-receptor tyrosine kinase TNK1 (TNK:C17orf61) leads to a constitutively active TNK1 that is associated with uncontrolled growth [39]. Other promising prostate cancer biomarkers are LAMTOR1 and ADIRF (81% sensitivity at 100% specificity). Moreover, LAMTOR1 could be used to augment the sensitivity to 100% in combination with TM256 (Figure 7). It is important to note that well known prostate cancer biomarkers including PSA, FOLH1/PMSA, TGM4, and TMPRSS were also found to be enriched in urinary exosomes from prostate cancer patients compared to controls. However, compared to some of the novel candidates described here, these proteins showed lower degree of specificity and/or sensitivity. In our opinion, the presence of known prostate cancer markers in urinary exosomes gives further credibility to the novel proteins identified in our study

This is as far as we know the first time that the proteome of exosomes isolated from non manipulated urine samples from prostate cancer patients have been characterized to this degree of detail. Previous studies have mainly used exosomes isolated from urine collected after prostatic massage in order to increase the amount of prostatic exosomes in the urine sample [37, 40]. Interestingly, we observed that almost all of the exosomal proteins described as significantly enriched in prostate cancer in a recent review based on urine collected after prostatic massage were also found in our study [12]. Therefore, our results suggest that it is unnecessary to perform prostatic massage prior to urine collection to detect prostate cancer biomarkers. There are to our knowledge no studies that have directly compared the levels of the specific exosomal proteins enriched in prostate cancer in both types of urine from prostate cancer patients. If the level of these proteins is higher in urine collected after prostatic massage, it can be useful to perform this procedure when low sensitive methods are used to identify these proteins.

Prostate-derived exosomes in urine probably originate from prostatic fluid that is drained when urine flows through the prostatic urethra. It is not clear to which extent the amount of prostatic fluid varies between subjects. In some of the studies presented here, we have reduced this potential variability by pooling control and patient samples in three groups. It would be useful in future experiments to measure urinary PSA in order to investigate the variability of prostatic fluid in urine samples.

In addition to the biomarker perspective, the enrichment of several classes of proteins in urinary exosomes from prostate cancer patients may provide an insight into the machinery behind the release of exosomes during disease progression. Our study reveals that several of the subunits of the vacuolar proton-transporting ATPase (V-ATPase), an enzyme responsible for acidifying a variety of intracellular compartments in eukaryotic cells, are enriched in urinary exosomes. This may indicate that the regulation of the proton gradient is especially important for the release of exosomes from prostate cancer cells, although recent studies suggest that V-ATPase can affect intracellular transport by a pH-independent mechanism [41, 42]. The proteolipid subunit V0c, uniquely detected in patient samples, is the target of bafilomycin A1 and concanamycin A, drugs that affect the transport from late endosomes to lysosomes [43]. Importantly, it has been shown that inhibition of V-ATPase also affects prostate cancer invasion and PSA secretion [44, 45]. Similarly, knock-down of LASS2/TMSG1, which reduces V-ATPase activity through V0c-binding, led to increased metastasis and prostate cancer progression [46].

Closely related to V-ATPase are the components of the LAMTOR/Ragulator complex, since it has been shown that V0 is required for the regulation of mammalian target of rapamycin complex 1 (mTORC1), the hetero-oligomeric assembly of mTOR, raptor, and mLST8 [47]. Several reports have demonstrated a pivotal role of LAMTOR proteins in fundamental cellular processes such as cell proliferation, growth factor signaling, and endosomal rearrangement [48-50]. All the components of the LAMTOR complex, which consists of p18 (LAMTOR1), p14 (LAMTOR2), MP1 (LAMTOR3), C7orf59 (LAMTOR4), and HBXIP (LAMTOR5), were found enriched in patient urinary exosomes (see Table 1). In cell homeostasis, the amino acid sensing function of the LAMTOR complex recruits mTOR to the lysosome for autophagy inhibition [47, 51]. Interestingly, Rag A and Rag C, proteins that together with LAMTOR also participate in this process [52], were detected in some patient samples, but not in control samples, but the differences were not significant (data not shown). Importantly, TM256, the protein with the highest sensitivity and level of enrichment in patients urinary exosomes has recently been linked to LAMTOR4 in an affinity-MS study (http://thebiogrid.org/166968/publication) and may be another partner in the LAMTOR complex. ADP-ribosylation factor-like 8b (ARL8B), among the enriched proteins in prostate cancer patients urinary exosomes, has been associated with the LAMTOR complex too, in particular with LAMTOR2/3 and its role in cell migration [53]. Finally, Golgi phosphoprotein 3 (GOLPH3), a protein involved in Golgi-membrane integrity and mTOR amplification [54], is enriched in prostate cancer patients urinary exosomes as well. This proteins has been described as an oncogene in several cancers such as pancreatic ductal adenocarcinoma [55], glioblastoma [56], hepatocarcinoma [57], gastric cancer [58], and prostate cancer [59]. Mechanistically, GOLPH3 regulates cell size, enhances growth-factor-induced mTOR signalling in human cancer cells, and alters the response to rapamycin *in vivo* [60].

Adipogenesis regulatory factor (ADIRF), ranked among the top candidates, correlates with resistance to cisplatin in several cancer cell types [61]. Its gene, APM2, has also been found to be considerably down-regulated in androgen-ablated prostate tumour cells [62]. Its precise function remains unknown.

Lysosomal hydrolases have previously been shown to affect cancer development in different ways [63, 64]. The increased levels of several endosomal/lysosomal peptidases (e.g. cathepsin D, carboxypeptidase Q, probable serine carboxypeptidase CPVL, napsin A) in prostate cancer patients urinary exosomes could be related to cancer progression. Many Rab proteins are normally found in exosomes and several Rab proteins have been shown to regulate exosome release e.g. Rab27 [65] and Rab35 [66]. Our study divulged as many as 18 different Rab proteins significantly enriched in patients urinary exosomes. In particular, Rab2A, Rab6A and Rab7a were more than 3-fold enriched, while Rab35 was the only Rab protein that was down-regulated in patients derived exosomes. Rab27 has been shown to control vesicle release and deliver critical proinvasive growth regulators into the tumor microenvironment. Rab27B regulates invasive growth and metastasis in estrogen-receptor-positive breast cancer cell lines, and increased expression is associated with poor prognosis [67]. Tetraspanins are also common exosomal proteins. Several tetraspanins such as CD81, CD9, tetraspanin-6 and tetraspanin-8 were enriched in prostate cancer versus control samples. This may indicate a specific function of these proteins in exosome release from prostate cancer cells. Moreover, the levels of tetraspanin-8, have been shown to be increased in cells with improved metastatic ability [68], and has been implied in invasiveness in several cancer types [69-72].

The methodology used to isolate exosomes is still the subject of current discussions. In addition, the type of biofluid also plays a role when choosing an isolation method. Urinary exosomes have mainly been isolated by ultracentrifugation [21, 29], but there is no consensus about a specific protocol. One of the challenges in the isolation of urinary exosomes is to remove uromodulin, also called Tamm-Horsfall protein, the most abundant protein in urine. This protein forms high-molecular-weight filaments that can entrap exosomes and negatively affect their recovery [73]. Several methods have been envisaged to avoid this problem, for example, to use a reducing agent to depolymerize uromodulin or to float exosomes on a sucrose gradient [21, 29], but these methods also have some limitations (low yield, potential alteration of exosomal proteins). In this study, we have optimized an ultracentrifugation based method that considerable avoids the co-isolation of uromodulin with exosomes. Uromodulin is pelleted at low-speed centrifugation at room temperature, and our results show that that the uromodulin that remains in the supernatant does not pellet to a large extent when higher centrifugation speeds are performed at room temperature. Moreover, a challenge of urinary proteomics is the variation of protein concentration in urine samples because of fluid intake and renal function. However, it is not clear if this affects the release of exosomes, and therefore we decided not to normalize the exosome yield by urine creatinine. Finally, we observed that there were minimal differences in the exosome proteome of first and second morning voids.

As expected, a comparison against our previous proteomic studies of exosomes derived from the prostate cancer cell line PC-3 revealed differences as well as similarities [13]. The differences are in part due to the the mass spectrometric capacity and sensitivity of the instruments used, but they probably also reflect the differential expression of proteins in *in vivo* and *in vitro* systems, emphasizing the importance of *in vivo* studies for biomarker analysis. An example is the nearly complete absence of integrins in urinary exosomes compared to PC-3 exosomes, were several integrins were among the most abundant proteins detected [13].

The results presented here need further validation in independent cohorts in order to evaluate if they can be used in the clinic. Furthermore, validation of these results using alternative methodologies is also an important issue since mass spectrometry analyses are not widely used in clinical settings. Antibody-based methods, and in particular Elisa tests, are commonly used in clinical laboratories, and we will evaluate the possibility to use antibody-based methods in such a validation study. However, some of the challenges of using antibodies are that antibodies are often not as specific as claimed by the manufacturer, and that they may not be sensitive enough to detect changes in protein levels that are detectable by MS.

The use of urinary exosomal proteins as prostate cancer biomarkers is at its infancy. However, one of the advantages of urinary biomarkers versus prostate tissue biomarkers is that, due to the heterogeneity of the prostate tissue, urinary exosomes provide a global picture of the tissue compared to prostate biopsies. It should also be mentioned that, due to the complexity of prostate cancer, it does not seem very likely to find a single molecule for the diagnosis/prognosis of the disease, and probably a panel of biomarkers will be required. We have observed that combination of some of the molecules described in this study show increased performance compared to single proteins. To ensure the quality of potential biomarkers discovered in this study from 16 patients a few days before prostatectomy a closer investigation of the distribution of these protein candidates in an additional number of samples is required. Given the high specificity and selectivity of several of the candidates, chances for a positive outcome of an evaluation in an independent validation are high.

## MATERIALS AND METHODS

### Materials

Bovine serum albumin (BSA) was purchased from Sigma-Aldrich (St. Louis, MO, USA). Bicinchoninic acid (BCA) protein assay kit and Imperial Protein stain was from Pierce (Thermo Scientific, Rockford, IL, USA). Mini-protean TGX 4-20% polyacrylamide gels and Transfer-Blot Turbo Transfer Pack were from Bio-Rad (Hercules, CA, USA). PVDF membranes were from Millipore (Billerica, MA, USA). The antibodies used for Western blotting were: mouse anti-Tsg101 (BD Biosciences, Heidelberg, Germany); rabbit anti-CD9 (Abcam, Cambridge, UK); mouse anti-CD81 (Ancell Corporation, Bayport, MN, USA), rabbit anti-uromodulin (St. Cruz Biotechnology Inc., Dallas, TX, USA). HRP-conjugated secondary antibodies were from Jackson Immunoresearch (West Grove, PA, USA). The antibodies used for immuno-electron microscopy were: mouse anti-CD63 (H5C6) (DSHB, Iowa city, IA, USA) and rabbit-anti-mouse (DACO, Glostrup, Denmark). Protein A-gold conjugates (10 nm) were purchased from Cell Microscopy Center (Utrecht, Netherlands).

### Urine samples

Urine samples were collected either from 15 healthy volunteers (without any diagnosed condition and full-time employees) or from prostate cancer patients (17 samples, but a sample was excluded from the proteomic analysis due to low exosomal protein yield) before robot-assisted laparoscopic radical prostatectomy. The cohort description is presented in Supplemental Table S1. For practical reasons, urine samples from prostate cancer patients were collected during the morning, and for control samples the first void of the day was collected. Therefore, as a control, exosomes from first and second voids of the day were collected and compared. The urine pH and the presence of leukocytes, nitrites, proteins, glucose, ketones and blood was analyzed with a Combur^7^ strip-Test in an Urysis 1100 urine analyzer (Roche Diagnostics, Basel, Switzerland). he level of leukocytes (Leu) is classified as 25 Leu/μl: + (low; Table S1), 100 Leu/μl: ++, 500 Leu/μl: +++. The level of erythrocytes (Ery) is classified as 10 Ery/μl: + (low; Table S1), 25 Ery/μl:++, 50 Ery/μl: +++, 250 Ery/μl: ++++. The level of glucose (Glu) is classified as 2.8 mM: + (low; Table S1), 5.5 mM: ++, 17 mM: +++, 56 mM: ++++. Creatinine was measured with a creatinine urinary detection kit (Arbor Assays, Ann Arbor, MI, USA). The collection of urine samples was approved by the Norwegian Regional Committees for medical and health research ethics.

### Exosome isolation

Urinary exosomes were isolated by serial centrifugation. Briefly, urine (in general 50-150 ml) was centrifuged at 2,000 x g for 15 min at room temperature (RT), and then at 10,000 x g for 30 min at RT discarding the pellet at each step. Then, the exosomes present in the supernatant were pelleted at 100,000 x g for 70 min at RT in a Ti70 rotor, washed with PBS, and centrifuged at 100,000 x g for 70 min at 4°C in a Ti70 rotor. Exosomes were then resuspended in PBS, vortexed, filtrated through a 200 nm pore Supor syringe filter (Pall corporation, Port Washington, NY, USA), and finally pelleted at 100,000 x g for 70 min at 4°C in a SW40 rotor. The supernatant was removed leaving 50-100 μl in the bottom to resuspend the pellet. Exosomes were submitted to several analyses to control the purity and yield, and then were stored at −80°C until further use.

### Protein measurements

The amount of protein in exosomes was determined using a BCA assay kit according to the manufacturer's instructions. BSA was used as standard protein.

### Electron microscopy of exosomes

Exosomes resuspended in PBS were fixed (4% formaldehyde/0.2% glutaraldehyde) and deposited on formvar/carbon-coated copper grids. For labelling, samples on grids were first blocked with 0.5% BSA, and then successively incubated with mouse anti-CD63 followed by rabbit-anti-mouse, and then by 10 nm Protein A-gold conjugates. Fixative, blocking solution and antibody dilutions were prepared in PHEM buffer (60 mM PIPES, 25 mM HEPES, 10 mM EGTA and 2 mM MgCl_2_ at pH 6.9). Samples were then contrasted and embedded in a mixture of methylcellulose and uranylacetate. Finally, exosomes were observed in a JEOL-JEM 1230 (JEOL Ltd., Tokyo, Japan) at 80 kV and pictures were acquired using a Morada camera and iTEM software (Olympus, Münster, Germany).

### Size determination of exosomes

The size of exosomes was determined by dynamic light scattering performed with a Zetasizer Nano ZS (Malvern Instruments Ltd, Worcestershire, UK). Isolated exosomes were diluted in PBS and the size determination was performed at 25°C according to the manufacturer's instructions. The size parameter is based on the intensity of the scattering particles and presented, z-average.

### SDS-PAGE and staining

Samples were mixed with loading buffer and run on 4-20% polyacrylamide gels. The gels were stained using a Ready-to-use Coomassie stain following the manufacturer's protocol.

### Immunoblotting

After separation on SDS-PAGE the proteins were transferred to PVDF membranes using a Transfer-Blot Turbo Transfer Pack for immunoblotting. Membranes were incubated with the specified primary and secondary antibodies, and finally blots were visualized with the AmershamTM ECLTM Prime Western blot detection (GE Healthcare, Little Chalfont, UK) on the Universal Hood II Bio-Rad scanner (Bio-Rad, Hercules, CA, USA).

### In-solution digestion of exosomes

Exosomes (2 μg) in one volume of PBS were mixed with four volumes of cold acetone (with 1M HCl) and methanol at −20°C. The samples were centrifuged at 15,000 x g for 15 min and the pellets were dried in a Speed-Vac. Then, the pellets were dissolved in 50 μl of a fresh solution of 100 mM ammonium bicarbonate with 6 M urea, and subsequently reduced with 10 mM dithiothreitol at 30°C for 30 min. The samples were then incubated with 25 mM iodoacetamide to alkylate exposed side chains for 1 h at room temperature protected from light. The enzymatic digestion was initiated by adding 1 μg Lys-C to the samples and incubating them at 37°C for 2 hours. Finally, 240 μl 50 mM ammonium bicarbonate with 10 μg of trypsin was added and the samples were first incubated for 1 h at 37°C, followed by 15 h at 30°C. Peptides were purified by C_18_ Zip Tips (Millipore, Billerica, MA, USA). Prior to LC-MS analysis, 5 μl formic acid was added to the digested exosomes.

### Mass spectrometric analyses

Two different mass spectrometers were used in this study. For LC-MS analyses of the complete dataset – (discovery analysis) an LTQ Orbitrap XL was used. The samples (one quarter of the volume, 0.5 μg) were injected into an Ultimate 3000 nano-UHPLC system (Dionex, Sunnyvale CA, USA) connected to a linear quadrupole ion trap-orbitrap (LTQ-Orbitrap XL) mass spectrometer (ThermoScientific, Bremen, Germany) equipped with a nanoelectrospray ion source. An Acclaim PepMap 100 column (C18, 3 μm, 100 Å) (Dionex) with a capillary of 25 cm bed length was used for separation by liquid chromatography. Solvent A was 0.1% formic acid, whereas aqueous 90% acetonitrile in 0.1% formic acid was used as solvent B. A flow rate of 300 nl/min was employed with a solvent gradient of 4% B to 60% B in 230 min.

The mass spectrometer was operated in the data-dependent mode to automatically switch between MS and MS/MS acquisition. Survey full scan MS spectra (from m/z 400 to 1,700) were acquired with the resolution R = 70,000 at m/z 200, after accumulation to a target of 3e6. The maximum allowed ion accumulation times were 100 ms. The method used allowed sequential isolation of up to the ten most intense ions, depending on signal intensity (intensity threshold 1.7e4), for fragmentation using higher collision induced dissociation (HCD) at a target value of 20,000 charges and a resolution R = 35,000 Target ions already selected for MS/MS were dynamically excluded for 60 sec. The isolation window was m/z = 2 without offset. The maximum allowed ion accumulation for the MS/MS spectrum was 120 ms. For accurate mass measurements, the lock mass option was enabled in MS mode and the polydimethylcyclosiloxane ions generated in the electrospray process from ambient air were used for internal recalibration during the analysis.

To validate the quantitative analyses for the complete data set, the samples (aliquots of the digested exosomes that were used in the previous analysis) were pooled into three sets of patient exosomes and three sets of controls (aliquots of digested exosomes and subjected to LC-MS with internal standard (iBAQ- intensity based absolute quantification)[74].

The iBAQ quantification experiments were performed on an Easy nLC1000 nano-LC system connected to a quadrupole – Orbitrap (QExactive) mass spectrometer (ThermoElectron, Bremen, Germany) equipped with a nanoelectrospray ion source (EasySpray/Thermo). For liquid chromatography separation we used an EasySpray column (C18, 2 μm beads, 100 Å, 75 μm inner diameter) (Thermo) capillary of 25 cm bed length. The flow rate used was 0.3 μL/min, and the solvent gradient was 5 % B to 30 % B in 240 minutes, then 90 % B wash in 20 minutes. Solvent A was aqueous 0.1 % formic acid, whereas solvent B was 100 % acetonitrile in 0.1 % formic acid. Column temperature was kept at 60 ^o^C.

The mass spectrometer was operated in the data-dependent mode to automatically switch between MS and MS/MS acquisition. Survey full scan MS spectra (from m/z 400 to 1,200) were acquired in the Orbitrap with resolution R = 70,000 at m/z 200 (after accumulation to a target of 3,000,000 ions in the quadruple). The method used allowed sequential isolation of the most intense multiply-charged ions, up to ten, depending on signal intensity, for fragmentation on the HCD cell using high-energy collision dissociation at a target value of 100,000 charges or maximum acquisition time of 100 ms. MS/MS scans were collected at 17,500 resolution at the Orbitrap cell. Target ions already selected for MS/MS were dynamically excluded for 30 seconds. General mass spectrometry conditions were: electrospray voltage, 2.0 kV; no sheath and auxiliary gas flow, heated capillary temperature of 250^o^C, normalized HCD collision energy 25%. Ion selection threshold was set to 5e4 counts. Isolation width of 3.0 Da was used. Proteins that were present only in 1 of the 3 sets were considered invalid.

### Data processing

Data were acquired using Xcalibur v2.5.5 and raw files were processed to generate peak list in Mascot generic format (*.mgf) using ProteoWizard release version 3.0.331. Database searches were performed using Mascot in-house version 2.4. to search from Swiss-Prot selected for homo sapiens (11.2013, 20,252 entries) assuming the digestion enzyme trypsin with at maximum one missed cleavage, fragment ion mass tolerance of 0.60 Da, and a parent ion tolerance of 10 ppm. Carbamidomethyl of cysteine was specified in Mascot as a fixed modification. Oxidation of methionine, acetylation of the N-terminus and phosphorylation of serine, threonine and tyrosine were specified in Mascot as variable modifications. Scaffold (version Scaffold_4.3.2, Proteome Software Inc., Portland, OR, USA) was used to validate MS/MS based peptide and protein identifications. Peptide identifications were accepted if they could be established at greater than 95.0% probability by the Peptide Prophet algorithm [75] with Scaffold delta-mass correction. Protein identifications were accepted if they could be established at greater than 99.0% probability and contained at least one identified peptide. Protein probabilities were assigned by the Protein Prophet algorithm [76]. Proteins that contained similar peptides and could not be differentiated based on MS/MS analysis alone were grouped to satisfy the principles of parsimony. MS/MS spectra from protein hits identified with only 1 peptide were investigated manually.

For comparing datasets Fisher's exact test (CI 95%) was used to determine significant changes between the subproteomes of exosomes from patients and healthy controls. The label-free quantitative measurement of individual samples used both peptide spectra match (PSM) and top 3 precursor intensities from total ion chromatogram (TOP3TIC) and only protein hits significantly altered (heteroscedastic two-sided *t*-test, *p* < 0.05) for both were considered. The suitability as biomarker for the candidate proteins were addressed by determining an intensity threshold in every sample. The intensity threshold was optimized to give maximum specificity and sensitivity of the test, based on Youden's J plot [77]. This enabled us to produce a heat map displaying most promising candidates within the cohort for further evaluation.

For iBAQ analysis the MS raw files were submitted to MaxQuant software version 1.4.0.8 [78] for protein identification. Parameters were set as follow: protein N-acetylation, methionine oxidation and pyrogluatamte conversion of Glu and Gln as variable modifications. First search error window of 20 ppm and mains search error of 6 ppm. Trypsin without proline restriction enzyme option was used, with two allowed miscleavages. Minimal unique peptides were set to 1, and FDR allowed was 0.01 (1%) for peptide and protein identification. The Uniprot human database was used (download from December 2013). Generation of reversed sequences was selected to assign FDR rates.

The pooled control samples triplicate were normalised to the total iBAQ-values for the pooled patient sample, and only proteins found in previous experiment investigated for significant iBAQ-change (two-sided, *t*-test, *p* < 0.05). For functional analysis DAVID version 6.7 (http://david.abcc.ncifcrf.gov/) were used [79], while STRING v9.1 (http://string-db.org/) were utilized for network analysis [80, 81]. Additional bioinformatic data was obtained from EVpedia (v2.1) (http://evpedia.info) [82], Vesiclepedia (v3.1) (microvesicles.org) [83] and Cancer Proteomics Database at University of Oslo (cancerproteomics.uio.no) (by courtesy of M. Arntzen & B. Thiede). We have deposited our data in PRIDE; depository accession number PXD090912.

## SUPPLEMENTARY MATERIAL FIGURES AND TABLES



## Abbreviations

a.k.a.: also known as
BCA: bicinchoninic acid
BSA: bovine serum albumin
CTR: control
EPS: expressed prostatic secretions
ESCRT: endosome sorting complex required for transport
EV: extracellular vesicle
FDR: false discovery rate
FOLH: folate hydrolase (a.k.a. PSMA, prostate-specific membrane antigen)
iBAQ: intensity based absolute quantification
LAMTOR: late endosomal/lysosomal adaptor and mitogen-activated protein kinase and mammalian target of rapamycin (mTOR) activator/regulator
LC/MS/MS: liquid chromatography tandem mass spectrometry
LFQ: label-free quantification
MS: Mass spectrometry
MVB: multivesicular body
PPM: parts per million
PBS: phosphate buffered saline
PSA: prostate specific antigen (a.k.a KLK3, kallikrein-3)
PSM: peptide spectra match
PTM: post-translational modifications
RT: room temperature
TOP3TIC: top 3 precursor intensities from total ion chromatogram
