# Supplementary material for: Identification of prostate cancer biomarkers in urinary exosomes
Source: Oncotarget. 2015 Jul 13;6(30):30357–76. doi: 10.18632/oncotarget.4851 (PMC4745805; doi:10.18632/oncotarget.4851)
Supplement: Supplementary file 1 [file oncotarget-06-30357-s001.pdf]

Identification of prostate cancer biomarkers in urinary exosomes

Supplementary Material

<u>Figures</u>

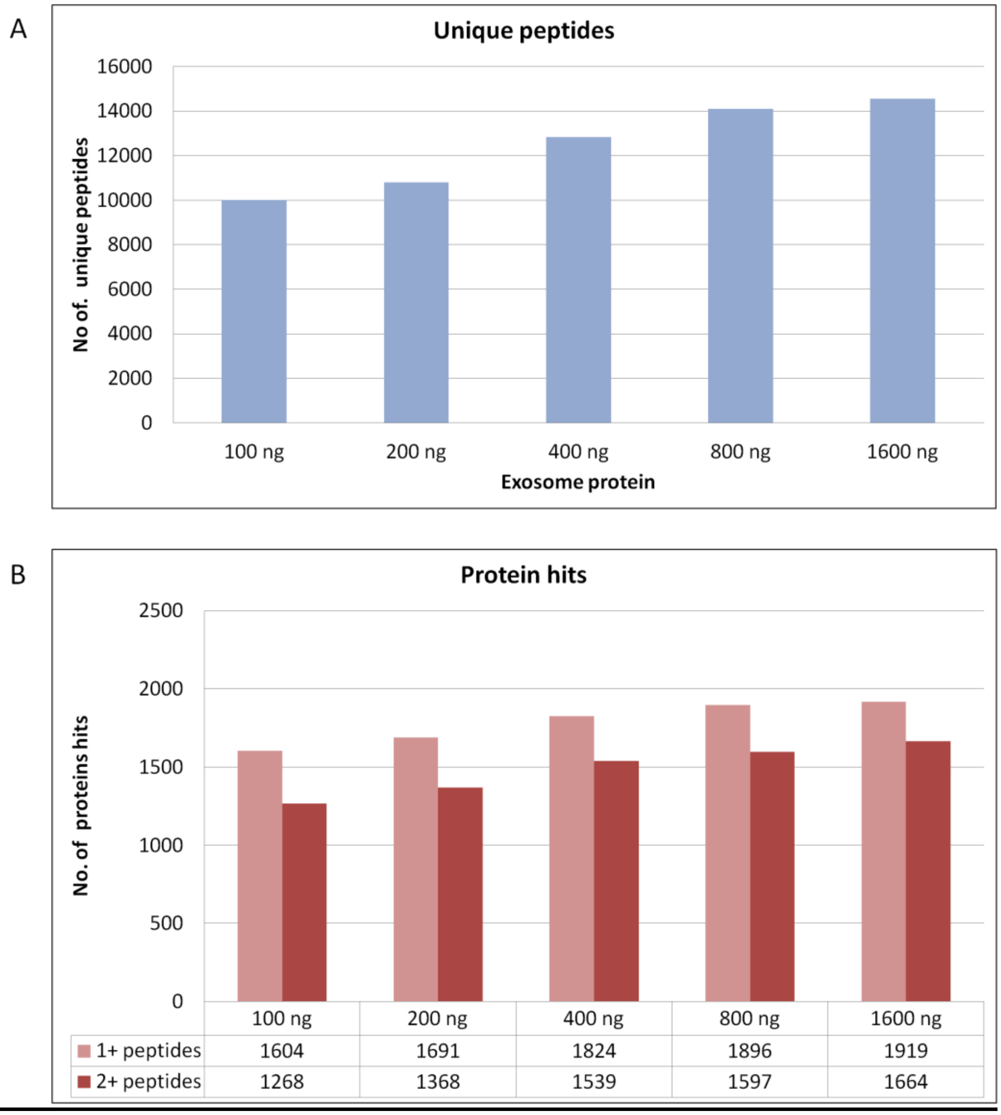

Fig. S1. Optimalization of exosomal protein amounts for mass spectrometry analysis. Increasing amounts of trypsinated exosomes were injected into a mass spectrometer to establish the minimal protein amount required for analysis. Number of unique peptides (A) and number of protein hits for 2 or more peptides, and 1 or more peptides (B) found at the corresponding exosomal protein amounts. The protein amount was measured using the BCA assay.

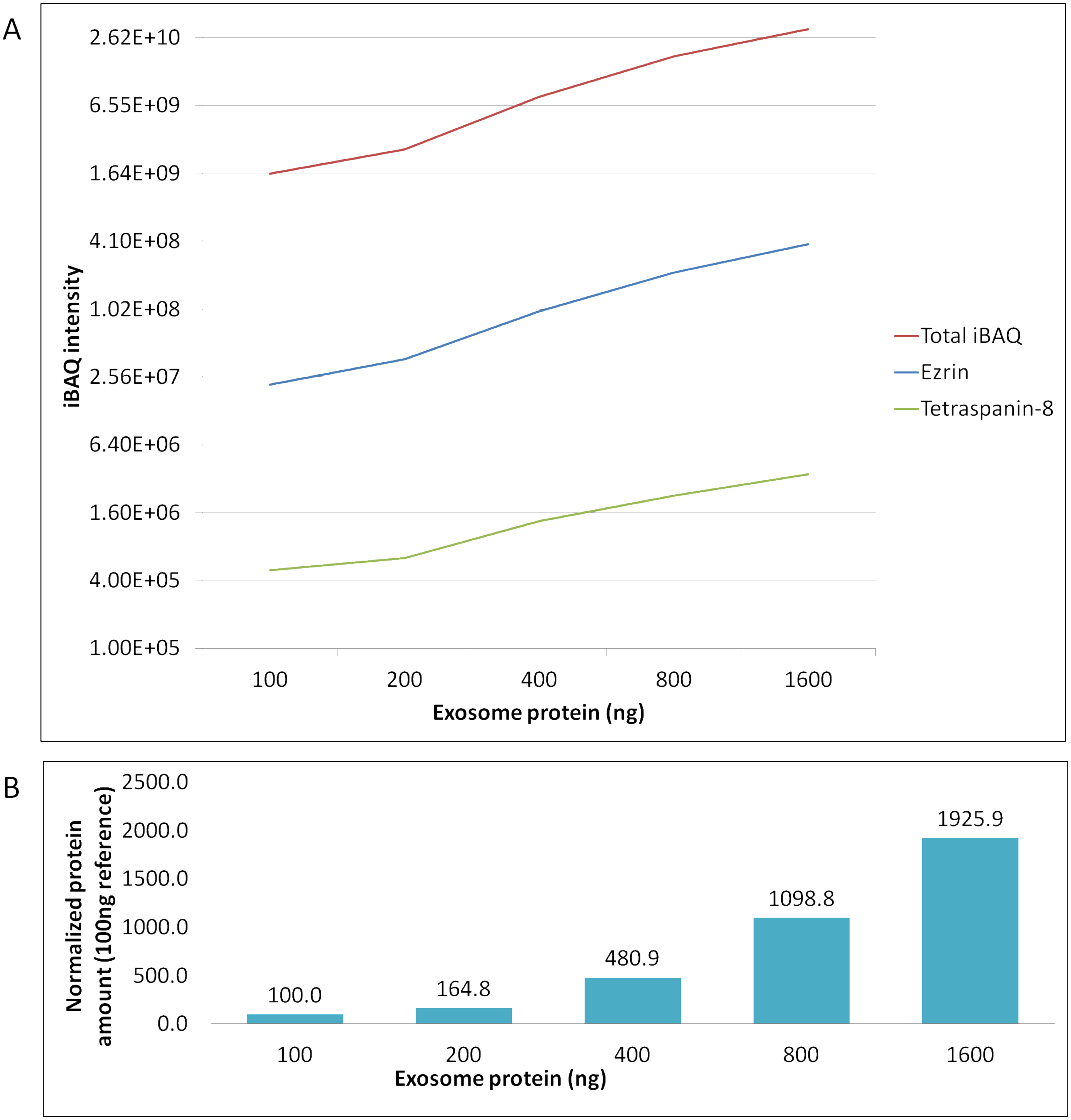

Fig. S2. Quantification analysis. Increasing amounts of trypsinized exosome proteins were injected into an LTQ-Orbitrap XL and subjeced to lock mass quantification. A. Both total and individual quantification for two proteins (ezrin and tetraspanin-8) gave a linear response within the quantification range measured (R^2= 0.987 (total), 0.9914 (ezrin), 0.9845 (tetraspanin-8). B. Quantitative values in ng (normalized to 100 ng) for total protein abundance as measured by iBAQ. iBAQ: Intensity-based absolute quantification.

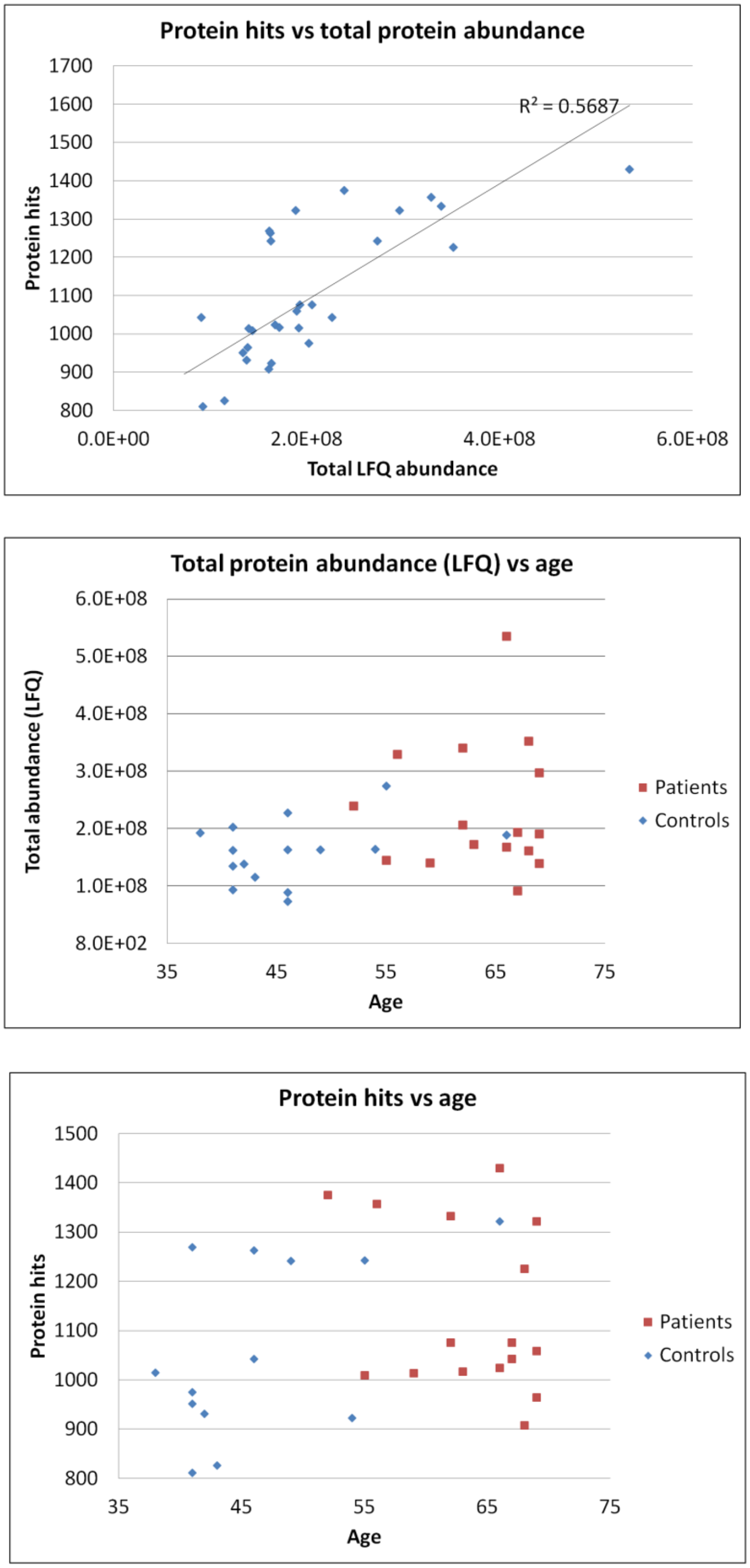

Fig. S3. Correlation studies of age, abundance and number of protein hits. Scatter plots investigating the correlation between total protein abundance (LFQ) and protein hits, age and total protein abundance (LFQ), and age and protein hits. Total protein abundance was measured as sum of TOP3TIC for all proteins identified in each sample.

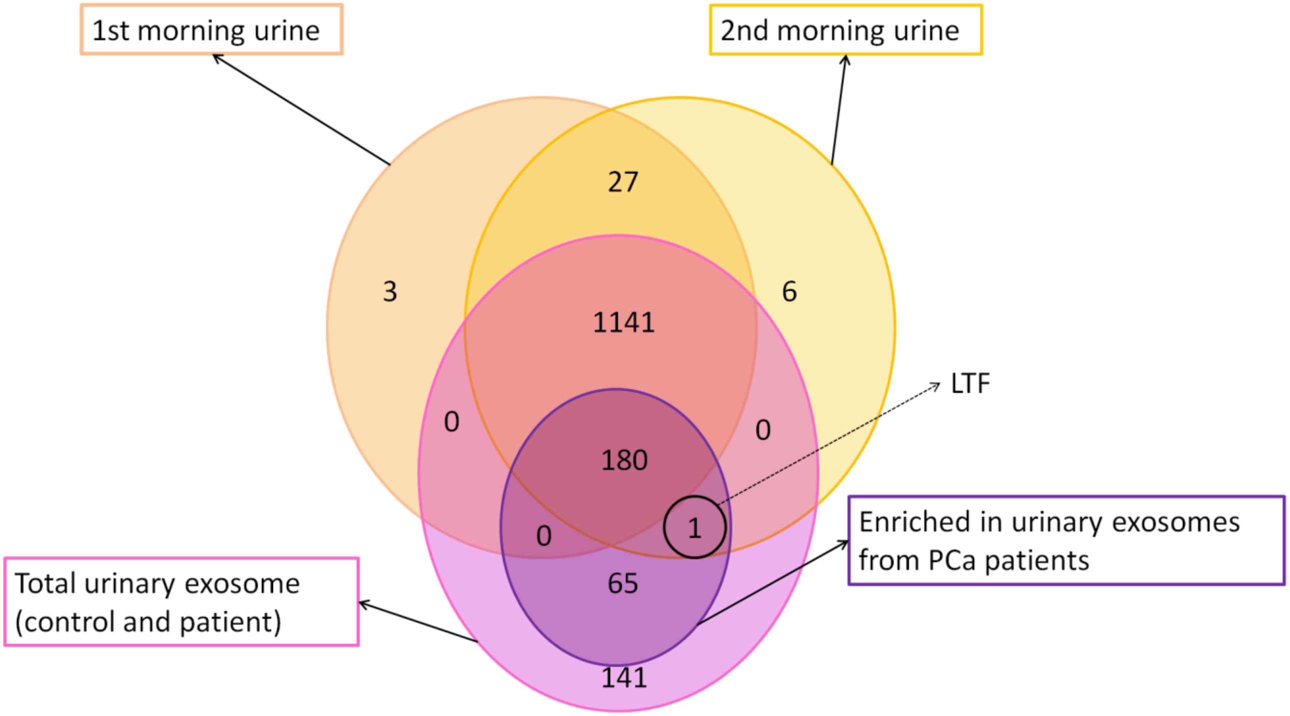

Fig. S4. Comparison of proteins found in exosomes isolated from first and second morning urine voids. The reproducibility of the three technical replicates was of 96.7%. Seven proteins were found exclusively in second urine (lactotransferrin (LTF), complement C4-B, sulfate transporter, protein AMBP, tetratricopeptide repeat protein 38, prolactin-inducible protein and fibrinogen alpha chain) and three proteins were found only in first urine (histidine triad nucleotide-binding protein 1, small proline-rich protein 2A, and serine/threonine-protein kinase PAK 2). Total urinary exosome is composed of proteins found in both control and patient protein samples.

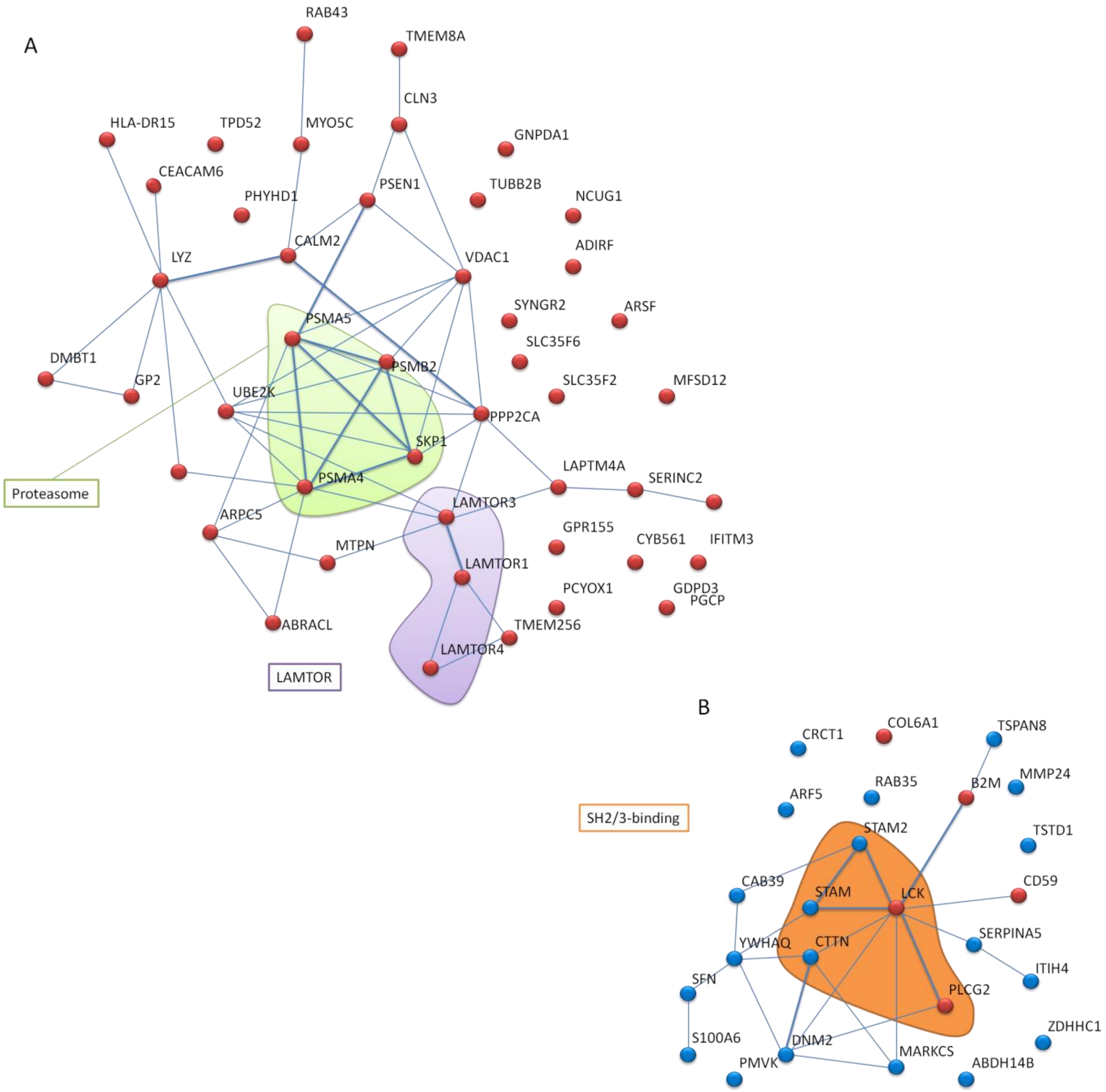

Fig. S5. STRING analysis. A. Protein interaction analysis of the 46 most up-regulated proteins (>5-fold) with medium stringency threshold (>0.40) (STRING-db.org). Strong lines indicate stronger interaction (>0.90). Key complexes are marked. B. Protein interaction analysis of the 25 down-regulated proteins with medium stringency (>0.40) (STRING-db.org). Strong lines indicate stronger interaction (>0.90). Proteins reduced by more than fivefold in patient samples are marked in red. SH3-binding proteins are emphasized.

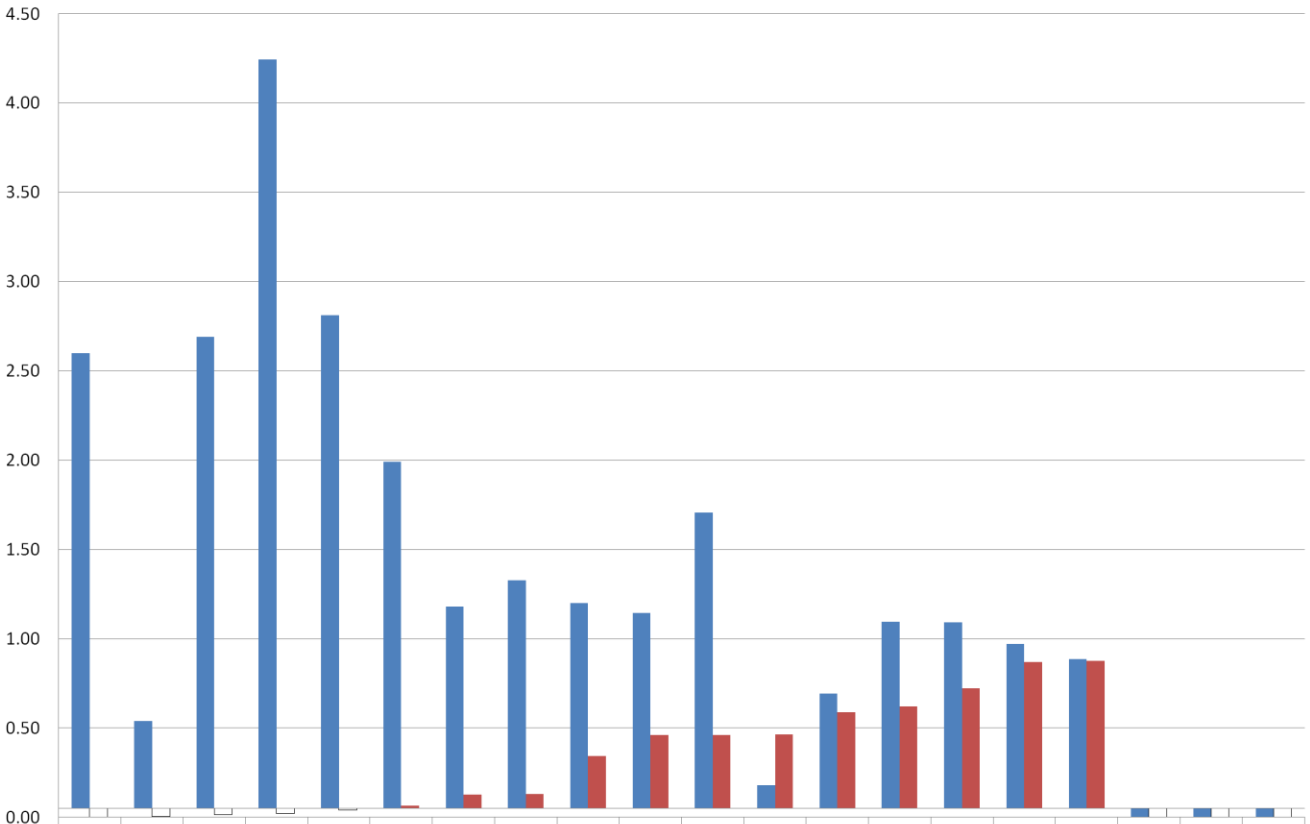

	KLK3	TGM4	FOLH1 (PSMA)	TRFL	ZA2G	LG3BP	LEG3	FAS	DHSO	NEP	KLK2	SEMG1	PSCA	AMPN	DPP4	TMPS2	PPAP	GDF15	TIMP1	KLK11
Ratio P:C	2.60	0.54	2.69	4.24	2.81	1.99	1.18	1.33	1.20	1.15	1.71	0.18	0.69	1.10	1.09	0.97	0.89	0.00	0.00	0
p-value	0.003	0.005	0.014	0.021	0.040	0.066	0.128	0.130	0.343	0.460	0.462	0.464	0.587	0.621	0.722	0.868	0.877	0.000	0.000	0.000

Fig. S6. Biomarkers found in crude urine versus EPS urine. This graph displays the prostate cancer to control ratio for the proteins found in our study that were suggested as prostate cancer markers in a recent review (Drake & Kislinger, 2014). The proteins are sorted by p-value. Five proteins (ZA2G, FOLH1, PPAP, TRFL, KLK3) passed a threshold for Fisher Exact-test at p= 0.05. Three markers described in that review (GFD15, TIMP1, KLK11) were not consistently found in this study.

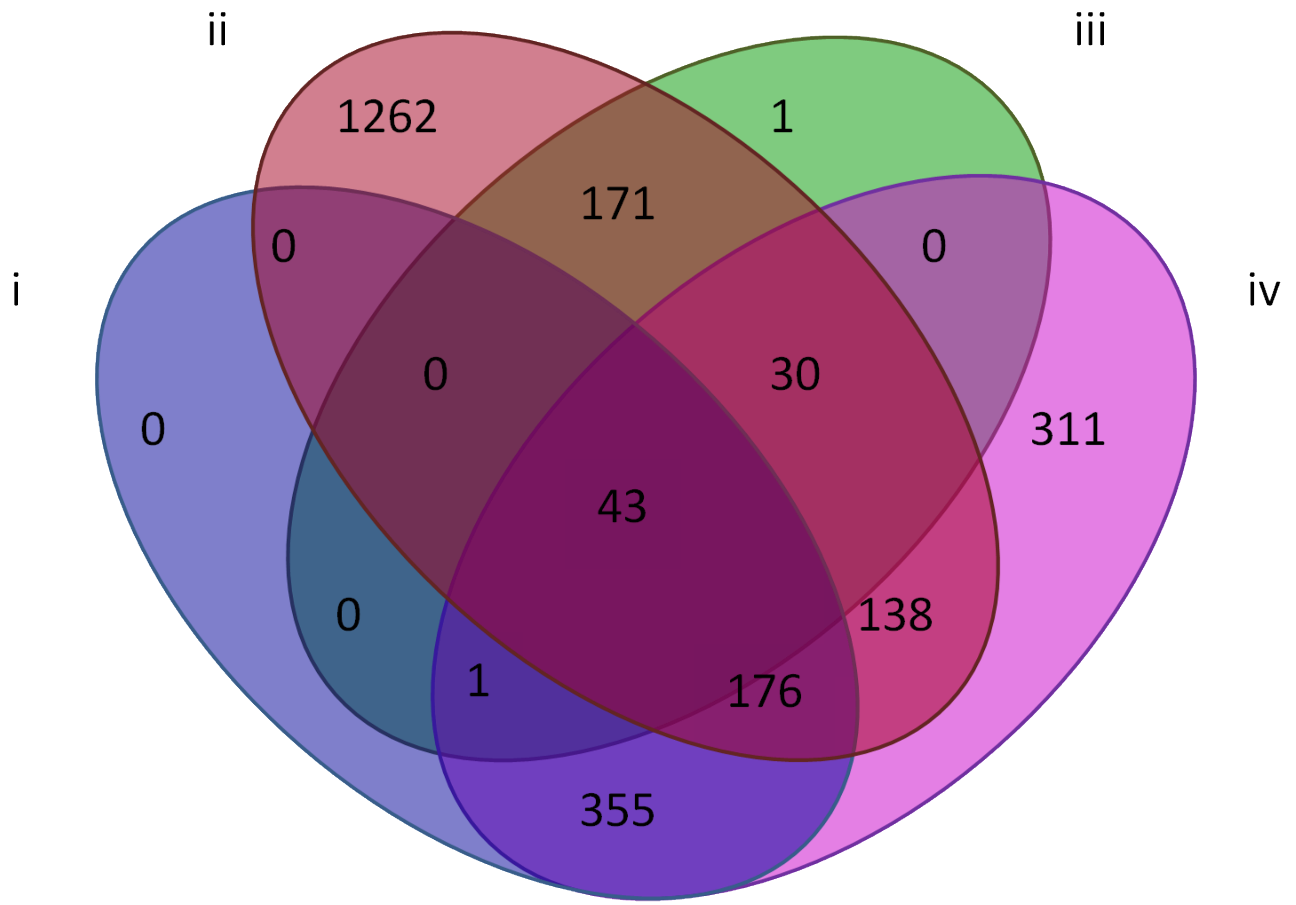

Fig. S7. Overlap of our study with a cancer proteomic database. Venn diagram showing the comparison of our study to a database for prostate cancer (http://cancerproteomics.uio.no). i) Upregulated (only) in prostate cancer (741 entries), ii) Complete urinary exosome proteome (1821 entries), iii) Significantly up-or down-regulated in prostate cancer urinary exosomes (246 entries), iv) Up-or down-regulated in prostate cancer (1586 entries).

Tables

Table S1. Cohort description. The 16 patients undergoing robot-assisted laparoscopic radical prostatectomy due to prostate cancer (PAT) and the 15 healthy urine donors (CTR) are described. Gleason score and serum PSA is shown only for patients. Gleason score 7a and 7b correspond to 3+4 and 4+3 respectively. The pH and the presence of leukocytes, nitrites, proteins, glucose, ketones and blood were analyzed with a Combur[7] Strip-Test in an Urysis 1100 urine analyzer. Only samples with low (see Materials and Methods) or not detectable (n.d.) levels of leukocytes, nitrites, proteins, glucose, ketones or blood were included in the cohort.

Table S2. List of proteins significantly changed in urinary exosomes from prostate cancer patients in alphabetical order. Full annotation of the 246 enriched proteins with Gene Name, Alternative Gene Names, Uniprot Entry Name, Uniprot Accession Number and Full Protein Name, sorted alphabetically by latter.

Table S3. Complete list of proteins significantly changed in urinary exosomes from prostate cancer patients with ratios and sensitivity for threshold set at 100% specificity. Complete list of the 246 enriched proteins with details on sensitivity at 100% specificity, number of samples (CTR and PAT) where protein was detected and ratio PAT:CTR based on iBAQ quantitative analysis of pooled samples in triplicate and relative abundance detected in same analysis (ppm of total proteome). Yellow colour show down-regulated proteins as measured by quantitative analysis, blue colour show proteins only present in patient urinary exosomes in discovery analysis.

Table S4. Heat-map for the prostate biomarkers candidates in urinary exosomes at 100% specificity. The heat-map displays all proteins that are significantly changed. Red/grey marker describes presence/absence of a specific protein above a threshold (LFQ-based) set to reach 100% specificity. Proteins that were confidently detected in patient samples only are indicated with blue, proteins significantly down-regulated in yellow. The table is sorted according to sensitivity (high-low).

Table S5. Heat-map for the prostate biomarkers candidates in urinary exosomes at maximum combined sensitivity and specificity. The heat-map contains proteins that are significantly changed at 100% specificity. Red/grey marker describes presence/absence of a specific protein above a threshold (LFQ-based) set to reach maximum combined sensitivity and specificity based on Youden's J analysis. Yellow colour show down-regulated proteins as measured by quantitative analysis, blue colour show proteins only present in patient urinary exosomes in discovery analysis. The table is sorted according to combined sensitivity and specificity (high-low).

Table S1. Cohort description

ID	*Age (yrs)*	*Gleason score*	*PSA (ng/ml)*	*Strip-test*
**PAT**				
P1	62	6	5.9	n.d.
P2	69	9	4.6	n.d.
P3	52	7a	7.9	low leu/ery
P4	66	7a	4.5	n.d.
P5	69	8	5.0	n.d.
P6	62	7b	8.7	n.d.
P7	59	7a	4.6	n.d.
P8	63	7a	23.0	n.d.
P9	56	8	13.0	n.d.
P10	69	7a	6.4	n.d.
P11	67	7a	22.0	n.d.
P12	67	7a	9.8	n.d.
P13	68	7a	6.4	n.d.
P14	55	7a	22.0	n.d.
P15	66	9	11.0	low glu/ery
P16	68	7a	9.6	n.d.
**CTR**				
C1	54	-	-	n.d.
C2	46	-	-	n.d.
C3	41	-	-	low glu/leu/ery
C4	46	-	-	low leu/ery
C5	49	-	-	low leu/ery
C6	55	-	-	n.d.
C7	41	-	-	n.d.
C8	66	-	-	n.d.
C9	42	-	-	n.d.
C10	38	-	-	n.d.
C11	41	-	-	n.d.
C12	46	-	-	n.d.
C13	43	-	-	n.d.
C14	41	-	-	n.d.
C15	46	-	-	n.d.

Table S2. Proteins differently expressed in urinary exosomes from prostate cancer patients compared to healthy individuals

#	Gene Name	Alt. Gene Name		Uniprot Entry Name	Uniprot Accession Number	Full Protein Name
1	SFN	HME1		1433S_HUMAN	P31947	14-3-3 protein sigma
2	YWHAQ			1433T_HUMAN	P27348	14-3-3 protein theta
3	PLCG2			PLCG2_HUMAN	P16885	1-phosphatidylinositol 4.5-bisphosphate phosphodiesterase gamma-2
4	DNPH1	C6orf108	RCL	DNPH1_HUMAN	O43598	2'-deoxynucleoside 5'-phosphate N-hydrolase 1
5	BDH2	DHRS6	SDR15C1	BDH2_HUMAN	Q9BUT1	3-hydroxybutyrate dehydrogenase type 2
6	ABI1	SSH3BP1		ABI1_HUMAN	Q8IZP0	Abl interactor 1
7	ASAH1	ASAH	HSD-33	ASAH1_HUMAN	Q13510	Acid ceramidase
8	ARPC4	ARC20		ARPC4_HUMAN	P59998	Actin-related protein 2/3 complex subunit 4
9	ARPC5	ARC16		ARPC5_HUMAN	O15511	Actin-related protein 2/3 complex subunit 5
10	APRT			APT_HUMAN	P07741	Adenine phosphoribosyltransferase
11	ADIRF	C10orf116	APM2	ADIRF_HUMAN	Q15847	Adipogenesis regulatory factor
12	CD38			CD38_HUMAN	P28907	ADP-ribosyl cyclase 1
13	ARF5			ARF5_HUMAN	P84085	ADP-ribosylation factor 5
14	ARL8B	ARL10C	GIE1	ARL8B_HUMAN	Q9NVJ2	ADP-ribosylation factor-like protein 8B
15	AKAP7	AKAP15	AKAP18	AKA7A_HUMAN	O43687	A-kinase anchor protein 7 isoforms alpha and beta
16	ADH5	ADHX	FDH	ADHX_HUMAN	P11766	Alcohol dehydrogenase class-3
17	ALDH1A3	ALDH6		AL1A3_HUMAN	P47895	Aldehyde dehydrogenase family 1 member A3
18	ABHD14B	CIB		ABHEB_HUMAN	Q96IU4	Alpha/beta hydrolase domain-containing protein 14B
19	ACTN1			ACTN1_HUMAN	P12814	Alpha-actinin-1
20	ANXA3	ANX3		ANXA3_HUMAN	P12429	Annexin A3
21	ANXA4	ANX4		ANXA4_HUMAN	P09525	Annexin A4
22	AQP2			AQP2_HUMAN	P41181	Aquaporin-2
23	AQP7	AQP7L	AQP9	AQP7_HUMAN	O14520	Aquaporin-7
24	ARSF			ARSF_HUMAN	P54793	Arylsulfatase F
25	GOT1			AATC_HUMAN	P17174	Aspartate aminotransferase. cytoplasmic
26	CLN3	BTS		CLN3_HUMAN	Q13286	Battenin
27	B2M	CDABP0092	HDCMA22P	B2MG_HUMAN	P61769	Beta-2-microglobulin
28	CALB1	CAB27		CALB1_HUMAN	P05937	Calbindin
29	CAB39	MO25	CGI-66	CAB39_HUMAN	Q9Y376	Calcium-binding protein 39
30	CALM1	CALM	CAM	CALM_HUMAN	P62158	Calmodulin
31	CALML3			CALL3_HUMAN	P27482	Calmodulin-like protein 3
32	CPQ	LCH1	PGCP	CBPQ_HUMAN	Q9Y646	Carboxypeptidase Q
33	CEACAM6	NCA		CEAM6_HUMAN	P40199	Carcinoembryonic antigen-related cell adhesion molecule 6
34	CTSD	CPSD		CATD_HUMAN	P07339	Cathepsin D
35	CTSZ			CATZ_HUMAN	Q9UBR2	Cathepsin Z
36	CD59	MIC11	MIN1	CD59_HUMAN	P13987	CD59 glycoprotein*
37	CD81	TAPA1	TSPAN28	CD81_HUMAN	P60033	CD81 antigen
38	CD9	MIC3	TSPAN29	CD9_HUMAN	P21926	CD9 antigen
39	CDC42SE2	SPEC2		C42S2_HUMAN	Q9NRR3	CDC42 small effector protein 2
40	TMEM30A	C6orf67	CDC50A	CC50A_HUMAN	Q9NV96	Cell cycle control protein 50A
41	CDC42			CDC42_HUMAN	P60953	Cell division control protein 42 homolog
42	CRABP2			RABP2_HUMAN	P29373	Cellular retinoic acid-binding protein 2
43	CLIC3			CLIC3_HUMAN	O95833	Chloride intracellular channel protein 3
44	CLIC6	CLIC1L		CLIC6_HUMAN	Q96NY7	Chloride intracellular channel protein 6
45	SLC44A4	C6orf29	CTL4	CTL4_HUMAN	Q53GD3	Choline transporter-like protein 4
46	SPAG9	HSS	KIAA0516	JIP4_HUMAN	O60271	C-Jun-amino-terminal kinase-interacting protein 4
47	CLDN10			CLD10_HUMAN	P78369	Claudin-10
48	CLDN2	PSEC0059	SP82	CLD2_HUMAN	P57739	Claudin-2
49	CLDN3	C7orf1	CPETR2	CLD3_HUMAN	O15551	Claudin-3
50	COL6A1			CO6A1_HUMAN	P12109	Collagen alpha-1(VI) chain
51	SPRR1A			SPR1A_HUMAN	P35321	Cornifin-A
52	ABRACL	C6orf115	HSPC280	ABRAL_HUMAN	Q9P1F3	Costars family protein ABRACL
53	CRCT1	C1orf42	NICE1	CRCT1_HUMAN	Q9UGL9	Cysteine-rich C-terminal protein 1
54	CYB561			CY561_HUMAN	P49447	Cytochrome b561
55	DMBT1	GP340		DMBT1_HUMAN	Q9UGM3	Deleted in malignant brain tumors 1 protein
56	ALAD			HEM2_HUMAN	P13716	Delta-aminolevulinic acid dehydratase
57	DSTN	ACTDP	DSN	DEST_HUMAN	P60981	Destrin
58	QDPR	DHPR		DHPR_HUMAN	P09417	Dihydropteridine reductase
59	DNM2	DYN2		DYN2_HUMAN	P50570	Dynamin-2
60	DYNLL2	DLC2		DYL2_HUMAN	Q96FJ2	Dynein light chain 2. cytoplasmic
61	LRSAM1	TAL	UNQ6496/PR	LRSM1_HUMAN	Q6UWE0	E3 ubiquitin-protein ligase LRSAM1
62	EFHD2	SWS1		EFHD2_HUMAN	Q96C19	EF-hand domain-containing protein D2
63	ENOPH1	MASA	MSTP145	ENOPH_HUMAN	Q9UHY7	Enolase-phosphatase E1
64	EIF4H	KIAA0038	WBSCR1	IF4H_HUMAN	Q15056	Eukaryotic translation initiation factor 4H
65	FABP5			FABP5_HUMAN	Q01469	Fatty acid-binding protein. epidermal
66	FTH1	FTH	FTHL6	FRIH_HUMAN	P02794	Ferritin heavy chain
67	BLVRB	FLR		BLVRB_HUMAN	P30043	Flavin reductase (NADPH)
68	FLOT1			FLOT1_HUMAN	O75955	Flotillin-1
69	FLOT2	ESA1	M17S1	FLOT2_HUMAN	Q14254	Flotillin-2
70	LGALS3BP	M2BP		LG3BP_HUMAN	Q08380	Galectin-3-binding protein
71	SNCG	BCSG1	PERSYN	SYUG_HUMAN	O76070	Gamma-synuclein
72	GMDS			GMDS_HUMAN	O60547	GDP-mannose 4.6 dehydratase
73	GMFB			GMFB_HUMAN	P60983	Glia maturation factor beta
74	GNPDA1	GNPI	HLN	GNPI1_HUMAN	P46926	Glucosamine-6-phosphate isomerase 1
75	FOLH1	FOLH	NAALAD1	FOLH1_HUMAN	Q04609	Glutamate carboxypeptidase 2
76	GSTM1	GST1		GSTM1_HUMAN	P09488	Glutathione S-transferase Mu 1
77	GSTM3	GST5		GSTM3_HUMAN	P21266	Glutathione S-transferase Mu 3
78	GSTP1	FAEES3	GST3	GSTP1_HUMAN	P09211	Glutathione S-transferase P
79	GSS			GSHB_HUMAN	P48637	Glutathione synthetase
80	GDPD3			GDPD3_HUMAN	Q7L5L3	Glycerophosphodiester phosphodiesterase domain-containing protein 3

#	Gene Name	Alt. Gene Names		Uniprot Entry Name	Uniprot Accession Number	Protein name
81	GLTP			GLTP_HUMAN	Q9NZD2	Glycolipid transfer protein
82	GOLPH3	GPP34		GOLP3_HUMAN	Q9H4A6	Golgi phosphoprotein 3
83	GCA	GCL		GRAN_HUMAN	P28676	Grancalcin
84	HRAS	HRAS1		RASH_HUMAN	P01112	GTPase HRas
85	HDHD2			HDHD2_HUMAN	Q9H0R4	Haloacid dehalogenase-like hydrolase domain-containing protein 2
86	HEBP2	C6orf34	SOUL	HEBP2_HUMAN	Q9Y5Z4	Heme-binding protein 2
87	HGSNAT	TMEM76		HGNAT_HUMAN	Q68CP4	Heparan-alpha-glucosaminide N-acetyltransferase
88	HPCAL1	BDR1		HPCL1_HUMAN	P37235	Hippocalcin-like protein 1
89	HLA-DMA	DMA	RING6	DMA_HUMAN	P28067	HLA class II histocompatibility antigen. DM alpha chain
90	HLA-DRB1	HLA-DRB2		2B1F_HUMAN	P01911	HLA class II histocompatibility antigen. DRB1-15 beta chain
91	IGHA1			IGHA1_HUMAN	P01876	Ig alpha-1 chain C region
92	IGLC2			LAC2_HUMAN (+1)	P0CG05	Ig lambda-2 chain C regions
93	GPR155	PGR22		GP155_HUMAN	Q7Z3F1	Integral membrane protein GPR155
94	ITIH4	IHRP	ITIHL1	ITIH4_HUMAN	Q14624	Inter-alpha-trypsin inhibitor heavy chain H4
95	IFITM3			IFM3_HUMAN	Q01628	Interferon-induced transmembrane protein 3
96	IDH1	PICD		IDHC_HUMAN	O75874	Isocitrate dehydrogenase [NADP] cytoplasmic
97	LTF	GIG12	LF	TRFL_HUMAN	P02788	Lactotransferrin
98	PPAP2A	LPP1		LPP1_HUMAN	O14494	Lipid phosphate phosphohydrolase 1
99	ACP1			PPAC_HUMAN	P24666	Low molecular weight phosphotyrosine protein phosphatase
100	DCXR	SDR20C1		DCXR_HUMAN	Q7Z4W1	L-xylulose reductase
101	C1orf85	PSEC0030	UNQ2553/PR(	NCUG1_HUMAN	Q8WWB7	Lysosomal protein NCU-G1
102	LAPTM4A	KIAA0108	LAPTM4	LAP4A_HUMAN	Q15012	Lysosomal-associated transmembrane protein 4A
103	SCARB2	CD36L2	LIMP2	SCRB2_HUMAN	Q14108	Lysosome membrane protein 2
104	LAMP1			LAMP1_HUMAN	P11279	Lysosome-associated membrane glycoprotein 1
105	LAMP2			LAMP2_HUMAN	P13473	Lysosome-associated membrane glycoprotein 2
106	LYZ	LZM		LYSC_HUMAN	P61626	Lysozyme C
107	MFSD12	C19orf28		MFS12_HUMAN	Q6NUT3	Major facilitator superfamily domain-containing protein 12
108	GMPPB			GMPPB_HUMAN	Q9Y5P6	Mannose-1-phosphate guanyltransferase beta
109	MMP24	MT5MMP		MMP24_HUMAN	Q9Y5R2	Matrix metalloproteinase-24
110	MLPH	SLAC2A		MELPH_HUMAN	Q9BV36	Melanophilin
111	STEAP4	STAMP2	TNFAIP9	STEA4_HUMAN	Q687X5	Metalloreductase STEAP4
112	MICALL1	KIAA1668	MIRAB13	MILK1_HUMAN	Q8N3F8	MICAL-like protein 1
113	MAP1A	MAP1L		MAP1A_HUMAN	P78559	Microtubule-associated protein 1A
114	MAPK1	ERK2	PRKM1	MK01_HUMAN	P28482	Mitogen-activated protein kinase 1
115	SLC16A4	MCT4	MCT5	MOT5_HUMAN	O15374	Monocarboxylate transporter 5
116	MTPN			MTPN_HUMAN	P58546	Myotrophin
117	MARCKS	MACS	PRKCSL	MARCS_HUMAN	P29966	Myristoylated alanine-rich C-kinase substrate
118	DDAH1	DDAH		DDAH1_HUMAN	O94760	N(G).N(G)-dimethylarginine dimethylaminohydrolase 1
119	APOA1BP	AIBP	YJEFN1	NNRE_HUMAN	Q8NCW5	NAD(P)H-hydrate epimerase
120	NAPSA	NAP1	NAPA	NAPSA_HUMAN	O96009	Napsin-A
121	SLC11A2	DCT1	DMT1	NRAM2_HUMAN	P49281	Natural resistance-associated macrophage protein 2
122	NPDC1			NPDC1_HUMAN	Q9NQX5	Neural proliferation differentiation and control protein 1
123	NCSTN	KIAA0253	UNQ1874/PR(	NICA_HUMAN	Q92542	Nicastrin
124	QPRT			NADC_HUMAN	Q15274	Nicotinate-nucleotide pyrophosphorylase [carboxylating]
125	NIT2	CUA002		NIT2_HUMAN	Q9NQR4	Omega-amidase NIT2
126	P2RX4			P2RX4_HUMAN	Q99571	P2X purinoceptor 4
127	GP2			GP2_HUMAN	P55259	Pancreatic secretory granule membrane major glycoprotein GP2
128	PALM	KIAA0270		PALM_HUMAN	O75781	Paralemmin-1
129	FKBP1A	FKBP1	FKBP12	FKB1A_HUMAN	P62942	Peptidyl-prolyl cis-trans isomerase FKBP1A
130	PGM3	AGM1		AGM1_HUMAN	O95394	Phosphoacetylglucosamine mutase
131	PMVK	PMKI		PMVK_HUMAN	Q15126	Phosphomevalonate kinase
132	PHYHD1			PHYD1_HUMAN	Q5SRE7	Phytanoyl-CoA dioxygenase domain-containing protein 1
133	SERPINA5	PCI	PLANH3	IPSP_HUMAN	P05154	Plasma serine protease inhibitor
134	LCP1	PLS2		PLSL_HUMAN	P13796	Plastin-2
135	PCYOX1	KIAA0908	PCL1	PCYOX_HUMAN	Q9UHG3	Prenylcysteine oxidase 1
136	PSEN1	AD3	PS1	PSN1_HUMAN	P49768	Presenilin-1
137	PSAP	GLBA	SAP1	SAP_HUMAN	P07602	Proactivator polypeptide
138	PNKD	KIAA1184	MR1	PNKD_HUMAN	Q8N490	Probable hydrolase PNKD
139	ZDHHC1	C16orf1	ZNF377	ZDHC1_HUMAN	Q8WTX9	Probable palmitoyltransferase ZDHHC1
140	ATP8A1	ATPIA		AT8A1_HUMAN	Q9Y2Q0	Probable phospholipid-transporting ATPase IA
141	CPVL	VLP	PSEC0124	CPVL_HUMAN	Q9H3G5	Probable serine carboxypeptidase CPVL
142	PTGR1	LTB4DH		PTGR1_HUMAN	Q14914	Prostaglandin reductase 1
143	PTGR2	ZADH1		PTGR2_HUMAN	Q8N8N7	Prostaglandin reductase 2
144	KLK3	APS		KLK3_HUMAN	P07288	Prostate-specific antigen
145	PSMA4	HC9	PSC9	PSA4_HUMAN	P25789	Proteasome subunit alpha type-4
146	PSMA5			PSA5_HUMAN	P28066	Proteasome subunit alpha type-5
147	PSMA7	HSPC		PSA7_HUMAN	O14818	Proteasome subunit alpha type-7
148	PSMB2			PSB2_HUMAN	P49721	Proteasome subunit beta type-2
149	BRK1	C3orf10	HSPC300	BRK1_HUMAN	Q8WUW1	Protein BRICK1
150	CUTA	ACHAP	C6orf82	CUTA_HUMAN	O60888	Protein CutA
151	PARK7			PARK7_HUMAN	Q99497	Protein DJ-1
152	DOPEY2	C21orf5	KIAA0933	DOP2_HUMAN	Q9Y3R5	Protein dopey-2
153	TMBIM1	LFG3	RECS1	LFG3_HUMAN	Q969X1	Protein lifeguard 3
154	FAM129A	C1orf24	NIBAN	NIBAN_HUMAN	Q9BZQ8	Protein Niban
155	S100A1	S100A		S10A1_HUMAN	P23297	Protein S100-A1
156	S100A6	CACY		S10A6_HUMAN	P06703	Protein S100-A6
157	S100A9	CAGB	CFAG	S10A9_HUMAN	P06702	Protein S100-A9
158	S100P	S100E		S100P_HUMAN	P25815	Protein S100-P
159	SPNS1	SPIN1	PP20300	SPNS1_HUMAN	Q9H2V7	Protein spinster homolog 1
160	TTYH3	KIAA1691		TTYH3_HUMAN	Q9C0H2	Protein tweety homolog 3

#	Gene Name	Alt. Gene Names		Uniprot Entry Name	Uniprot Accession Number	Protein name
161	SLC2A13			MYCT_HUMAN	Q96QE2	Proton myo-inositol cotransporter
162	PNP	NP		PNPH_HUMAN	P00491	Purine nucleoside phosphorylase
163	CRYZ			QOR_HUMAN	Q08257	Quinone oxidoreductase
164	LAMTOR1	C11orf59	PDRO	LTOR1_HUMAN	Q6IAA8	Ragulator complex protein LAMTOR1
165	LAMTOR2	MAPBPIP	ROBLD3	LTOR2_HUMAN	Q9Y2Q5	Ragulator complex protein LAMTOR2
166	LAMTOR3	MAP2K1IP1	MAPKSP1	LTOR3_HUMAN	Q9UHA4	Ragulator complex protein LAMTOR3
167	LAMTOR4	C7orf59		LTOR4_HUMAN	Q0VGL1	Ragulator complex protein LAMTOR4
168	LAMTOR5	HBXIP	XIP	LTOR5_HUMAN	O43504	Ragulator complex protein LAMTOR5
169	RAB10			RAB10_HUMAN	P61026	Ras-related protein Rab-10
170	RAB12			RAB12_HUMAN	Q6IQ22	Ras-related protein Rab-12
171	RAB14			RAB14_HUMAN	P61106	Ras-related protein Rab-14
172	RAB17			RAB17_HUMAN	Q9H0T7	Ras-related protein Rab-17
173	RAB18			RAB18_HUMAN	Q9NP72	Ras-related protein Rab-18
174	RAB1A	RAB1		RAB1A_HUMAN	P62820	Ras-related protein Rab-1A
175	RAB1B			RAB1B_HUMAN	Q9H0U4	Ras-related protein Rab-1B
176	RAB27B			RB27B_HUMAN	O00194	Ras-related protein Rab-27B
177	RAB2A	RAB2		RAB2A_HUMAN	P61019	Ras-related protein Rab-2A
178	RAB35	RAB1C	RAY	RAB35_HUMAN	Q15286	Ras-related protein Rab-35
179	RAB3B			RAB3B_HUMAN	P20337	Ras-related protein Rab-3B
180	RAB3D	GOV	RAB16	RAB3D_HUMAN	O95716	Ras-related protein Rab-3D
181	RAB43	RAB41		RAB43_HUMAN	Q86YS6	Ras-related protein Rab-43
182	RAB6A	RAB6		RAB6A_HUMAN	P20340	Ras-related protein Rab-6A
183	RAB7A	RAB7		RAB7A_HUMAN	P51149	Ras-related protein Rab-7a
184	RAB8A	MEL	RAB8	RAB8A_HUMAN	P61006	Ras-related protein Rab-8A
185	RAB8B			RAB8B_HUMAN	Q92930	Ras-related protein Rab-8B
186	RAB9A	RAB9		RAB9A_HUMAN	P51151	Ras-related protein Rab-9A
187	RBP5			RET5_HUMAN	P82980	Retinol-binding protein 5
188	NQO2	NMOR2		NQO2_HUMAN	P16083	Ribosyldihydronicotinamide dehydrogenase [quinone]
189	SCAMP1	SCAMP		SCAM1_HUMAN	O15126	Secretory carrier-associated membrane protein 1
190	SCAMP2			SCAM2_HUMAN	O15127	Secretory carrier-associated membrane protein 2
191	SPR			SPRE_HUMAN	P35270	Sepiapterin reductase
192	SEPTIN2	DIFF6	KIAA0158	SEPT2_HUMAN	Q15019	Septin-2
193	SERINC2	TDE2L	FKSG84	SERC2_HUMAN	Q96SA4	Serine incorporator 2
194	PPP2CA			PP2AA_HUMAN	P67775	Serine/threonine-protein phosphatase 2A catalytic subunit alpha isoform
195	STAM	STAM1		STAM1_HUMAN	Q92783	Signal transducing adapter molecule 1
196	STAM2	HBP		STAM2_HUMAN	O75886	Signal transducing adapter molecule 2
197	SMIM22			SIM22_HUMAN	K7EJ46	Small integral membrane protein 22
198	SMIM5	C17orf109		SMIM5_HUMAN	Q71RC9	Small integral membrane protein 5
199	SLC5A1	NAGT	SGLT1	SC5A1_HUMAN	P13866	Sodium/glucose cotransporter 1
200	SLC34A2			NPT2B_HUMAN	O95436	Sodium-dependent phosphate transport protein 2B
201	SLC15A1	PEPT1		S15A1_HUMAN	P46059	Solute carrier family 15 member 1
202	SLC35F2			S35F2_HUMAN	Q8IXU6	Solute carrier family 35 member F2
203	SLC35F6	C2orf18	UNQ3047/PR(	S35F6_HUMAN	Q8N357	Solute carrier family 35 member F6
204	SRI			SORCN_HUMAN	P30626	Sorcin
205	SARG	C1orf116		SARG_HUMAN	Q9BW04	Specifically androgen-regulated gene protein
206	SMS			SPSY_HUMAN	P52788	Spermine synthase
207	SKP1	EMC19	OCP2	SKP1_HUMAN	P63208	S-phase kinase-associated protein 1
208	CTTN	EMS1		SRC8_HUMAN	Q14247	Src substrate cortactin
209	SI			SUIS_HUMAN	P14410	Sucrase-isomaltase. intestinal
210	SYNGR2	UNQ352/PRO615		SNG2_HUMAN	O43760	Synaptogyrin-2
211	SYT7	PCANAP7		SYT7_HUMAN	O43581	Synaptotagmin-7
212	SYTL4			SYTL4_HUMAN	Q96C24	Synaptotagmin-like protein 4
213	STXBP4			STXB4_HUMAN	Q6ZWJ1	Syntaxin-binding protein 4
214	CCT5	CCTE	KIAA0098	TCPE_HUMAN	P48643	T-complex protein 1 subunit epsilon
215	TSPAN6	TM4SF6	UNQ767/PRO	TSN6_HUMAN	O43657	Tetraspanin-6
216	TSPAN8	TM4SF3		TSN8_HUMAN	P19075	Tetraspanin-8
217	TXNDC17	TXNL5		TXD17_HUMAN	Q9BRA2	Thioredoxin domain-containing protein 17
218	TSTD1	KAT		TSTD1_HUMAN	Q8NFU3	Thiosulfate sulfurtransferase/rhodanese-like domain-containing protein 1
219	TMSB4X	TB4X	THYB4	TYB4_HUMAN	P62328	Thymosin beta-4
220	TPT1			TCTP_HUMAN	P13693	Translationally-controlled tumor protein
221	TM7SF3			TM7S3_HUMAN	Q9NS93	Transmembrane 7 superfamily member 3
222	TMPRSS2	PRSS10		TMPS2_HUMAN	O15393	Transmembrane protease serine 2
223	TMEM106B			T106B_HUMAN	Q9NUM4	Transmembrane protein 106B
224	TMEM176A	HCA112		T176A_HUMAN	Q96HP8	Transmembrane protein 176A
225	TMEM256	C17orf61		TM256_HUMAN	Q8N2U0	Transmembrane protein 256
226	TMEM63A	KIAA0489	KIAA0792	TM63A_HUMAN	O94886	Transmembrane protein 63A
227	TMEM8A	TMEM6	TMEM8	TMM8A_HUMAN	Q9HCN3	Transmembrane protein 8A
228	TPM4			TPM4_HUMAN	P67936	Tropomyosin alpha-4 chain
229	TUBB2B			TBB2B_HUMAN	Q9BVA1	Tubulin beta-2B chain
230	TPD52			TPD52_HUMAN	P55327	Tumor protein D52
231	LCK			LCK_HUMAN	P06239	Tyrosine-protein kinase Lck
232	UBE2K	HIP2	LIG	UBE2K_HUMAN	P61086	Ubiquitin-conjugating enzyme E2 K
233	UBE2V2	MMS2	UEV2	UB2V2_HUMAN	Q15819	Ubiquitin-conjugating enzyme E2 variant 2
234	UGDH			UGDH_HUMAN	O60701	UDP-glucose 6-dehydrogenase
235	C6orf132			CF132_HUMAN	Q5T0Z8	Uncharacterized protein C6orf132
236	MYO5C			MYO5C_HUMAN	Q9NQX4	Unconventional myosin-Vc
237	UPK1A	TSPAN21		UPK1A_HUMAN	O00322	Uroplakin-1a
238	VPS37C	PML39		VP37C_HUMAN	A5D8V6	Vacuolar protein sorting-associated protein 37C
239	VPS25	DERP9	EAP20	VPS25_HUMAN	Q9BRG1	Vacuolar protein-sorting-associated protein 25
240	VAMP2	SYB2		VAMP2_HUMAN	P63027	Vesicle-associated membrane protein 2
241	VDAC1	VDAC		VDAC1_HUMAN	P21796	Voltage-dependent anion-selective channel protein 1
242	ATP6V0C	ATP6C	ATP6L	VATL_HUMAN	P27449	V-type proton ATPase 16 kDa proteolipid subunit
243	ATP6V0D1	ATP6D	VPATPD	VA0D1_HUMAN	P61421	V-type proton ATPase subunit d 1
244	ATP6V1F	ATP6S14	VATF	VATF_HUMAN	Q16864	V-type proton ATPase subunit F
245	ZNF185			ZN185_HUMAN	O15231	Zinc finger protein 185
246	AZGP1	ZAG	ZNGP1	ZA2G_HUMAN	P25311	Zinc-alpha-2-glycoprotein

Table S3. Complete list of proteins enriched in prostate cancer exosomes			Data from Discovery analysis			Data from Quantitative Validation analysis	
#	Uniprot Accession Number	Protein name	Sensitivity	#Present in CTR (of 15)	#Present i PAT (of 16)	Validated iBAQ ratio PAT:CTR	Abundance (ppm of total proteome)
**1**	Q8N2U0	Transmembrane protein 256	94 %	5	16	**140.39**	4324
**2**	Q15847	Adipogenesis regulatory factor	81 %	4	15	**18.99**	369
**3**	Q6IAA8	Ragulator complex protein LAMTOR1	81 %	4	16	**22.98**	201
**4**	P13796	Plastin-2	75 %	14	16	**3.15**	256
**5**	P61019	Ras-related protein Rab-2A	75 %	14	16	**3.55**	1083
**6**	P20337	Ras-related protein Rab-3B	75 %	15	16	**2.69**	1138
**7**	O95716	Ras-related protein Rab-3D	75 %	15	16	**2.24**	2340
**8**	P51149	Ras-related protein Rab-7a	75 %	15	16	**3.26**	2317
**9**	P27449	V-type proton ATPase 16 kDa proteolipid subunit	75 %	0	12	**3.55**	861
**10**	Q687X5	Metalloreductase STEAP4	69 %	14	16	**2.97**	953
**11**	Q99497	Protein DJ-1	69 %	15	16	**1.92**	957
**12**	P25815	Protein S100-P	69 %	14	15	**1.84**	1351
**13**	Q96C24	Synaptotagmin-like protein 4	69 %	5	12	**3.08**	91
**14**	Q9NVJ2	ADP-ribosylation factor-like protein 8B	63 %	13	15	**2.79**	49
**15**	Q96QE2	Proton myo-inositol cotransporter	63 %	2	11	**2.66**	100
**16**	P20340	Ras-related protein Rab-6A	63 %	10	16	**3.36**	240
**17**	O43657	Tetraspanin-6	63 %	9	16	**4.03**	3067
**18**	P78369	Claudin-10	56 %	7	14	**2.14**	26
**19**	P57739	Claudin-2	56 %	2	12	**3.00**	69
**20**	O15551	Claudin-3	56 %	1	10	**1.75**	170
**21**	O60547	GDP-mannose 4.6 dehydratase	56 %	2	12	**2.45**	16
**22**	P46926	Glucosamine-6-phosphate isomerase 1	56 %	4	13	**15.51**	44
**23**	Q14108	Lysosome membrane protein 2	56 %	15	16	**3.94**	824
**24**	Q6NUT3	Major facilitator superfamily domain-containing protein 12	56 %	5	15	**8.07**	65
**25**	Q9BV36	Melanophilin	56 %	12	16	**2.26**	151
**26**	P35270	Sepiapterin reductase	56 %	10	14	**2.16**	114
**27**	Q9BRA2	Thioredoxin domain-containing protein 17	56 %	15	16	**2.35**	288
**28**	Q9BUT1	3-hydroxybutyrate dehydrogenase type 2	50 %	14	16	**2.26**	389
**29**	P62158	Calmodulin	50 %	15	16	**6.30**	4764
**30**	Q9Y646	Carboxypeptidase Q	50 %	1	8	**5.80**	51
**31**	Q14254	Flotillin-2	50 %	14	16	**2.89**	541
**32**	Q08380	Galectin-3-binding protein	50 %	15	16	**1.99**	678
**33**	Q99571	P2X purinoceptor 4	50 %	6	13	**2.36**	76
**34**	Q9Y3R5	Protein dopey-2	50 %	12	16	**2.99**	218
**35**	P06703	Protein S100-A6	50 %	15	16	**0.48**	1853
**36**	Q15286	Ras-related protein Rab-35	50 %	15	16	**0.57**	245
**37**	P67775	Serine/threonine-protein phosphatase 2A catalytic subunit alpha isoform	50 %	11	14	**16.91**	41
**38**	O43598	2'-deoxynucleoside 5'-phosphate N-hydrolase 1	44 %	7	14	**2.26**	117
**39**	Q13510	Acid ceramidase	44 %	15	16	**3.73**	967
**40**	P05937	Calbindin	44 %	15	16	**2.02**	1907
**41**	P13987	CD59 glycoprotein*	44 %	15	16	**0.00**	2
**42**	P60033	CD81 antigen	44 %	13	15	**2.61**	918
**43**	P49447	Cytochrome b561	44 %	1	11	**24.50**	86
**44**	Q9UHY7	Enolase-phosphatase E1	44 %	1	7	**2.66**	55
**45**	Q9H4A6	Golgi phosphoprotein 3	44 %	1	15	**1.86**	9
**46**	Q92542	Nicastrin	44 %	10	16	**2.37**	170
**47**	Q9H3G5	Probable serine carboxypeptidase CPVL	44 %	1	7	**6.25**	75
**48**	O43504	Ragulator complex protein LAMTOR5	44 %	0	7	**2.40**	192
**49**	O00194	Ras-related protein Rab-27B	44 %	15	16	**2.24**	1850
**50**	O15127	Secretory carrier-associated membrane protein 2	44 %	0	7	**3.51**	230
**51**	P52788	Spermine synthase	44 %	8	14	**2.46**	143
**52**	P63208	S-phase kinase-associated protein 1	44 %	10	16	**7.68**	12
**53**	Q9NS93	Transmembrane 7 superfamily member 3	44 %	15	15	**4.15**	198
**54**	P55327	Tumor protein D52	44 %	0	7	**13.69**	34
**55**	Q15819	Ubiquitin-conjugating enzyme E2 variant 2	44 %	11	15	**2.10**	289
**56**	O60701	UDP-glucose 6-dehydrogenase	44 %	13	16	**4.11**	249
**57**	P25311	Zinc-alpha-2-glycoprotein	44 %	8	14	**2.49**	942
**58**	Q7L5L3	Glycerophosphodiester phosphodiesterase domain-containing protein 3	41 %	2	16	**6.31**	40
59	Q96C19	EF-hand domain-containing protein D2	63 %	13	15	1.75	71
60	P61106	Ras-related protein Rab-14	63 %	15	16	2.34	975
61	Q9NQR4	Omega-amidase NIT2	63 %	13	16	**1.58**	393
62	P12814	Alpha-actinin-1	56 %	8	12	**1.33**	33652
63	O15374	Monocarboxylate transporter 5	56 %	0	9	**1.33**	383
64	Q6IQ22	Ras-related protein Rab-12	56 %	14	15	**1.57**	22
65	P61006	Ras-related protein Rab-8A	56 %	15	16	**1.24**	323
66	O94886	Transmembrane protein 63A	56 %	13	16	**1.71**	69
67	P61769	Beta-2-microglobulin	50 %	4	9	0.20	13
68	P61421	V-type proton ATPase subunit d 1	50 %	14	16	2.48	235
69	O14494	Lipid phosphate phosphohydrolase 1	50 %	15	16	4.26	441
70	Q7Z3F1	Integral membrane protein GPR155	50 %	1	9	11.64	9
71	P31947	14-3-3 protein sigma	50 %	14	16	**0.78**	215
72	Q6UWE0	E3 ubiquitin-protein ligase LRSAM1	50 %	0	8	**1.30**	15
73	P28067	HLA class II histocompatibility antigen. DM alpha chain	50 %	7	7	**1.67**	295
74	P51151	Ras-related protein Rab-9A	50 %	3	9	**1.51**	68
75	Q14247	Src substrate cortactin	50 %	12	16	**0.69**	101
76	O14520	Aquaporin-7	44 %	6	11	2.08	248
77	O76070	Gamma-synuclein	44 %	11	14	2.43	183
78	P27348	14-3-3 protein theta	44 %	15	16	**0.70**	175
79	P17174	Aspartate aminotransferase. cytoplasmic	44 %	15	16	**1.55**	539
80	O95833	Chloride intracellular channel protein 3	44 %	11	13	**1.30**	156
81	P60981	Destrin	44 %	15	16	**1.49**	639
82	P01112	GTPase HRas	44 %	2	8	**1.60**	65
83	Q8N8N7	Prostaglandin reductase 2	44 %	2	7	**1.34**	94
84	P48643	T-complex protein 1 subunit epsilon	44 %	9	14	**1.39**	13
85	Q14624	Inter-alpha-trypsin inhibitor heavy chain H4	40 %	6	0	**0.23**	1
86	P47895	Aldehyde dehydrogenase family 1 member A3	38 %	6	14	**4.56**	97
87	P12429	Annexin A3	38 %	15	16	**2.80**	802
88	Q13286	Battenin	38 %	1	9	**7.26**	23

#	Uniprot Accession Number	Protein name	Sensitivity	#Present in CTR (of 15)	#Present i PAT (of 16)	Validated iBAQ ratio PAT:CTR	Abundance (ppm of total proteome)
89	P07339	Cathepsin D	38 %	5	16	**4.37**	284
90	O94760	N(G).N(G)-dimethylarginine dimethylaminohydrolase 1	38 %	15	16	**1.84**	742
91	Q9NQX5	Neural proliferation differentiation and control protein 1	38 %	2	8	**4.88**	9
92	P07602	Proactivator polypeptide	38 %	10	14	**2.23**	115
93	P07288	Prostate-specific antigen	38 %	15	16	**2.61**	783
94	Q969X1	Protein lifeguard 3	38 %	15	16	**3.32**	2745
95	Q9BZQ8	Protein Niban	38 %	11	16	**3.70**	210
96	Q9H2V7	Protein spinster homolog 1	38 %	1	7	**4.38**	27
97	Q9Y2Q5	Ragulator complex protein LAMTOR2	38 %	0	6	**4.57**	226
98	Q9UHA4	Ragulator complex protein LAMTOR3	38 %	0	6	**7.91**	36
99	O43581	Synaptotagmin-7	38 %	7	14	**3.90**	240
100	Q9NUM4	Transmembrane protein 106B	38 %	0	6	**2.53**	81
101	Q9NQX4	Unconventional myosin-Vc	38 %	2	11	***15.50***	9
102	P63027	Vesicle-associated membrane protein 2	38 %	14	16	**2.23**	367
103	Q16864	V-type proton ATPase subunit F	38 %	7	14	**3.18**	57
104	O43687	A-kinase anchor protein 7 isoforms alpha and beta	31 %	3	13	**2.56**	24
105	P54793	Arylsulfatase F	31 %	4	13	**5.79**	75
106	O60271	C-Jun-amino-terminal kinase-interacting protein 4	31 %	5	12	**2.50**	34
107	Q9UGM3	Deleted in malignant brain tumors 1 protein	31 %	7	12	**14.32**	136
108	P60983	Glia maturation factor beta	31 %	1	10	**2.52**	72
109	Q04609	Glutamate carboxypeptidase 2	31 %	13	16	**2.69**	382
110	P48637	Glutathione synthetase	31 %	12	14	**3.05**	161
111	P37235	Hippocalcin-like protein 1	31 %	13	12	**1.79**	116
112	P29966	Myristoylated alanine-rich C-kinase substrate	31 %	15	16	**0.38**	239
113	Q8NCW5	NAD(P)H-hydrate epimerase	31 %	7	12	**2.53**	226
114	O96009	Napsin-A	31 %	15	16	**2.83**	1638
115	O95394	Phosphoacetylglucosamine mutase	31 %	8	13	**4.08**	62
116	Q9Y2Q0	Probable phospholipid-transporting ATPase IA	31 %	4	12	**3.35**	50
117	Q08257	Quinone oxidoreductase	31 %	8	14	**2.24**	175
118	P82980	Retinol-binding protein 5	31 %	14	16	**2.36**	928
119	Q96SA4	Serine incorporator 2	31 %	15	16	**20.18**	56
120	P46059	Solute carrier family 15 member 1	31 %	0	5	**2.23**	39
121	P30626	Sorcin	31 %	14	16	**3.22**	2828
122	P14410	Sucrase-isomaltase. intestinal	31 %	1	5	**2.74**	4
123	P59998	Actin-related protein 2/3 complex subunit 4	25 %	15	16	**2.35**	634
124	O15511	Actin-related protein 2/3 complex subunit 5	25 %	15	16	**8.06**	165
125	P07741	Adenine phosphoribosyltransferase	25 %	14	16	**2.20**	356
126	Q9P1F3	Costars family protein ABRACL	25 %	5	13	**8.38**	375
127	P01876	Ig alpha-1 chain C region	25 %	14	16	**4.75**	593
128	Q01628	Interferon-induced transmembrane protein 3	25 %	7	15	**10.13**	265
129	P02788	Lactotransferrin **	25 %	3	11	**41.74**	626
130	P00491	Purine nucleoside phosphorylase	25 %	9	16	**2.39**	244
131	Q6ZWJ1	Syntaxin-binding protein 4	25 %	4	10	**2.23**	43
132	P19075	Tetraspanin-8	25 %	12	16	**0.51**	131
133	Q9BRG1	Vacuolar protein-sorting-associated protein 25	25 %	14	16	**3.06**	819
134	P21796	Voltage-dependent anion-selective channel protein 1	25 %	9	16	**6.97**	116
135	P12109	Collagen alpha-1(VI) chain	0 %	7	2	**0.00**	4
136	Q92783	Signal transducing adapter molecule 1	0 %	12	13	**0.63**	90
137	P21926	CD9 antigen	19 %	15	16	**2.03**	15238
138	O75955	Flotillin-1	19 %	15	16	**1.98**	589
139	P28676	Grancalcin	19 %	7	7	**2.09**	723
140	Q9Y5P6	Mannose-1-phosphate guanyltransferase beta	19 %	5	14	**2.40**	71
141	O14818	Proteasome subunit alpha type-7	19 %	13	13	**3.10**	244
142	Q9NP72	Ras-related protein Rab-18	19 %	13	16	**1.98**	243
143	A5D8V6	Vacuolar protein sorting-associated protein 37C	19 %	15	16	**2.09**	761
144	O75886	Signal transducing adapter molecule 2	0 %	14	11	**0.59**	51
145	P55259	Pancreatic secretory granule membrane major glycoprotein GP2	13 %	4	11	**9.07**	11
146	P16885	1-phosphatidylinositol 4.5-bisphosphate phosphodiesterase gamma-2	0 %	14	11	**0.49**	40
147	P13473	Lysosome-associated membrane glycoprotein 2	6 %	15	16	**2.81**	1924
148	Q9UGL9	Cysteine-rich C-terminal protein 1	0 %	11	3	**0.30**	21
149	P05154	Plasma serine protease inhibitor	0 %	15	11	**0.41**	80
150	P06239	Tyrosine-protein kinase Lck	0 %	7	2	**0.04**	35
151	Q0VGL1	Ragulator complex protein LAMTOR4***	xx	1	5	**7.38**	159
152	O15126	Secretory carrier-associated membrane protein 1	56 %	2	10	*3.88*	nd
153	P62942	Peptidyl-prolyl cis-trans isomerase FKBP1A	56 %	15	16	1.27	559
154	Q96HP8	Transmembrane protein 176A	50 %	2	10	*3.61*	nd
155	P62328	Thymosin beta-4	50 %	15	16	1.14	2890
156	Q9H0R4	Haloacid dehalogenase-like hydrolase domain-containing protein 2	50 %	1	16	1.16	62
157	P60953	Cell division control protein 42 homolog	50 %	15	16	1.20	775
158	Q9H0T7	Ras-related protein Rab-17	50 %	10	14	1.23	99
159	Q96NY7	Chloride intracellular channel protein 6	50 %	15	16	1.24	217
160	Q53GD3	Choline transporter-like protein 4	50 %	15	16	1.26	1366
161	P30043	Flavin reductase (NADPH)	50 %	15	16	1.27	439
162	P61026	Ras-related protein Rab-10	50 %	15	16	1.41	2078
163	Q9Y5Z4	Heme-binding protein 2	50 %	15	11	1.46	264
164	Q01469	Fatty acid-binding protein. epidermal	50 %	9	14	1.53	54
165	Q71RC9	Small integral membrane protein 5	50 %	13	13	1.64	3277
166	Q15012	Lysosomal-associated transmembrane protein 4A	50 %	0	8	*INF*	nd
167	Q5SRE7	Phytanoyl-CoA dioxygenase domain-containing protein 1	50 %	0	8	*INF*	nd
168	P28066	Proteasome subunit alpha type-5	50 %	0	8	*INF*	nd

#	Uniprot Accession Number	Protein name	Sensitivity	#Present in CTR (of 15)	#Present i PAT (of 16)	Validated iBAQ ratio PAT:CTR	Abundance (ppm of total proteome)
169	P27482	Calmodulin-like protein 3	44 %	4	8	*4.79*	nd
170	P49768	Presenilin-1	44 %	1	7	*14.95*	nd
171	P16083	Ribosyldihydronicotinamide dehydrogenase [quinone]	44 %	5	13	1.03	70
172	P13693	Translationally-controlled tumor protein	44 %	10	16	1.20	244
173	P11279	Lysosome-associated membrane glycoprotein 1	44 %	15	16	1.38	1279
174	P28907	ADP-ribosyl cyclase 1	44 %	7	13	1.53	28
175	P58546	Myotrophin	44 %	15	16	*INF*	nd
176	Q96FJ2	Dynein light chain 2. cytoplasmic	38 %	10	14	1.95	34
177	P09417	Dihydropteridine reductase	38 %	15	16	2.02	122
178	Q15274	Nicotinate-nucleotide pyrophosphorylase [carboxylating]	38 %	4	10	4.34	21
179	Q9NV96	Cell cycle control protein 50A	38 %	8	10	**1.26**	48
180	Q15126	Phosphomevalonate kinase	38 %	5	8	**0.70**	16
181	Q15056	Eukaryotic translation initiation factor 4H	31 %	11	15	1.86	155
182	Q9C0H2	Protein tweety homolog 3	31 %	15	16	2.11	132
183	O95436	Sodium-dependent phosphate transport protein 2B	31 %	2	11	2.72	9
184	P0CG05	Ig lambda-2 chain C regions	31 %	4	9	3.28	31
185	P29373	Cellular retinoic acid-binding protein 2	31 %	15	15	3.63	389
186	O60888	Protein CutA	31 %	6	12	4.03	104
187	P25789	Proteasome subunit alpha type-4	31 %	4	10	6.11	13
188	Q8N357	Solute carrier family 35 member F6	31 %	3	11	6.24	25
189	P13716	Delta-aminolevulinic acid dehydratase	31 %	10	14	**1.73**	63
190	Q7Z4W1	L-xylulose reductase	31 %	15	16	**1.58**	496
191	O00322	Uroplakin-1a	31 %	15	16	**1.53**	3427
192	P35321	Cornifin-A	25 %	8	14	2.64	177
193	O15231	Zinc finger protein 185	25 %	5	10	4.49	1
194	Q9HCN3	Transmembrane protein 8A	25 %	1	7	6.47	6
195	Q9UHG3	Prenylcysteine oxidase 1	25 %	1	13	8.22	24
196	P61626	Lysozyme C	25 %	10	16	23.11	1449
197	O75781	Paralemmin-1	19 %	2	9	3.70	3
198	P40199	Carcinoembryonic antigen-related cell adhesion molecule 6	19 %	5	8	5.05	352
199	P13866	Sodium/glucose cotransporter 1	19 %	15	16	**1.75**	472
200	Q14914	Prostaglandin reductase 1	13 %	15	16	**1.57**	1125
201	P06702	Protein S100-A9	13 %	10	14	**1.31**	1928
202	Q8N3F8	MICAL-like protein 1	6 %	4	6	3.41	1
203	Q96IU4	Alpha/beta hydrolase domain-containing protein 14B	6 %	14	16	**0.93**	652
204	P41181	Aquaporin-2	6 %	15	16	**1.59**	6762
205	P09211	Glutathione S-transferase P	6 %	15	16	**1.71**	2655
206	Q8WTX9	Probable palmitoyltransferase ZDHHC1	0 %	15	15	0.57	175
207	Q92930	Ras-related protein Rab-8B	56 %	14	16	*1.30*	nd
208	O15393	Transmembrane protease serine 2	44 %	15	16	*1.16*	nd
209	Q9H0U4	Ras-related protein Rab-1B	44 %	15	16	*1.35*	nd
210	P62820	Ras-related protein Rab-1A	44 %	15	16	*1.47*	nd
211	Q86YS6	Ras-related protein Rab-43	38 %	2	8	*7.56*	nd
212	O43760	Synaptogyrin-2	38 %	1	6	*8.43*	nd
213	P01911	HLA class II histocompatibility antigen. DRB1-15 beta chain	38 %	1	10	*9.13*	nd
214	Q8WWB7	Lysosomal protein NCU-G1	38 %	1	6	*23.59*	nd
215	Q9Y376	Calcium-binding protein 39	38 %	15	16	0.89	1074
216	P50570	Dynamin-2	38 %	13	16	0.98	96
217	Q9NRR3	CDC42 small effector protein 2	38 %	8	14	1.20	775
218	P02794	Ferritin heavy chain	38 %	11	14	1.61	655
219	Q8IXU6	Solute carrier family 35 member F2	38 %	0	6	*INF*	nd
220	Q8N490	Probable hydrolase PNKD	0 %	10	6	1.52	9
221	Q9UBR2	Cathepsin Z	31 %	5	10	*2.52*	nd
222	Q9BVA1	Tubulin beta-2B chain	31 %	2	9	*7.48*	nd
223	Q8NFU3	Thiosulfate sulfurtransferase/rhodanese-like domain-containing protein 1	31 %	11	15	0.93	132
224	P28482	Mitogen-activated protein kinase 1	31 %	9	16	1.01	54
225	P11766	Alcohol dehydrogenase class-3	31 %	11	16	1.12	178
226	P24666	Low molecular weight phosphotyrosine protein phosphatase	31 %	7	15	1.31	52
227	P09525	Annexin A4	31 %	15	16	1.36	1971
228	Q15019	Septin-2	31 %	14	16	1.44	92
229	P21266	Glutathione S-transferase Mu 3	31 %	15	16	1.46	781
230	Q8WUW1	Protein BRICK1	31 %	1	8	1.48	41
231	P49721	Proteasome subunit beta type-2	31 %	0	5	*INF*	nd
232	P61086	Ubiquitin-conjugating enzyme E2 K	31 %	0	5	*INF*	nd
233	P23297	Protein S100-A1	25 %	6	11	*2.46*	nd
234	P78559	Microtubule-associated protein 1A	25 %	1	7	*4.87*	nd
235	P09488	Glutathione S-transferase Mu 1	25 %	10	14	1.34	381
236	Q9Y5R2	Matrix metalloproteinase-24	0 %	14	5	*0.39*	nd
237	K7EJ46	Small integral membrane protein 22	19 %	10	14	*2.05*	nd
238	Q68CP4	Heparan-alpha-glucosaminide N-acetyltransferase	19 %	3	16	*4.28*	nd
239	Q9BW04	Specifically androgen-regulated gene protein	19 %	15	16	1.06	335
240	Q8IZP0	Abl interactor 1	19 %	15	16	1.09	190
241	Q5T0Z8	Uncharacterized protein C6orf132	19 %	4	8	1.16	8
242	P84085	ADP-ribosylation factor 5	13 %	6	12	0.91	56
243	O75874	Isocitrate dehydrogenase [NADP] cytoplasmic	13 %	15	16	1.22	2420
244	Q9NZD2	Glycolipid transfer protein	13 %	14	10	1.48	167
245	P67936	Tropomyosin alpha-4 chain	6 %	9	14	*2.47*	nd
246	P49281	Natural resistance-associated macrophage protein 2	6 %	10	16	*1.63*	nd

Notes:

Yellow indicates down-regulated protein

Blue indicates protein only found in patient exosome samples

Column: Validated iBAQ ratio PAT:CTR

Bold = found significantly altered (p<0.05) in PAT in validation analysis

Normal = found in validation analysis (p>0.05), (iBAQ ratio)

Italics = not found in validation analysis, values from discovery analysis (TOP3TIC ratio

INF = Infinite; only found in PAT samples

Column: Abundance

Abundance: for PAT:CTR>1.0; total detected protein amount in PAT, for PAT:CTR<1.0; total detected protein amount in CTF

nd = not detecte Infinite; only found in PAT samples

TABLE S4 Heatmap for enriched proteins at 100% specificity

Gene	Uniprot	Identified Proteins
TMEM256	Q8N2U0	Transmembrane protein 256
ADIRF	Q15847	Adipogenesis regulatory factor
LAMTOR1	Q6IAA8	Ragulator complex protein LAMTOR1
ATP6V0C	P27449	V-type proton ATPase 16 kDa proteolipid subunit
RAB7A	P51149	Ras-related protein Rab-7a
RAB3D	O95716	Ras-related protein Rab-3D
RAB3B	P20337	Ras-related protein Rab-3B
LCP1	P13796	Plastin-2
RAB2A	P61019	Ras-related protein Rab-2A
PARK7	Q99497	Protein DJ-1
S100P	P25815	Protein S100-P
STEAP4	Q687X5	Metalloreductase STEAP4
SYTL4	Q96C24	Synaptotagmin-like protein 4
ARL8B	Q9NVJ2	ADP-ribosylation factor-like protein 8B
RAB14	P61106	Ras-related protein Rab-14
EFHD2	Q96C19	EF-hand domain-containing protein D2
TSPAN6	O43657	Tetraspanin-6
NIT2	Q9NQR4	Omega-amidase NIT2
RAB6A	P20340	Ras-related protein Rab-6A
SLC2A13	Q96QE2	Proton myo-inositol cotransporter
SLC16A4	O15374	Monocarboxylate transporter 5
SPR	P35270	Sepiapterin reductase
GMDS	O60547	GDP-mannose 4.6 dehydratase
CLDN3	O15551	Claudin-3
RAB12	Q6IQ22	Ras-related protein Rab-12
FKBP1A	P62942	Peptidyl-prolyl cis-trans isomerase FKBP1A
RAB8A	P61006	Ras-related protein Rab-8A
MFSD12	Q6NUT3	Major facilitator superfamily domain-containing protein 12
SCAMP1	O15126	Secretory carrier-associated membrane protein 1
MLPH	Q9BV36	Melanophilin
RAB8B	Q92930	Ras-related protein Rab-8B
TXNDC17	Q9BRA2	Thioredoxin domain-containing protein 17
TMEM63A	O94886	Transmembrane protein 63A
ACTN1	P12814	Alpha-actinin-1
SCARB2	Q14108	Lysosome membrane protein 2
CLDN2	P57739	Claudin-2
GNPDA1	P46926	Glucosamine-6-phosphate isomerase 1
CLDN10	P78369	Claudin-10
LAPTM4A	Q15012	Lysosomal-associated transmembrane protein 4A
LRSAM1	Q6UWE0	E3 ubiquitin-protein ligase LRSAM1
PHYHD1	Q5SRE7	Phytanoyl-CoA dioxygenase domain-containing protein 1
PSMA5	P28066	Proteasome subunit alpha type-5
CPQ	Q9Y646	Carboxypeptidase Q
RAB35	Q15286	Ras-related protein Rab-35
GPR155	Q7Z3F1	Integral membrane protein GPR155
DBI	P07108	Acyl-CoA-binding protein
S100A6	P06703	Protein S100-A6
TMSB4X	P62328	Thymosin beta-4
BDH2	Q9BUT1	3-hydroxybutyrate dehydrogenase type 2
TMEM176A	Q96HP8	Transmembrane protein 176A
RAB9A	P51151	Ras-related protein Rab-9A
B2M	P61769	Beta-2-microglobulin
SMIM5	Q71RC9	Small integral membrane protein 5
CALM1	P62158	Calmodulin
FABP5	Q01469	Fatty acid-binding protein. epidermal
PPP2CA	P67775	Serine/threonine-protein phosphatase 2A catalytic subunit alpha isoform
CDC42	P60953	Cell division control protein 42 homolog
P2RX4	Q99571	P2X purinoceptor 4
HDHD2	Q9H0R4	Haloacid dehalogenase-like hydrolase domain-containing protein 2
RAB10	P61026	Ras-related protein Rab-10
LGALS3BP	Q08380	Galectin-3-binding protein
RAB17	Q9H0T7	Ras-related protein Rab-17
BLVRB	P30043	Flavin reductase (NADPH)
ATP6V0D1	P61421	V-type proton ATPase subunit d 1
CLIC6	Q96NY7	Chloride intracellular channel protein 6
DOPEY2	Q9Y3R5	Protein dopey-2
PPAP2A	O14494	Lipid phosphate phosphohydrolase 1
HEBP2	Q9Y5Z4	Heme-binding protein 2
SCAMP2	O15127	Secretory carrier-associated membrane protein 2
TPD52	P55327	Tumor protein D52
CPVL	Q9H3G5	Probable serine carboxypeptidase CPVL
ENOPH1	Q9UHY7	Enolase-phosphatase E1
PSEN1	P49768	Presenilin-1
LAMTOR5	O43504	Ragulator complex protein LAMTOR5
HRAS	P01112	GTPase HRas
CYB561	P49447	Cytochrome b561
GOT1	P17174	Aspartate aminotransferase. cytoplasmic
CD38	P28907	ADP-ribosyl cyclase 1
SNCG	O76070	Gamma-synuclein
CD59	P13987	CD59 glycoprotein
CALB1	P05937	Calbindin
DNPH1	O43598	2'-deoxynucleoside 5'-phosphate N-hydrolase 1
TM7SF3	Q9NS93	Transmembrane 7 superfamily member 3

MTPN	P58546	Myotrophin	○	○	○	○	○	○	○	○	○	○	○	○	○	○	○	●	●	●	●	○	○	○	●	○	○	●	○	○	●	○	○
AQP7	O14520	Aquaporin-7	○	○	○	○	○	○	○	○	○	○	○	○	○	○	○	●	●	○	●	○	●	○	●	●	○	○	○	○	○	○	●
SKP1	P63208	S-phase kinase-associated protein 1	○	○	○	○	○	○	○	○	○	○	○	○	○	○	○	●	●	○	○	○	○	○	●	○	●	○	○	○	●	●	●
NCSTN	Q92542	Nicastrin	○	○	○	○	○	○	○	○	○	○	○	○	○	○	○	●	●	○	○	○	○	○	●	●	○	●	○	○	●	●	○
TMPRSS2	O15393	Transmembrane protease serine 2	○	○	○	○	○	○	○	○	○	○	○	○	○	○	○	●	●	○	○	○	○	○	●	○	●	●	○	○	●	●	○
TPT1	P13693	Translationally-controlled tumor protein	○	○	○	○	○	○	○	○	○	○	○	○	○	○	○	●	●	○	●	○	○	○	●	○	○	●	○	○	●	●	○
YWHAQ	P27348	14-3-3 protein theta	○	○	○	○	○	○	○	○	○	○	○	○	○	○	○	●	●	○	○	○	○	○	●	○	○	○	●	○	●	●	●
SMS	P52788	Spermine synthase	○	○	○	○	○	○	○	○	○	○	○	○	○	○	○	●	○	○	●	○	○	○	●	○	○	●	●	○	●	●	○
ITIH4	Q14624	Inter-alpha-trypsin inhibitor heavy chain H4	●	○	○	●	○	●	●	●	●	○	○	○	○	○	○	○	○	○	○	○	○	○	○	○	○	○	○	○	○	○	○
HLA-DMA	P28067	HLA class II histocompatibility antigen. DM alpha chain	○	○	○	○	○	○	○	○	○	○	○	○	○	○	○	●	●	●	○	○	○	○	○	○	○	●	○	○	●	○	●
FLOT2	Q14254	Flotillin-2	○	○	○	○	○	○	○	○	○	○	○	○	○	○	○	●	●	●	○	○	○	○	○	●	○	○	○	○	●	○	●
SFN	P31947	14-3-3 protein sigma	○	○	○	○	○	○	○	○	○	○	○	○	○	○	○	●	○	○	○	○	○	○	●	○	○	●	●	○	●	○	●
PTGR2	Q8N8N7	Prostaglandin reductase 2	○	○	○	○	○	○	○	○	○	○	○	○	○	○	○	○	●	○	○	○	○	○	●	○	○	●	●	○	●	●	○
CALML3	P27482	Calmodulin-like protein 3	○	○	○	○	○	○	○	○	○	○	○	○	○	○	○	●	○	○	●	○	●	●	○	●	○	●	○	○	○	○	○
ASAH1	Q13510	Acid ceramidase	○	○	○	○	○	○	○	○	○	○	○	○	○	○	○	●	●	○	●	○	○	○	●	●	○	○	○	○	●	○	○
NQO2	P16083	Ribosyldihydronicotinamide dehydrogenase [quinone]	○	○	○	○	○	○	○	○	○	○	○	○	○	○	○	●	●	○	●	○	●	○	○	●	○	●	○	○	○	○	○
LAMP1	P11279	Lysosome-associated membrane glycoprotein 1	○	○	○	○	○	○	○	○	○	○	○	○	○	○	○	●	○	●	○	○	○	○	●	○	○	○	○	○	●	●	●
RAB1A	P62820	Ras-related protein Rab-1A	○	○	○	○	○	○	○	○	○	○	○	○	○	○	○	○	○	○	●	○	○	○	●	○	○	○	●	○	●	●	●
RAB1B	Q9H0U4	Ras-related protein Rab-1B	○	○	○	○	○	○	○	○	○	○	○	○	○	○	○	○	○	○	●	○	○	○	●	○	○	○	●	○	●	●	●
AZGP1	P25311	Zinc-alpha-2-glycoprotein	○	○	○	○	○	○	○	○	○	○	○	○	○	○	○	○	○	○	●	○	○	●	○	○	●	●	●	○	○	●	○
CLIC3	O95833	Chloride intracellular channel protein 3	○	○	○	○	○	○	○	○	○	○	○	○	○	○	○	●	●	●	○	○	○	○	●	●	○	○	○	○	●	○	○
UBE2V2	Q15819	Ubiquitin-conjugating enzyme E2 variant 2	○	○	○	○	○	○	○	○	○	○	○	○	○	○	○	○	●	○	●	○	○	○	○	●	●	●	○	○	○	●	○
CCT5	P48643	T-complex protein 1 subunit epsilon	○	○	○	○	○	○	○	○	○	○	○	○	○	○	○	●	○	○	●	○	○	○	●	○	●	○	○	○	●	●	○
UGDH	O60701	UDP-glucose 6-dehydrogenase	○	○	○	○	○	○	○	○	○	○	○	○	○	○	○	○	○	○	●	○	○	●	○	○	●	●	○	○	●	●	○
DSTN	P60981	Destrin	○	○	○	○	○	○	○	○	○	○	○	○	○	○	○	●	●	○	○	○	○	○	●	○	○	○	○	○	●	●	●
LAMTOR2	Q9Y2Q5	Ragulator complex protein LAMTOR2	○	○	○	○	○	○	○	○	○	○	○	○	○	○	○	●	●	○	●	○	○	○	○	●	○	○	○	○	●	○	●
LAMTOR3	Q9UHA4	Ragulator complex protein LAMTOR3	○	○	○	○	○	○	○	○	○	○	○	○	○	○	○	●	●	○	○	○	○	○	○	●	○	●	○	○	●	○	●
SLC35F2	Q8IXU6	Solute carrier family 35 member F2	○	○	○	○	○	○	○	○	○	○	○	○	○	○	○	●	●	○	○	●	○	○	○	○	○	●	○	○	○	●	●
TMEM106B	Q9NUM4	Transmembrane protein 106B	○	○	○	○	○	○	○	○	○	○	○	○	○	○	○	●	●	○	○	○	○	○	●	●	○	●	○	○	○	○	●
C1orf85	Q8WWB7	Lysosomal protein NCU-G1	○	○	○	○	○	○	○	○	○	○	○	○	○	○	○	●	●	○	○	○	○	○	●	●	○	●	○	○	○	○	●
SPNS1	Q9H2V7	Protein spinster homolog 1	○	○	○	○	○	○	○	○	○	○	○	○	○	○	○	●	●	○	○	○	○	●	○	●	○	●	○	○	○	○	●
HLA-DRB1	P01911	HLA class II histocompatibility antigen. DRB1-15 beta chain	○	○	○	○	○	○	○	○	○	○	○	○	○	○	○	●	○	●	○	○	○	○	●	●	●	○	○	○	●	○	○
SYNGR2	O43760	Synaptogyrin-2	○	○	○	○	○	○	○	○	○	○	○	○	○	○	○	●	●	○	●	●	○	○	○	○	●	●	○	○	○	○	○
PMVK	Q15126	Phosphomevalonate kinase	○	○	○	○	○	○	○	○	○	○	○	○	○	○	○	○	○	○	○	○	○	○	●	●	●	●	●	○	●	○	○
ALDH1A3	P47895	Aldehyde dehydrogenase family 1 member A3	○	○	○	○	○	○	○	○	○	○	○	○	○	○	○	○	○	○	●	○	○	●	●	○	●	●	○	○	○	●	○
CLN3	Q13286	Battenin	○	○	○	○	○	○	○	○	○	○	○	○	○	○	○	●	●	●	○	○	○	○	●	○	○	●	○	○	●	○	○
QPRT	Q15274	Nicotinate-nucleotide pyrophosphorylase [carboxylating]	○	○	○	○	○	○	○	○	○	○	○	○	○	○	○	●	●	○	●	○	○	●	●	○	○	○	○	○	●	○	○
CDC42SE2	Q9NRR3	CDC42 small effector protein 2	○	○	○	○	○	○	○	○	○	○	○	○	○	○	○	●	●	○	○	○	○	○	●	○	○	○	○	●	●	○	●
RAB43	Q86YS6	Ras-related protein Rab-43	○	○	○	○	○	○	○	○	○	○	○	○	○	○	○	●	●	○	○	○	○	○	●	●	○	●	○	○	○	○	●
NPDC1	Q9NQX5	Neural proliferation differentiation and control protein 1	○	○	○	○	○	○	○	○	○	○	○	○	○	○	○	○	○	○	●	○	○	○	●	○	○	●	●	●	○	●	○
QDPR	P09417	Dihydropteridine reductase	○	○	○	○	○	○	○	○	○	○	○	○	○	○	○	●	○	○	●	○	○	○	○	●	○	●	○	○	●	●	○
HLA-DRB1	Q29974	HLA class II histocompatibility antigen. DRB1-16 beta chain	○	○	○	○	○	○	○	○	○	○	○	○	○	○	○	●	○	○	○	○	○	○	●	●	○	●	○	●	●	○	○
SYT7	O43581	Synaptotagmin-7	○	○	○	○	○	○	○	○	○	○	○	○	○	○	○	○	○	○	●	○	○	○	●	○	●	●	○	○	●	●	○
DYNLL2	Q96FJ2	Dynein light chain 2. cytoplasmic	○	○	○	○	○	○	○	○	○	○	○	○	○	○	○	●	○	○	●	○	○	○	○	○	○	●	●	○	●	●	○
PSAP	P07602	Proactivator polypeptide	○	○	○	○	○	○	○	○	○	○	○	○	○	○	○	●	●	●	○	○	○	○	○	○	○	●	○	○	●	○	●
DNM2	P50570	Dynamin-2	○	○	○	○	○	○	○	○	○	○	○	○	○	○	○	●	●	○	●	○	○	○	●	○	○	○	○	○	●	●	○
KLK3	P07288	Prostate-specific antigen	○	○	○	○	○	○	○	○	○	○	○	○	○	○	○	○	○	○	●	○	○	○	○	○	●	●	●	○	●	●	○
PNKD	Q8N490	Probable hydrolase PNKD	○	○	○	○	○	●	○	○	●	○	○	●	○	●	●	○	○	○	○	○	○	○	○	○	○	○	○	○	○	○	○
CTTN	Q14247	Src substrate cortactin	○	○	○	○	○	○	○	○	○	○	○	○	○	○	○	●	●	○	○	○	○	○	●	○	○	○	○	○	●	●	○
SLC44A4	Q53GD3	Choline transporter-like protein 4	○	○	○	○	○	○	○	○	○	○	○	○	○	○	○	○	●	●	○	○	○	○	●	○	○	○	○	○	●	○	●
GOLPH3	Q9H4A6	Golgi phosphoprotein 3	○	○	○	○	○	○	○	○	○	○	○	○	○	○	○	○	○	○	●	○	○	○	●	○	●	●	○	○	●	○	○
RAB27B	O00194	Ras-related protein Rab-27B	○	○	○	○	○	○	○	○	○	○	○	○	○	○	○	○	○	○	●	○	○	○	●	○	○	●	●	○	●	○	○
CD81	P60033	CD81 antigen	○	○	○	○	○	○	○	○	○	○	○	○	○	○	○	●	●	●	●	○	○	○	○	○	○	○	○	○	○	●	○
MYO5C	Q9NQX4	Unconventional myosin-Vc	○	○	○	○	○	○	○	○	○	○	○	○	○	○	○	○	○	○	○	○	○	○	●	○	○	●	●	○	●	●	○
FTH1	P02794	Ferritin heavy chain	○	○	○	○	○	○	○	○	○	○	○	○	○	○	○	●	○	○	○	○	○	○	●	○	○	○	○	○	●	●	●
TMEM30A	Q9NV96	Cell cycle control protein 50A	○	○	○	○	○	○	○	○	○	○	○	○	○	○	○	○	○	●	○	○	○	●	●	○	●	○	○	○	●	○	○
VAMP2	P63027	Vesicle-associated membrane protein 2	○	○	○	○	○	○	○	○	○	○	○	○	○	○	○	●	○	○	●	○	○	○	○	○	●	●	○	○	○	●	○
TMBIM1	Q969X1	Protein lifeguard 3	○	○	○	○	○	○	○	○	○	○	○	○	○	○	○	●	●	○	○	○	○	○	●	●	○	○	○	○	○	○	●
CTSD	P07339	Cathepsin D	○	○	○	○	○	○	○	○	○	○	○	○	○	○	○	●	○	○	○	○	○	○	●	●	○	●	○	○	●	○	○
DDAH1	O94760	N(G).N(G)-dimethylarginine dimethylaminohydrolase 1	○	○	○	○	○	○	○	○	○	○	○	○	○	○	○	●	●	○	○	○	○	○	○	○	●	●	○	○	●	○	○
FAM129A	Q9BZQ8	Protein Niban	○	○	○	○	○	○	○	○	○	○	○	○	○	○	○	○	○	○	○	○	○	○	●	○	●	●	○	○	●	●	○
CAB39	Q9Y376	Calcium-binding protein 39	○	○	○	○	○	○	○	○	○	○	○	○	○	○	○	●	●	○	●	○	○	○	●	○	○	○	○	○	●	○	○
ATP6V1F	Q16864	V-type proton ATPase subunit F	○	○	○	○	○	○	○	○	○	○	○	○	○	○	○	●	●	○	○	○	○	○	○	●	○	●	○	○	●	○	○
PSMB2	P49721	Proteasome subunit beta type-2	○	○	○	○	○	○	○	○	○	○	○	○	○	○	○	○	●	○	○	○	○	○	●	○	○	●	○	○	●	●	○
SLC15A1	P46059	Solute carrier family 15 member 1	○	○	○	○	○	○	○	○	○	○	○	○	○	○	○	●	○	○	○	●	○	○	●	●	○	●	○	○	○	○	○
UBE2K	P61086	Ubiquitin-conjugating enzyme E2 K	○	○	○	○	○	○	○	○	○	○	○	○	○	○	○	○	●	○	○	○	○	○	●	○	○	●	○	○	●	●	○
SI	P14410	Sucrase-isomaltase. intestinal	○	○	○	○	○	○	○	○	○	○	○	○	○	○	○	○	○	●	●	●	○	○	○	○	○	●	○	○	○	●	○
PSMA4	P25789	Proteasome subunit alpha type-4	○	○	○	○	○	○	○	○	○	○	○	○	○	○	○	○	○	○	●	○	○	○	●	○	○	●	○	○	●	●	○
CUTA	O60888	Protein CutA	○	○	○	○	○	○	○	○	○	○	○	○	○	○	○	○	○	○	●	○	○	○	○	○	●	●	○	○	●	●	○
SRI	P30626	Sorcin	○	○	○	○	○	○	○	○	○	○	○	○	○	○	○	○	●	○	○	○	○	○	●	●	○	●	○	○	○	○	●
SLC34A2	O95436	Sodium-dependent phosphate transport protein 2B	○	○	○	○	○	○	○	○	○	○	○	○	○	○	○	○	●	●	○	○	●	○	○	○	○	○	○	○	●	○	●
AKAP7	O43687	A-kinase anchor protein 7 isoforms alpha and beta	○	○	○	○	○	○	○	○	○	○	○	○	○	○	○	○	○	○	○	●	○	○	○	○	●	●	○	○	●	●	○
ACP1	P24666	Low molecular weight phosphotyrosine protein phosphatase	○	○	○	○	○	○	○	○	○	○	○	○	○	○	○	○	○	○	●	○	○	●	●	○	●	○	○	○	●	○	○
SLC35F6	Q8N357	Solute carrier family 35 member F6	○	○	○	○	○	○	○	○	○	○	○	○	○	○	○	●	●	○	○	○	○	○	○	●	○	●	○	○	○	○	●
ATP8A1	Q9Y2Q0	Probable phospholipid-transporting ATPase IA	○	○	○	○	○	○	○	○	○	○	○	○	○	○	○	○	○	○	○	●	○	○	○	○	●	●	●	○	○	●	○
CRYZ	Q08257	Quinone oxidoreductase	○	○	○	○	○	○	○	○	○	○	○	○	○	○	○	●	●	○	○	○	○	○	●	○	○	○	●	○	●	○	○
EIF4H	Q15056	Eukaryotic translation initiation factor 4H	○	○	○	○	○	○	○	○	○	○	○	○	○	○	○	●	●	○	●	○	○	○	●	○	○	○	○	○	●	○	○
CTSZ	Q9UBR2	Cathepsin Z	○	○	○	○	○	○	○	○	○	○	○	○	○	○	○	●	●	○	○	○	○	○	●	●	○	○	○	○	●	○	○
TTYH3	Q9C0H2	Protein tweety homolog 3	○	○	○	○	○	○	○	○	○	○	○	○	○	○	○	●	●	○	○	○	○	○	●	○	○	●	○	○	●	○	○
SPAG9	O60271	C-Jun-amino-terminal kinase-interacting protein 4	○	○	○	○	○	○	○	○	○	○	○	○	○	○	○	●	○	○	○	○	○	○	●	●	○	○	○	○	●	○	●
RBP5	P82980	Retinol-binding protein 5	○	○	○	○	○	○	○	○	○	○	○	○	○	○	○	●	●	○	●	○	○	○	●	○	○	○	○	○	●	○	○
NAPSA	O96009	Napsin-A	○	○	○	○	○	○	○	○	○	○	○	○	○	○	○	●	●	○	○	○	○	○	●	●	○	○	○	○	●	○	○
CRABP2	P29373	Cellular retinoic acid-binding protein 2	○	○	○	○	○	○	○	○	○	○	○	○	○	○	○	●	●	○	○	○	○	○	●	○	○	○	○	○	●	●	○
ANXA4	P09525	Annexin A4	○	○	○	○	○	○	○	○	○	○	○	○	○	○	○	●	●	○	○	○	○	○	●	○	○	○	○	○	●	●	○
UPK1A	O00322	Uroplakin-1a	○	○	○	○	○	○	○	○	○	○	○	○	○	○	○	●	●	○	○	○	○	●	○	●	○	○	○	○	●	○	○
APOA1BP	Q8NCW5	NAD(P)H-hydrate epimerase	○	○	○	○	○	○	○	○	○	○	○	○	○	○	○	○	●	○	○	●	○	○	●	○	○	○	○	○	●	●	○
TSTD1	Q8NFU3	Thiosulfate sulfurtransferase/rhodanese-like domain-containing protein 1	○	○	○	○	○	○	○	○	○	○	○	○	○	○	○	○	○	○	●	○	○	○	●	○	○	●	○	○	●	●	○

FOLH1	Q04609	Glutamate carboxypeptidase 2	○	○	○	○	○	○	○	○	○	○	○	○	○	○	○	●	○	○	○	○	○	○	○	○	●	●	○	○	●	●	○
SEPTIN2	Q15019	Septin-2	○	○	○	○	○	○	○	○	○	○	○	○	○	○	○	●	○	●	●	○	○	○	●	○	○	○	○	○	●	○	○
MARCKS	P29966	Myristoylated alanine-rich C-kinase substrate	○	○	○	○	○	○	○	○	○	○	○	○	○	○	○	●	●	○	○	○	○	○	○	○	○	○	○	○	●	●	●
ANXA3	P12429	Annexin A3	○	○	○	○	○	○	○	○	○	○	○	○	○	○	○	○	○	○	○	○	○	○	○	○	●	●	○	○	●	●	○
GMFB	P60983	Glia maturation factor beta	○	○	○	○	○	○	○	○	○	○	○	○	○	○	○	●	●	○	○	○	○	○	○	○	○	○	○	○	●	●	○
PGM3	O95394	Phosphoacetylglucosamine mutase	○	○	○	○	○	○	○	○	○	○	○	○	○	○	○	○	○	○	○	●	○	○	○	○	●	●	○	○	●	○	○
ALAD	P13716	Delta-aminolevulinic acid dehydratase	○	○	○	○	○	○	○	○	○	○	○	○	○	○	○	●	○	○	●	○	○	○	○	○	○	○	○	○	●	●	○
TUBB2B	Q9BVA1	Tubulin beta-2B chain	○	○	○	○	○	○	○	○	○	○	○	○	○	○	○	○	○	○	●	○	○	○	○	○	○	○	●	○	●	●	○
DMBT1	Q9UGM3	Deleted in malignant brain tumors 1 protein	○	○	○	○	○	○	○	○	○	○	○	○	○	○	○	●	○	●	○	○	●	○	○	○	○	○	○	●	○	○	○
IGLC2	P0CG05	Ig lambda-2 chain C regions	○	○	○	○	○	○	○	○	○	○	○	○	○	○	○	○	○	●	○	○	●	○	○	○	○	○	○	○	●	●	○
ARSF	P54793	Arylsulfatase F	○	○	○	○	○	○	○	○	○	○	○	○	○	○	○	●	○	○	○	○	○	○	●	●	○	○	○	○	●	○	○
DCXR	Q7Z4W1	L-xylulose reductase	○	○	○	○	○	○	○	○	○	○	○	○	○	○	○	●	●	○	○	○	○	○	●	○	○	○	○	○	●	○	○
HPCAL1	P37235	Hippocalcin-like protein 1	○	○	○	○	○	○	○	○	○	○	○	○	○	○	○	●	●	○	○	○	○	○	○	○	○	○	○	●	●	○	○
SERINC2	Q96SA4	Serine incorporator 2	○	○	○	○	○	○	○	○	○	○	○	○	○	○	○	○	●	○	○	○	○	○	●	○	○	○	○	○	●	●	○
MAPK1	P28482	Mitogen-activated protein kinase 1	○	○	○	○	○	○	○	○	○	○	○	○	○	○	○	○	○	○	●	○	○	○	●	○	○	○	○	○	●	●	○
ADH5	P11766	Alcohol dehydrogenase class-3	○	○	○	○	○	○	○	○	○	○	○	○	○	○	○	●	○	○	○	○	○	○	●	○	○	○	○	○	●	●	○
GSTM3	P21266	Glutathione S-transferase Mu 3	○	○	○	○	○	○	○	○	○	○	○	○	○	○	○	●	●	○	○	○	○	○	●	○	○	○	○	○	●	○	○
PCYOX1	Q9UHG3	Prenylcysteine oxidase 1	○	○	○	○	○	○	○	○	○	○	○	○	○	○	○	●	●	○	○	○	○	○	○	●	○	○	○	○	●	○	○
STXBP4	Q6ZWJ1	Syntaxin-binding protein 4	○	○	○	○	○	○	○	○	○	○	○	○	○	○	○	○	●	○	●	○	○	○	●	○	○	○	○	○	●	○	○
TMEM8A	Q9HCN3	Transmembrane protein 8A	○	○	○	○	○	○	○	○	○	○	○	○	○	○	○	○	●	○	○	○	○	○	○	○	○	●	○	○	●	○	●
LTF	P02788	Lactotransferrin	○	○	○	○	○	○	○	○	○	○	○	○	○	○	○	○	○	●	○	○	○	○	○	○	●	○	○	●	○	●	○
IGHA1	P01876	Ig alpha-1 chain C region	○	○	○	○	○	○	○	○	○	○	○	○	○	○	○	○	○	●	○	○	●	○	○	○	○	○	○	●	●	○	○
ABRACL	Q9P1F3	Costars family protein ABRACL	○	○	○	○	○	○	○	○	○	○	○	○	○	○	○	○	○	○	●	○	○	○	●	○	○	●	○	●	○	○	○
LYZ	P61626	Lysozyme C	○	○	○	○	○	○	○	○	○	○	○	○	○	○	○	○	○	○	○	○	○	●	○	●	○	●	○	●	○	○	○
APRT	P07741	Adenine phosphoribosyltransferase	○	○	○	○	○	○	○	○	○	○	○	○	○	○	○	●	○	○	○	○	○	○	●	○	○	○	○	○	●	●	○
PNP	P00491	Purine nucleoside phosphorylase	○	○	○	○	○	○	○	○	○	○	○	○	○	○	○	●	●	○	○	○	○	○	○	●	○	○	○	○	●	○	○
TSPAN8	P19075	Tetraspanin-8	○	○	○	○	○	○	○	○	○	○	○	○	○	○	○	○	○	○	●	○	○	○	●	●	●	○	○	○	○	○	○
SPRR1A	P35321	Cornifin-A	○	○	○	○	○	○	○	○	○	○	○	○	○	○	○	●	●	○	○	○	○	○	○	○	○	○	○	○	●	●	○
S100A1	P23297	Protein S100-A1	○	○	○	○	○	○	○	○	○	○	○	○	○	○	○	●	○	○	○	○	○	●	○	○	○	●	○	○	●	○	○
GSTM1	P09488	Glutathione S-transferase Mu 1	○	○	○	○	○	○	○	○	○	○	○	○	○	○	○	●	●	○	●	○	○	○	○	○	○	○	○	○	○	●	○
COL6A1	P12109	Collagen alpha-1(VI) chain	○	○	○	●	○	○	○	●	○	○	○	●	○	○	○	○	○	○	○	○	○	○	○	○	○	○	○	○	○	○	○
MMP24	Q9Y5R2	Matrix metalloproteinase-24	○	●	●	○	○	○	○	●	○	○	○	○	○	○	○	○	○	○	○	○	○	○	○	○	○	○	○	○	○	○	○
STAM	Q92783	Signal transducing adapter molecule 1	○	●	○	○	○	○	●	○	○	○	○	●	○	○	○	○	○	○	○	○	○	○	○	○	○	○	○	○	○	○	○
GSS	P48637	Glutathione synthetase	○	○	○	○	○	○	○	○	○	○	○	○	○	○	○	○	○	○	○	○	○	○	●	○	○	●	○	○	●	○	○
VPS25	Q9BRG1	Vacuolar protein-sorting-associated protein 25	○	○	○	○	○	○	○	○	○	○	○	○	○	○	○	○	●	○	○	○	○	○	○	○	○	○	○	○	●	○	●
ARPC4	P59998	Actin-related protein 2/3 complex subunit 4	○	○	○	○	○	○	○	○	○	○	○	○	○	○	○	●	○	○	○	○	○	○	●	○	○	○	○	○	●	○	○
ZNF185	O15231	Zinc finger protein 185	○	○	○	○	○	○	○	○	○	○	○	○	○	○	○	○	○	●	○	○	○	○	●	○	○	○	○	○	○	●	○
IFITM3	Q01628	Interferon-induced transmembrane protein 3	○	○	○	○	○	○	○	○	○	○	○	○	○	○	○	●	○	●	○	○	○	○	○	○	○	○	○	○	●	○	○
VDAC1	P21796	Voltage-dependent anion-selective channel protein 1	○	○	○	○	○	○	○	○	○	○	○	○	○	○	○	●	●	○	○	○	○	○	○	○	○	○	○	○	●	○	○
ARPC5	O15511	Actin-related protein 2/3 complex subunit 5	○	○	○	○	○	○	○	○	○	○	○	○	○	○	○	○	○	●	○	○	○	○	●	○	○	○	○	○	●	○	○
C6orf132	Q5T0Z8	Uncharacterized protein C6orf132	○	○	○	○	○	○	○	○	○	○	○	○	○	○	○	●	○	●	○	○	○	○	○	○	○	○	○	○	●	○	○
HGSNAT	Q68CP4	Heparan-alpha-glucosaminide N-acetyltransferase	○	○	○	○	○	○	○	○	○	○	○	○	○	○	○	○	●	○	○	○	○	○	●	●	○	○	○	○	○	○	○
SARG	Q9BW04	Specifically androgen-regulated gene protein	○	○	○	○	○	○	○	○	○	○	○	○	○	○	○	●	○	○	○	○	○	○	●	○	○	○	○	○	●	○	○
PSMA7	O14818	Proteasome subunit alpha type-7	○	○	○	○	○	○	○	○	○	○	○	○	○	○	○	○	○	○	●	○	○	○	○	○	●	●	○	○	○	○	○
CEACAM6	P40199	Carcinoembryonic antigen-related cell adhesion molecule 6	○	○	○	○	○	○	○	○	○	○	○	○	○	○	○	●	○	●	○	○	○	○	○	○	○	●	○	○	○	○	○
GMPPB	Q9Y5P6	Mannose-1-phosphate guanyltransferase beta	○	○	○	○	○	○	○	○	○	○	○	○	○	○	○	●	○	○	○	○	○	○	○	○	●	●	○	○	○	○	○
SMIM22	K7EJ46	Small integral membrane protein 22	○	○	○	○	○	○	○	○	○	○	○	○	○	○	○	●	●	○	○	○	●	○	○	○	○	○	○	○	○	○	○
SLC5A1	P13866	Sodium/glucose cotransporter 1	○	○	○	○	○	○	○	○	○	○	○	○	○	○	○	○	●	○	○	○	○	○	●	○	○	○	○	○	●	○	○
CD9	P21926	CD9 antigen	○	○	○	○	○	○	○	○	○	○	○	○	○	○	○	○	○	○	○	○	○	○	○	○	○	●	○	○	●	●	○
FLOT1	O75955	Flotillin-1	○	○	○	○	○	○	○	○	○	○	○	○	○	○	○	●	●	○	○	○	○	○	○	○	○	○	○	○	●	○	○
ABI1	Q8IZP0	Abl interactor 1	○	○	○	○	○	○	○	○	○	○	○	○	○	○	○	○	●	○	○	○	○	○	○	○	○	○	○	○	●	○	●
STAM2	O75886	Signal transducing adapter molecule 2	○	○	○	○	○	○	●	○	○	○	○	●	○	○	○	○	○	○	○	○	○	○	○	○	○	○	○	○	○	○	○
BRK1	Q8WUW1	Protein BRICK1	○	○	○	○	○	○	○	○	○	○	○	○	○	○	○	●	○	○	○	○	○	○	○	○	○	○	○	○	○	○	●
PALM	O75781	Paralemmin-1	○	○	○	○	○	○	○	○	○	○	○	○	○	○	○	●	○	○	○	○	○	○	○	●	○	○	○	○	○	○	○
VPS37C	A5D8V6	Vacuolar protein sorting-associated protein 37C	○	○	○	○	○	○	○	○	○	○	○	○	○	○	○	○	○	○	●	○	○	○	○	○	○	○	○	○	●	○	○
RAB18	Q9NP72	Ras-related protein Rab-18	○	○	○	○	○	○	○	○	○	○	○	○	○	○	○	○	○	○	○	○	○	○	●	○	●	○	○	○	○	○	○
S100A9	P06702	Protein S100-A9	○	○	○	○	○	○	○	○	○	○	○	○	○	○	○	○	○	●	○	○	○	○	○	○	○	○	○	○	○	●	○
PTGR1	Q14914	Prostaglandin reductase 1	○	○	○	○	○	○	○	○	○	○	○	○	○	○	○	○	○	○	○	○	○	○	○	○	○	○	○	○	●	●	○
ARF5	P84085	ADP-ribosylation factor 5	○	○	○	○	○	○	○	○	○	○	○	○	○	○	○	○	○	○	○	○	○	○	●	●	○	○	○	○	○	○	○
GP2	P55259	Pancreatic secretory granule membrane major glycoprotein GP2	○	○	○	○	○	○	○	○	○	○	○	○	○	○	○	○	○	●	●	○	○	○	○	○	○	○	○	○	○	○	○
IDH1	O75874	Isocitrate dehydrogenase [NADP] cytoplasmic	○	○	○	○	○	○	○	○	○	○	○	○	○	○	○	○	○	○	○	○	○	○	●	○	○	○	○	○	●	○	○
PLCG2	P16885	1-phosphatidylinositol 4.5-bisphosphate phosphodiesterase gamma-2	○	○	○	○	○	○	●	○	○	○	○	○	○	○	○	○	○	○	○	○	○	○	○	○	○	○	○	○	○	○	○
GCA	P28676	Grancalcin	○	○	○	○	○	○	○	○	○	○	○	○	○	○	○	○	○	○	○	○	○	○	○	○	○	○	○	○	○	●	○
MICALL1	Q8N3F8	MICAL-like protein 1	○	○	○	○	○	●	○	○	○	○	○	○	○	○	○	○	○	○	○	○	○	○	○	○	○	○	●	○	○	○	○
GLTP	Q9NZD2	Glycolipid transfer protein	○	○	○	○	○	○	○	○	○	○	○	○	○	○	○	○	○	○	○	○	○	○	○	○	○	○	○	○	●	○	○
TPM4	P67936	Tropomyosin alpha-4 chain	○	○	○	○	○	○	○	○	○	○	○	○	○	○	○	○	○	○	○	○	○	○	○	○	○	○	○	○	●	○	○
GSTP1	P09211	Glutathione S-transferase P	○	○	○	○	○	○	○	○	○	○	○	○	○	○	○	○	○	○	○	○	○	○	●	○	○	○	○	○	○	○	○
AQP2	P41181	Aquaporin-2	○	○	○	○	○	○	○	○	○	○	○	○	○	○	○	○	●	○	○	○	○	○	○	○	○	○	○	○	○	○	○
SLC11A2	P49281	Natural resistance-associated macrophage protein 2	○	○	○	○	○	○	○	○	○	○	○	○	○	○	○	○	○	○	○	○	○	○	●	○	○	○	○	○	○	○	○
LAMP2	P13473	Lysosome-associated membrane glycoprotein 2	○	○	○	○	○	○	○	○	○	○	○	○	○	○	○	○	○	○	○	○	○	○	●	○	○	○	○	○	○	○	○
ABHD14B	Q96IU4	Alpha/beta hydrolase domain-containing protein 14B	○	○	○	○	○	○	○	○	○	○	○	○	○	○	○	○	○	○	○	○	○	○	○	○	○	○	○	○	○	○	○
CRCT1	Q9UGL9	Cysteine-rich C-terminal protein 1	○	○	○	○	○	○	○	○	○	○	○	○	○	○	○	○	○	○	○	○	○	○	○	○	○	○	○	○	○	○	○
LCK	P06239	Tyrosine-protein kinase Lck	○	○	○	○	○	○	○	○	○	○	○	○	○	○	○	○	○	○	○	○	○	○	○	○	○	○	○	○	○	○	○
ZDHHC1	Q8WTX9	Probable palmitoyltransferase ZDHHC1	○	○	○	○	○	○	○	○	○	○	○	○	○	○	○	○	○	○	○	○	○	○	○	○	○	○	○	○	○	○	○
SERPINA5	P05154	Plasma serine protease inhibitor	○	○	○	○	○	○	○	○	○	○	○	○	○	○	○	○	○	○	○	○	○	○	○	○	○	○	○	○	○	○	○

TABLE S5 Heatmap for enriched proteins at maximum combined specificity and sensitivity

Gene Name	Uniprot Entry	Identified Proteins	C1	C2	C3	C4	C5	C6	C7	C8	C9	C10	C11	C12	C13	C14	C15	P1	P2	P3	P4	P5	P6	P7	P8	P9	P10	P12	P13	P14	P15	P16	P17	Combined Specificity + Sensitivity
TMEM256	Q8N2U0	Transmembrane protein 256	○	○	○	○	○	○	○	○	○	○	○	○	○	○	○	●	●	●	●	●	●	●	●	●	●	●	●	○	●	●	●	194 %
ADIRF	Q15847	Adipogenesis regulatory factor	○	○	○	○	○	○	○	○	○	○	○	○	○	○	○	●	●	●	●	●	●	●	○	●	●	●	○	○	●	●	●	181 %
LAMTOR1	Q6IAA8	Ragulator complex protein LAMTOR1	○	○	○	○	○	○	○	○	○	○	○	○	○	○	○	●	●	○	●	●	●	●	●	●	○	●	●	●	●	○	●	181 %
RAB7A	P51149	Ras-related protein Rab-7a	●	○	○	○	○	○	●	○	○	○	○	○	○	○	○	●	●	●	●	○	●	●	●	●	●	●	●	●	●	●	●	180 %
UBE2V2	Q15819	Ubiquitin-conjugating enzyme E2 variant 2	○	○	●	○	○	○	○	○	○	○	○	●	○	○	○	●	●	●	●	●	●	●	●	●	●	●	○	●	●	●	●	180 %
VAMP2	P63027	Vesicle-associated membrane protein 2	●	○	●	○	○	●	○	○	○	○	○	○	○	○	○	●	●	●	●	●	●	●	●	●	●	●	●	●	●	●	●	180 %
ATP6V0C	P27449	V-type proton ATPase 16 kDa proteolipid subunit	○	○	○	○	○	○	○	○	○	○	○	○	○	○	○	●	●	●	●	●	●	●	○	●	●	●	○	●	○	○	●	175 %
LCP1	P13796	Plastin-2	○	○	○	○	○	○	○	○	○	○	○	○	○	○	○	●	●	○	●	●	○	●	●	●	●	●	○	○	●	●	●	175 %
PCYOX1	Q9UHG3	Prenylcysteine oxidase 1	○	○	○	○	○	○	●	○	○	○	○	○	○	○	○	●	●	●	○	●	●	●	●	●	●	●	○	○	●	●	●	175 %
SRI	P30626	Sorcin	○	○	●	○	●	○	○	○	○	○	○	○	○	○	○	●	●	●	●	●	●	●	●	●	●	●	○	○	●	●	●	174 %
RAB3D	O95716	Ras-related protein Rab-3D	○	○	○	○	○	○	○	○	●	○	○	●	○	○	○	●	●	●	●	○	●	●	●	●	●	●	●	○	●	●	●	174 %
PARK7	Q99497	Protein DJ-1	○	○	○	●	○	○	○	○	●	○	●	○	○	○	○	●	●	●	●	●	○	●	●	●	●	●	●	●	●	●	●	174 %
TSPAN6	O43657	Tetraspanin-6	○	○	○	●	○	○	○	○	○	○	●	○	○	○	●	●	●	●	●	●	●	●	○	●	●	●	●	●	●	●	●	174 %
GCA	P28676	Grancalcin	●	○	●	○	●	○	○	○	○	○	○	○	○	○	○	●	●	●	●	●	●	●	●	●	●	●	●	○	●	●	●	174 %
ASAH1	Q13510	Acid ceramidase	○	●	○	○	○	○	○	○	○	○	○	○	○	○	○	●	●	●	●	○	●	●	●	●	○	○	○	●	●	●	●	168 %
FLOT2	Q14254	Flotillin-2	○	○	●	○	○	○	○	○	○	○	○	○	○	○	○	●	●	●	●	○	●	●	●	●	●	●	○	○	●	○	●	168 %
EFHD2	Q96C19	EF-hand domain-containing protein D2	●	○	○	○	○	○	○	○	○	○	○	●	○	○	○	●	●	●	●	○	●	●	●	○	●	○	●	●	●	●	●	168 %
RAB3B	P20337	Ras-related protein Rab-3B	○	○	○	○	○	○	○	○	○	○	●	●	○	○	○	●	●	●	●	○	●	●	●	○	●	●	●	○	●	●	●	168 %
PTGR1	Q14914	Prostaglandin reductase 1	●	○	○	○	○	○	○	○	○	○	●	●	○	○	○	●	●	○	●	●	●	●	●	●	●	●	○	●	●	●	●	168 %
VDAC1	P21796	Voltage-dependent anion-selective channel protein 1	○	●	○	●	○	○	●	○	○	○	○	○	○	○	○	●	●	●	●	●	●	○	●	●	●	●	●	○	●	●	●	168 %
DSTN	P60981	Destrin	○	○	○	○	○	○	○	○	○	●	●	●	○	○	○	●	●	●	○	○	●	●	●	●	●	●	●	●	●	●	●	168 %
FLOT1	O75955	Flotillin-1	●	○	●	○	○	○	●	○	●	○	○	○	○	○	○	●	●	●	●	●	●	●	●	●	●	●	●	○	●	●	●	167 %
RBP5	P82980	Retinol-binding protein 5	○	○	○	○	○	○	●	●	○	●	○	●	○	○	○	●	●	○	●	●	●	●	●	●	●	●	●	●	●	●	●	167 %
APRT	P07741	Adenine phosphoribosyltransferase	○	●	●	○	○	○	○	●	○	○	●	○	○	○	○	●	●	●	●	●	●	●	●	●	●	●	●	○	●	●	●	167 %
CD81	P60033	CD81 antigen	○	○	●	○	●	○	○	●	○	○	●	○	○	○	○	●	●	●	●	●	●	●	○	●	●	●	●	●	●	●	●	167 %
MMP24	Q9Y5R2	Matrix metalloproteinase-24	●	●	●	●	●	○	●	●	●	●	●	●	●	●	●	○	○	○	●	○	●	●	●	○	○	○	○	●	○	○	○	165 %
ITIH4	Q14624	Inter-alpha-trypsin inhibitor heavy chain H4	●	○	○	●	○	●	●	●	●	○	○	○	○	○	○	○	○	○	○	○	○	○	○	○	○	○	○	○	○	○	○	164 %
RAB14	P61106	Ras-related protein Rab-14	○	○	○	○	○	○	○	○	○	○	○	○	○	○	○	●	●	●	●	○	●	○	●	○	○	○	●	○	●	●	●	163 %
CYB561	P49447	Cytochrome b561	●	○	○	○	○	○	○	○	○	○	○	○	○	○	○	●	●	●	●	●	●	●	○	○	●	●	○	○	○	●	●	162 %
GMDS	O60547	GDP-mannose 4.6 dehydratase	○	○	○	○	○	○	○	○	○	○	●	○	○	○	○	●	○	○	●	●	○	●	●	○	●	●	●	●	●	●	○	162 %
ARL8B	Q9NVJ2	ADP-ribosylation factor-like protein 8B	○	○	○	○	○	○	●	○	○	○	○	○	○	○	○	●	●	○	●	○	●	●	●	●	●	●	○	○	●	○	●	162 %
MFSD12	Q6NUT3	Major facilitator superfamily domain-containing protein 12	○	○	●	○	○	○	○	○	○	○	○	○	○	○	○	●	●	●	●	●	●	●	●	●	○	●	○	○	○	○	●	162 %
SLC11A2	P49281	Natural resistance-associated macrophage protein 2	○	○	○	○	○	○	○	○	○	○	○	●	○	○	○	●	●	●	●	○	○	○	●	●	●	●	○	●	●	●	○	162 %
BDH2	Q9BUT1	3-hydroxybutyrate dehydrogenase type 2	○	○	●	○	○	○	○	○	○	●	○	○	○	○	○	●	●	○	●	○	●	○	●	●	●	●	○	●	●	●	●	162 %
CLDN2	P57739	Claudin-2	○	○	●	○	○	○	●	○	○	○	○	○	○	○	○	●	●	●	●	●	●	●	○	●	○	●	○	○	●	●	●	162 %
GSS	P48637	Glutathione synthetase	○	●	○	○	○	○	○	○	○	○	○	●	○	○	○	●	●	○	●	○	○	●	●	●	●	●	●	●	●	●	○	162 %
CALM1	P62158	Calmodulin	●	○	●	○	○	○	●	○	○	○	○	○	○	○	○	●	●	●	●	●	●	●	●	●	○	●	○	○	●	●	●	161 %
AKAP7	O43687	A-kinase anchor protein 7 isoforms alpha and beta	●	○	○	○	○	○	○	○	●	○	○	○	○	○	●	●	●	●	●	●	○	●	○	●	●	●	●	○	●	●	●	161 %
ABRACL	Q9P1F3	Costars family protein ABRACL	○	●	○	○	○	○	○	○	○	●	●	○	○	○	○	●	●	●	●	○	○	●	●	●	●	●	○	●	●	●	●	161 %
VPS25	Q9BRG1	Vacuolar protein-sorting-associated protein 25	●	○	●	○	○	○	●	○	○	○	○	○	○	○	○	●	●	●	●	●	●	●	●	●	○	●	○	○	●	●	●	161 %
ARPC4	P59998	Actin-related protein 2/3 complex subunit 4	●	○	●	●	○	○	○	○	○	○	○	○	○	○	○	●	●	●	●	○	●	●	●	●	●	○	●	○	●	●	●	161 %
QDPR	P09417	Dihydropteridine reductase	○	●	●	●	○	○	○	○	○	○	○	○	○	○	○	●	○	○	●	○	●	●	●	●	●	●	●	●	●	●	●	161 %
DDAH1	O94760	N(G).N(G)-dimethylarginine dimethylaminohydrolase 1	○	●	●	○	○	○	○	○	○	○	○	●	○	○	○	●	●	○	●	○	●	●	●	●	●	●	○	●	●	●	●	161 %
SERPINA5	P05154	Plasma serine protease inhibitor	○	●	●	●	●	●	●	●	●	●	●	●	●	●	●	○	○	●	○	○	○	○	●	●	○	○	○	●	●	○	●	161 %
CTSD	P07339	Cathepsin D	●	○	○	●	○	○	●	○	○	○	○	○	●	○	○	●	●	●	●	●	●	●	●	●	●	●	○	●	●	○	●	161 %
GMPPB	Q9Y5P6	Mannose-1-phosphate guanyltransferase beta	○	○	○	●	○	●	○	○	●	○	●	○	○	○	○	●	●	●	●	●	●	●	●	●	●	●	●	○	●	●	○	161 %
PNP	P00491	Purine nucleoside phosphorylase	●	○	●	○	○	○	●	○	○	●	○	○	○	○	○	●	●	○	●	●	●	●	●	●	●	●	○	●	●	●	●	161 %
SMIM22	K7EJ46	Small integral membrane protein 22	●	○	●	○	●	○	●	○	○	○	○	○	○	○	○	●	●	●	●	●	●	●	●	●	●	●	○	○	●	●	●	161 %
S100P	P25815	Protein S100-P	●	○	○	○	○	○	○	●	●	○	●	○	○	○	○	●	●	●	●	●	●	●	●	○	●	●	○	●	●	●	●	161 %
NAPSA	O96009	Napsin-A	○	●	○	○	○	○	●	○	○	●	○	●	○	○	○	●	●	●	●	○	●	●	●	●	●	●	●	●	●	○	●	161 %
TSPAN8	P19075	Tetraspanin-8	○	○	●	○	○	○	○	○	●	○	●	●	○	○	○	○	●	●	●	●	●	●	●	●	●	●	●	●	●	●	○	161 %
DCXR	Q7Z4W1	L-xylulose reductase	○	●	○	○	○	○	●	○	○	○	●	●	○	○	○	●	●	○	●	●	○	●	●	●	●	●	●	●	●	●	●	161 %
UGDH	O60701	UDP-glucose 6-dehydrogenase	○	○	○	●	○	●	○	○	●	○	●	○	○	○	○	●	●	●	●	●	●	●	●	○	●	●	●	○	●	●	●	161 %
ANXA4	P09525	Annexin A4	○	○	●	○	●	○	●	○	○	○	○	●	○	○	○	●	●	●	●	●	●	●	●	●	●	●	○	○	●	●	●	161 %
TMPRSS2	O15393	Transmembrane protease serine 2	○	●	○	○	○	○	○	○	●	○	●	●	○	○	○	●	●	●	●	●	●	●	●	●	●	●	○	○	●	●	●	161 %
TMEM63A	O94886	Transmembrane protein 63A	○	●	○	○	○	●	○	○	○	○	●	●	○	○	●	●	●	●	●	●	●	●	●	●	●	●	●	●	●	●	○	160 %
SLC16A4	O15374	Monocarboxylate transporter 5	○	○	○	○	○	○	○	○	○	○	○	○	○	○	○	●	●	○	●	●	○	○	○	●	○	●	●	○	●	○	●	156 %
SPR	P35270	Sepiapterin reductase	○	○	○	○	○	○	○	○	○	○	○	○	○	○	○	●	●	○	●	○	○	○	●	○	●	●	●	○	●	●	○	156 %
FKBP1A	P62942	Peptidyl-prolyl cis-trans isomerase FKBP1A	○	○	○	○	○	○	○	○	○	○	○	○	○	○	○	●	●	○	●	○	○	○	●	○	○	●	○	●	●	●	●	156 %
MLPH	Q9BV36	Melanophilin	○	○	○	○	○	○	○	○	○	○	○	○	○	○	○	●	○	○	●	○	○	●	●	○	●	●	●	○	●	●	○	156 %
TPM4	P67936	Tropomyosin alpha-4 chain	○	○	●	○	○	○	○	○	○	○	○	○	○	○	○	●	●	●	●	○	○	○	●	●	○	●	○	○	●	●	●	156 %
GLTP	Q9NZD2	Glycolipid transfer protein	○	○	●	○	○	○	○	○	○	○	○	○	○	○	○	●	●	●	●	○	●	●	○	○	○	●	○	○	●	●	●	156 %
GOLPH3	Q9H4A6	Golgi phosphoprotein 3	○	○	●	○	○	○	○	○	○	○	○	○	○	○	○	●	●	○	●	●	○	●	●	○	●	●	○	○	●	●	○	156 %
MYO5C	Q9NQX4	Unconventional myosin-Vc	○	○	○	○	○	●	○	○	○	○	○	○	○	○	○	●	○	○	●	●	○	●	●	○	●	●	●	○	●	●	○	156 %
GMFB	P60983	Glia maturation factor beta	●	○	○	○	○	○	○	○	○	○	○	○	○	○	○	●	●	●	○	○	○	●	●	●	○	○	●	○	●	●	●	156 %
STXBP4	Q6ZWJ1	Syntaxin-binding protein 4	○	○	●	○	○	○	○	○	○	○	○	○	○	○	○	●	●	○	●	○	○	●	●	●	○	○	○	●	●	●	●	156 %
ALDH1A3	P47895	Aldehyde dehydrogenase family 1 member A3	○	○	○	○	○	●	○	○	○	○	○	○	○	○	○	○	○	●	●	●	○	●	●	○	●	●	●	○	●	●	○	156 %
SCARB2	Q14108	Lysosome membrane protein 2	○	○	○	○	○	○	○	○	○	○	○	●	○	○	○	●	●	○	○	○	○	●	●	●	●	●	○	○	●	●	●	156 %
RAB8A	P61006	Ras-related protein Rab-8A	○	○	●	○	○	○	○	○	○	○	○	○	○	○	○	●	●	●	●	○	●	○	●	○	○	○	●	○	●	●	●	156 %
RAB35	Q15286	Ras-related protein Rab-35	○	○	●	○	○	○	○	○	○	○	○	○	○	○	○	●	●	●	●	○	●	○	●	○	○	○	●	○	●	●	●	156 %
RAB8B	Q92930	Ras-related protein Rab-8B	○	○	●	○	○	○	○	○	○	○	○	○	○	○	○	●	●	●	●	○	●	○	●	○	○	○	●	○	●	●	●	156 %
RAB10	P61026	Ras-related protein Rab-10	○	○	●	○	○	○	○	○	○	○	○	○	○	○	○	●	●	●	●	○	●	○	●	○	○	○	●	○	●	●	●	156 %
CD59	P13987	CD59 glycoprotein	●	○	●	○	○	○	○	○	○	○	○	○	○	○	○	●	●	○	●	●	○	●	●	○	○	●	●	○	●	●	●	155 %
SLC34A2	O95436	Sodium-dependent phosphate transport protein 2B	○	○	●	○	○	○	○	○	●	○	○	○	○	○	○	●	●	●	●	○	●	○	●	●	○	○	●	○	●	●	●	155 %
SLC2A13	Q96QE2	Proton myo-inositol cotransporter	○	○	○	○	○	○	○	○	●	○	○	○	●	○	○	●	●	●	○	●	○	●	○	●	●	●	●	○	●	○	●	155 %
HGSNAT	Q68CP4	Heparan-alpha-glucosaminide N-acetyltransferase	○	○	●	○	○	○	○	○	●	○	○	○	○	○	○	●	●	●	●	○	●	●	●	●	●	○	○	○	●	○	●	155 %
LTF	P02788	Lactotransferrin	○	●	○	●	○	○	○	○	○	○	○	○	○	○	○	○	○	●	●	●	●	●	●	○	●	●	○	●	●	●	○	155 %
SARG	Q9BW04	Specifically androgen-regulated gene protein	○	○	○	○	○	○	●	○	●	○	○	○	○	○	○	●	●	○	●	○	○	●	●	●	●	●	○	○	●	●	●	155 %
VPS37C	A5D8V6	Vacuolar protein sorting-associated protein 37C	○	○	●	○	○	○	●	○	○	○	○	○	○	○	○	●	○	●	●	○	●	●	●	●	●	●	○	○	●	●	○	155 %
RAB27B	O00194	Ras-related protein Rab-27B	○	○	○	○	○	○	○	○	○	○	●	●	○	○	○	●	●	○	●	○	○	○	●	●	●	●	●	●	●	●	○	155 %
NIT2	Q9NQR4	Omega-amidase NIT2	○	○	●	○	○	○	○	○	○	○	●	○	○	○	○	●	●	○	●	○	○	●	●	●	●	●	○	○	●	●	●	155 %

RAB1A	P62820	Ras-related protein Rab-1A	155 %
RAB1B	Q9H0U4	Ras-related protein Rab-1B	155 %
ATP6V0D1	P61421	V-type proton ATPase subunit d 1	155 %
CRCT1	Q9UGL9	Cysteine-rich C-terminal protein 1	155 %
DNPH1	O43598	2'-deoxynucleoside 5'-phosphate N-hydrolase 1	155 %
SYTL4	Q96C24	Synaptotagmin-like protein 4	155 %
TM7SF3	Q9NS93	Transmembrane 7 superfamily member 3	155 %
ARF5	P84085	ADP-ribosylation factor 5	155 %
BLVRB	P30043	Flavin reductase (NADPH)	155 %
SYT7	O43581	Synaptotagmin-7	155 %
STEAP4	Q687X5	Metalloreductase STEAP4	155 %
ARPC5	O15511	Actin-related protein 2/3 complex subunit 5	155 %
IFITM3	Q01628	Interferon-induced transmembrane protein 3	154 %
NCSTN	Q92542	Nicastrin	154 %
PPAP2A	O14494	Lipid phosphate phosphohydrolase 1	154 %
RAB18	Q9NP72	Ras-related protein Rab-18	154 %
CLDN10	P78369	Claudin-10	154 %
TPT1	P13693	Translationally-controlled tumor protein	154 %
HEBP2	Q9Y5Z4	Heme-binding protein 2	154 %
LAMP2	P13473	Lysosome-associated membrane glycoprotein 2	154 %
FOLH1	Q04609	Glutamate carboxypeptidase 2	154 %
ACTN1	P12814	Alpha-actinin-1	150 %
LAPTM4A	Q15012	Lysosomal-associated transmembrane protein 4A	150 %
LRSAM1	Q6UWE0	E3 ubiquitin-protein ligase LRSAM1	150 %
PHYHD1	Q5SRE7	Phytanoyl-CoA dioxygenase domain-containing protein 1	150 %
PSMA5	P28066	Proteasome subunit alpha type-5	150 %
CPQ	Q9Y646	Carboxypeptidase Q	150 %
HLA-DMA	P28067	HLA class II histocompatibility antigen. DM alpha chain	150 %
CLDN3	O15551	Claudin-3	150 %
RAB12	Q6IQ22	Ras-related protein Rab-12	150 %
GPR155	Q7Z3F1	Integral membrane protein GPR155	150 %
CLN3	Q13286	Battenin	150 %
P2RX4	Q99571	P2X purinoceptor 4	150 %
NQO2	P16083	Ribosyldihydronicotinamide dehydrogenase [quinone]	150 %
CUTA	O60888	Protein CutA	150 %
DBI	P07108	Acyl-CoA-binding protein	150 %
RAB9A	P51151	Ras-related protein Rab-9A	150 %
LGALS3BP	Q08380	Galectin-3-binding protein	150 %
GNPDA1	P46926	Glucosamine-6-phosphate isomerase 1	149 %
IGHA1	P01876	Ig alpha-1 chain C region	149 %
ALAD	P13716	Delta-aminolevulinic acid dehydratase	149 %
TMEM176A	Q96HP8	Transmembrane protein 176A	149 %
SCAMP1	O15126	Secretory carrier-associated membrane protein 1	149 %
QPRT	Q15274	Nicotinate-nucleotide pyrophosphorylase [carboxylating]	149 %
CALB1	P05937	Calbindin	149 %
ATP8A1	Q9Y2Q0	Probable phospholipid-transporting ATPase IA	149 %
CLIC3	O95833	Chloride intracellular channel protein 3	149 %
STAM2	O75886	Signal transducing adapter molecule 2	149 %
ZDHHC1	Q8WTX9	Probable palmitoyltransferase ZDHHC1	149 %
ACP1	P24666	Low molecular weight phosphotyrosine protein phosphatase	149 %
CTTN	Q14247	Src substrate cortactin	149 %
SLC35F6	Q8N357	Solute carrier family 35 member F6	149 %
CRYZ	Q08257	Quinone oxidoreductase	149 %
SFN	P31947	14-3-3 protein sigma	149 %
EIF4H	Q15056	Eukaryotic translation initiation factor 4H	149 %
ANXA3	P12429	Annexin A3	149 %
DYNLL2	Q96FJ2	Dynein light chain 2. cytoplasmic	149 %
FAM129A	Q9BZQ8	Protein Niban	149 %
SLC5A1	P13866	Sodium/glucose cotransporter 1	149 %
SLC44A4	Q53GD3	Choline transporter-like protein 4	149 %
YWHAQ	P27348	14-3-3 protein theta	149 %
CAB39	Q9Y376	Calcium-binding protein 39	149 %
RAB2A	P61019	Ras-related protein Rab-2A	148 %
RAB6A	P20340	Ras-related protein Rab-6A	148 %
SPRR1A	P35321	Cornifin-A	148 %
COL6A1	P12109	Collagen alpha-1(VI) chain	148 %
SKP1	P63208	S-phase kinase-associated protein 1	148 %
MAPK1	P28482	Mitogen-activated protein kinase 1	148 %
ADH5	P11766	Alcohol dehydrogenase class-3	148 %
CDC42	P60953	Cell division control protein 42 homolog	148 %
IDH1	O75874	Isocitrate dehydrogenase [NADP] cytoplasmic	148 %
ABI1	Q8IZP0	Abl interactor 1	148 %
GSTM3	P21266	Glutathione S-transferase Mu 3	148 %
SCAMP2	O15127	Secretory carrier-associated membrane protein 2	144 %
TPD52	P55327	Tumor protein D52	144 %
CPVL	Q9H3G5	Probable serine carboxypeptidase CPVL	144 %
ENOPH1	Q9UHY7	Enolase-phosphatase E1	144 %
PSEN1	P49768	Presenilin-1	144 %
LAMTOR5	O43504	Ragulator complex protein LAMTOR5	144 %
PLCG2	P16885	1-phosphatidylinositol 4.5-bisphosphate phosphodiesterase gamma-2	144 %
PSMA4	P25789	Proteasome subunit alpha type-4	143 %
S100A6	P06703	Protein S100-A6	143 %
GOT1	P17174	Aspartate aminotransferase. cytoplasmic	143 %
HDHD2	Q9H0R4	Haloacid dehalogenase-like hydrolase domain-containing protein 2	143 %
BRK1	Q8WUW1	Protein BRICK1	143 %
TMSB4X	P62328	Thymosin beta-4	143 %
GSTP1	P09211	Glutathione S-transferase P	143 %

B2M	P61769	Beta-2-microglobulin	○	○	○	○	○	○	○	●	○	○	○	○	○	○	○	●	●	○	○	○	○	○	●	●	○	●	○	○	●	●	●	143 %
RAB17	Q9H0T7	Ras-related protein Rab-17	○	○	●	○	○	○	○	○	○	○	○	○	○	○	○	●	●	○	○	○	○	○	●	●	●	●	○	○	●	○	●	143 %
LAMP1	P11279	Lysosome-associated membrane glycoprotein 1	○	○	○	○	○	○	●	○	○	○	○	○	○	○	○	●	●	●	○	○	○	○	●	○	●	○	○	○	●	●	●	143 %
CDC42SE2	Q9NRR3	CDC42 small effector protein 2	○	○	●	○	○	○	●	○	○	○	○	○	○	○	○	●	●	○	●	○	●	○	●	●	○	○	○	●	●	○	●	143 %
TMBIM1	Q969X1	Protein lifeguard 3	●	○	○	○	○	○	○	○	○	○	○	●	○	○	○	●	●	●	○	○	○	○	●	●	○	●	○	○	●	●	●	143 %
TUBB2B	Q9BVA1	Tubulin beta-2B chain	○	○	○	●	○	●	○	○	○	○	○	○	○	○	○	●	●	○	●	○	○	●	○	○	○	●	●	●	●	●	○	143 %
DMBT1	Q9UGM3	Deleted in malignant brain tumors 1 protein	●	○	○	○	○	○	○	○	●	○	○	○	○	○	○	●	○	●	●	○	●	●	○	○	○	●	●	●	○	○	●	143 %
SMIM5	Q71RC9	Small integral membrane protein 5	○	○	○	○	●	○	●	○	○	○	○	○	○	○	○	●	●	●	●	○	●	●	○	●	○	●	○	○	○	○	●	143 %
PSMA7	O14818	Proteasome subunit alpha type-7	○	○	○	○	○	○	●	○	○	○	●	○	○	○	○	○	○	○	●	●	○	●	●	○	●	●	●	○	●	●	○	143 %
TXNDC17	Q9BRA2	Thioredoxin domain-containing protein 17	○	○	○	○	○	○	○	●	○	○	●	○	○	○	○	●	●	○	●	○	○	●	●	●	●	○	○	○	●	●	○	143 %
AQP2	P41181	Aquaporin-2	○	○	●	○	○	○	○	○	○	●	○	○	○	○	○	○	●	○	●	○	●	●	●	○	○	○	●	○	●	●	●	143 %
PALM	O75781	Paralemmin-1	○	○	○	○	○	○	●	○	○	○	○	●	○	○	○	●	●	○	●	○	○	○	○	●	○	●	○	●	●	●	●	143 %
LYZ	P61626	Lysozyme C	○	○	○	○	○	○	○	●	○	○	○	●	●	○	○	●	●	○	●	○	●	●	○	●	○	●	○	●	●	○	●	143 %
ARSF	P54793	Arylsulfatase F	○	●	○	○	○	○	●	○	●	○	○	○	○	○	○	●	●	○	●	○	●	○	●	●	○	●	○	○	●	●	●	143 %
AZGP1	P25311	Zinc-alpha-2-glycoprotein	○	○	○	○	○	●	○	○	○	○	●	●	○	○	○	○	○	○	●	○	○	●	●	●	●	●	●	●	●	●	○	143 %
ZNF185	O15231	Zinc finger protein 185	●	○	○	○	○	○	●	○	○	●	○	○	○	○	○	●	●	●	●	○	●	●	●	○	○	○	○	○	●	●	●	143 %
CTSZ	Q9UBR2	Cathepsin Z	○	●	○	●	○	○	●	○	○	○	○	○	○	○	○	●	●	○	○	○	●	●	●	●	○	●	○	●	●	○	●	143 %
MTPN	P58546	Myotrophin	○	○	○	●	○	○	●	●	○	○	●	○	○	○	○	●	●	●	●	●	○	●	●	●	●	●	○	○	●	○	○	142 %
AQP7	O14520	Aquaporin-7	●	○	○	○	○	○	●	○	●	○	○	○	○	●	○	●	●	○	●	●	●	●	●	●	●	○	○	○	○	●	●	142 %
PSAP	P07602	Proactivator polypeptide	●	○	●	●	○	○	●	○	○	○	○	○	○	○	○	●	●	●	●	○	●	●	○	○	●	●	○	○	●	●	●	142 %
S100A1	P23297	Protein S100-A1	●	○	●	○	●	○	●	○	○	○	○	○	○	○	○	●	●	○	●	●	●	●	●	○	○	●	○	○	●	●	●	142 %
GP2	P55259	Pancreatic secretory granule membrane major glycoprotein GP2	●	○	○	○	○	○	○	○	●	○	●	●	○	○	○	○	●	●	●	●	●	●	●	○	●	●	●	○	●	○	○	142 %
FABP5	Q01469	Fatty acid-binding protein. epidermal	○	●	○	○	○	○	○	○	○	●	○	○	○	●	●	●	●	○	●	●	●	●	○	●	●	●	○	●	○	●	○	142 %
PPP2CA	P67775	Serine/threonine-protein phosphatase 2A catalytic subunit alpha isoform	○	○	●	●	○	○	○	●	○	○	●	○	○	○	○	●	●	○	●	○	○	●	●	○	●	●	●	○	●	●	●	142 %
CCT5	P48643	T-complex protein 1 subunit epsilon	○	○	○	●	○	●	○	○	●	○	●	○	○	○	○	●	○	○	●	●	○	●	●	○	●	●	●	○	●	●	●	142 %
CLIC6	Q96NY7	Chloride intracellular channel protein 6	●	○	○	○	○	○	●	●	●	○	○	○	○	○	○	●	●	●	●	○	●	●	●	○	●	○	○	○	●	●	●	142 %
DNM2	P50570	Dynamin-2	○	○	●	○	○	○	●	●	○	○	●	○	○	○	○	●	●	●	●	○	○	●	●	●	●	○	○	○	●	●	●	142 %
HPCAL1	P37235	Hippocalcin-like protein 1	○	○	●	●	○	○	●	○	○	○	●	○	○	○	○	●	●	●	●	○	●	●	●	●	○	●	○	●	●	○	○	142 %
CRABP2	P29373	Cellular retinoic acid-binding protein 2	○	●	○	○	○	○	○	●	○	○	●	○	○	●	○	●	●	●	●	○	●	●	●	○	○	●	○	○	●	●	●	142 %
GSTM1	P09488	Glutathione S-transferase Mu 1	●	○	●	○	○	○	○	○	●	●	●	○	○	○	○	●	●	○	●	○	●	●	○	●	○	●	●	●	●	●	●	142 %
UPK1A	O00322	Uroplakin-1a	●	○	●	○	●	○	●	○	●	○	○	○	○	○	○	●	●	○	●	●	○	●	●	●	○	●	○	●	●	●	●	142 %
APOA1BP	Q8NCW5	NAD(P)H-hydrate epimerase	●	○	●	○	○	○	●	○	○	○	●	●	○	○	○	●	●	●	○	●	○	●	●	○	●	●	●	○	●	●	●	142 %
PNKD	Q8N490	Probable hydrolase PNKD	○	○	○	○	○	●	○	○	●	○	●	●	●	●	●	○	○	○	○	○	○	○	○	○	●	○	●	○	○	○	○	141 %
LCK	P06239	Tyrosine-protein kinase Lck	●	○	●	○	○	○	○	●	○	●	●	●	●	○	○	●	○	○	○	○	○	○	●	○	○	○	○	○	○	○	○	141 %
SMS	P52788	Spermine synthase	●	○	○	●	○	●	●	○	●	○	●	○	○	○	○	●	●	●	●	●	○	●	●	○	●	●	●	○	●	●	●	141 %
SEPTIN2	Q15019	Septin-2	○	●	●	○	○	○	●	●	●	○	●	○	○	○	○	●	●	●	●	○	○	●	●	●	●	○	●	●	●	●	●	141 %
HRAS	P01112	GTPase HRas	○	○	○	○	○	○	○	○	○	○	○	○	○	○	○	●	●	○	●	○	○	○	○	○	●	○	○	○	●	●	○	138 %
PTGR2	Q8N8N7	Prostaglandin reductase 2	○	○	○	○	○	○	○	○	○	○	○	○	○	○	○	○	●	○	○	○	○	○	●	○	○	●	●	○	●	●	○	138 %
LAMTOR2	Q9Y2Q5	Ragulator complex protein LAMTOR2	○	○	○	○	○	○	○	○	○	○	○	○	○	○	○	●	●	○	●	○	○	○	○	●	○	○	○	○	●	○	●	138 %
LAMTOR3	Q9UHA4	Ragulator complex protein LAMTOR3	○	○	○	○	○	○	○	○	○	○	○	○	○	○	○	●	●	○	○	○	○	○	○	●	○	●	○	○	●	○	●	138 %
SLC35F2	Q8IXU6	Solute carrier family 35 member F2	○	○	○	○	○	○	○	○	○	○	○	○	○	○	○	●	●	○	○	●	○	○	○	○	○	●	○	○	○	●	●	138 %
TMEM106B	Q9NUM4	Transmembrane protein 106B	○	○	○	○	○	○	○	○	○	○	○	○	○	○	○	●	●	○	○	○	○	○	●	●	○	●	○	○	○	○	●	138 %
C1orf85	Q8WWB7	Lysosomal protein NCU-G1	○	○	○	○	○	○	○	○	○	○	○	○	○	○	○	●	●	○	○	○	○	○	●	●	○	●	○	○	○	○	●	138 %
SPNS1	Q9H2V7	Protein spinster homolog 1	○	○	○	○	○	○	○	○	○	○	○	○	○	○	○	●	●	○	○	○	○	●	○	●	○	●	○	○	○	○	●	138 %
HLA-DRB1	P01911	HLA class II histocompatibility antigen. DRB1-15 beta chain	○	○	○	○	○	○	○	○	○	○	○	○	○	○	○	●	○	●	○	○	○	○	●	●	●	○	○	○	●	○	○	138 %
CALML3	P27482	Calmodulin-like protein 3	○	○	○	○	○	○	○	○	○	○	○	○	○	○	○	●	○	○	●	○	●	●	○	●	○	●	○	○	○	○	○	138 %
PMVK	Q15126	Phosphomevalonate kinase	○	○	○	○	○	○	○	○	○	○	○	○	○	○	○	○	○	○	○	○	○	○	●	●	●	●	●	○	●	○	○	138 %
PGM3	O95394	Phosphoacetylglucosamine mutase	○	○	○	○	○	○	○	○	○	○	○	●	○	○	○	○	○	○	○	●	○	○	●	○	●	●	●	○	●	●	○	137 %
TMEM8A	Q9HCN3	Transmembrane protein 8A	○	○	○	○	○	○	○	○	○	○	○	●	○	○	○	○	●	●	○	○	○	○	●	○	○	●	○	○	●	●	●	137 %
FTH1	P02794	Ferritin heavy chain	○	○	○	○	○	○	●	○	○	○	○	○	○	○	○	●	○	○	○	○	○	○	●	○	●	●	○	○	●	●	●	137 %
CD38	P28907	ADP-ribosyl cyclase 1	○	○	○	○	○	●	○	○	○	○	○	○	○	○	○	○	○	○	●	●	○	○	○	○	●	●	●	○	●	●	○	137 %
C6orf132	Q5T0Z8	Uncharacterized protein C6orf132	●	○	○	○	○	○	○	○	○	○	○	○	○	○	○	●	●	●	●	○	●	○	●	○	○	○	○	○	●	○	○	137 %
TMEM30A	Q9NV96	Cell cycle control protein 50A	○	○	○	○	○	○	○	○	○	○	●	○	○	○	○	●	○	●	○	○	○	●	●	○	●	○	○	○	●	○	●	137 %
SNCG	O76070	Gamma-synuclein	○	●	○	○	○	○	○	○	○	○	○	○	○	○	○	●	●	○	●	○	○	○	○	●	○	○	○	○	●	●	●	137 %
RAB43	Q86YS6	Ras-related protein Rab-43	○	○	○	●	○	●	○	○	○	○	○	○	○	○	○	●	●	○	○	○	○	○	●	●	○	●	○	○	●	●	●	137 %
NPDC1	Q9NQX5	Neural proliferation differentiation and control protein 1	○	○	○	○	○	●	○	○	●	○	○	○	○	○	○	○	○	○	●	●	○	○	●	○	○	●	●	●	●	●	○	137 %
TTYH3	Q9C0H2	Protein tweety homolog 3	○	○	○	○	○	○	●	○	○	○	○	●	○	●	○	●	●	●	○	○	○	●	●	○	○	●	○	○	●	●	●	136 %
HLA-DRB1	Q29974	HLA class II histocompatibility antigen. DRB1-16 beta chain	●	○	●	○	○	○	●	○	○	○	○	○	○	○	○	●	○	●	○	○	○	●	●	●	●	●	○	●	●	○	○	136 %
SPAG9	O60271	C-Jun-amino-terminal kinase-interacting protein 4	○	○	○	●	○	●	●	○	○	○	○	○	○	○	○	●	●	○	●	○	○	○	●	●	○	●	●	○	●	○	●	136 %
CD9	P21926	CD9 antigen	○	●	●	○	●	○	○	○	○	○	○	●	○	○	○	●	●	○	○	●	○	●	●	●	○	●	○	○	●	●	●	136 %
SERINC2	Q96SA4	Serine incorporator 2	○	○	●	○	○	○	●	●	●	○	○	○	○	○	○	●	●	●	●	○	○	○	●	●	○	●	○	○	●	●	●	136 %
ATP6V1F	Q16864	V-type proton ATPase subunit F	●	○	●	○	○	○	●	○	○	○	●	○	○	○	○	●	●	●	●	○	●	○	○	●	○	●	○	○	●	●	●	136 %
STAM	Q92783	Signal transducing adapter molecule 1	○	●	○	●	○	●	●	●	●	●	●	●	○	○	●	○	●	○	○	○	●	○	●	○	○	●	○	○	●	○	○	135 %
ABHD14B	Q96IU4	Alpha/beta hydrolase domain-containing protein 14B	○	○	○	●	○	○	●	○	●	○	○	●	○	●	○	●	●	○	○	○	○	●	●	●	●	●	○	●	●	●	●	135 %
MARCKS	P29966	Myristoylated alanine-rich C-kinase substrate	●	○	○	●	○	●	●	○	●	○	●	○	○	○	○	●	●	●	●	○	●	○	○	○	●	●	●	●	●	●	●	135 %
KLK3	P07288	Prostate-specific antigen	●	○	○	●	○	●	●	○	●	○	●	●	○	○	○	●	●	●	●	●	○	●	●	○	●	●	●	●	●	●	○	135 %
SYNGR2	O43760	Synaptogyrin-2	○	○	○	○	○	○	○	○	○	○	○	○	○	○	○	●	○	○	●	●	○	○	○	○	●	●	○	○	○	○	○	131 %
PSMB2	P49721	Proteasome subunit beta type-2	○	○	○	○	○	○	○	○	○	○	○	○	○	○	○	○	●	○	○	○	○	○	●	○	○	●	○	○	●	●	○	131 %
SLC15A1	P46059	Solute carrier family 15 member 1	○	○	○	○	○	○	○	○	○	○	○	○	○	○	○	●	○	○	○	●	○	○	●	●	○	●	○	○	○	○	○	131 %
UBE2K	P61086	Ubiquitin-conjugating enzyme E2 K	○	○	○	○	○	○	○	○	○	○	○	○	○	○	○	○	●	○	○	○	○	○	●	○	○	●	○	○	●	●	○	131 %
SI	P14410	Sucrase-isomaltase. intestinal	○	○	○	○	○	○	○	○	○	○	○	○	○	○	○	○	○	●	●	●	○	○	○	○	○	●	○	○	○	●	○	131 %
S100A9	P06702	Protein S100-A9	●	●	○	○	○	○	○	○	○	○	○	○	○	○	○	○	○	●	○	○	●	○	○	○	●	●	○	○	●	●	●	130 %
DOPEY2	Q9Y3R5	Protein dopey-2	○	○	○	○	○	●	○	○	○	○	●	●	○	○	●	●	●	○	●	○	○	○	●	○	●	●	●	○	●	●	○	130 %
TSTD1	Q8NFU3	Thiosulfate sulfurtransferase/rhodanese-like domain-containing protein 1	●	○	●	●	○	●	○	○	○	●	○	○	○	○	○	○	●	○	●	●	○	●	●	○	●	●	●	○	●	●	○	129 %
CEACAM6	P40199	Carcinoembryonic antigen-related cell adhesion molecule 6	○	○	○	○	○	○	○	○	○	○	○	○	○	○	○	●	○	●	○	○	●	○	●	○	○	●	○	○	○	○	○	118 %
IGLC2	P0CG05	Ig lambda-2 chain C regions	○	○	○	●	○	○	○	●	○	○	○	○	○	○	○	○	○	●	○	○	●	○	○	○	○	○	○	●	●	●	○	118 %
MICALL1	Q8N3F8	MICAL-like protein 1	○	○	○	○	○	○	○	○	●	○	○	○	○	○	○	○	○	○	○	○	○	○	○	●	○	○	●	○	○	○	●	112 %

Key:

Light blue		Unique for patient urinary exosomes
Light red		Enriched in control urinary exosomes
	●	Positive for given sample at threshold
	○	Negative for given sample at threshold
